# Associations Between Socioeconomic Status, Obesity, Cognition, and White Matter Microstructure in Children

**DOI:** 10.1001/jamanetworkopen.2023.20276

**Published:** 2023-06-27

**Authors:** Zhaolong Adrian Li, Yuqi Cai, Rita L. Taylor, Sarah A. Eisenstein, Deanna M. Barch, Scott Marek, Tamara Hershey

**Affiliations:** 1 Department of Psychiatry, Washington University in St Louis School of Medicine, St Louis, Missouri; 2 Department of Psychological and Brain Sciences, Washington University in St Louis, St Louis, Missouri; 3 Now with Ann Romney Center for Neurologic Diseases, Department of Neurology, Brigham and Women’s Hospital, Harvard Medical School, Boston, Massachusetts; 4 Mallinckrodt Institute of Radiology, Washington University in St Louis School of Medicine, St Louis, Missouri; 5 Department of Neurology, Washington University in St Louis School of Medicine, St Louis, Missouri

## Abstract

**Importance:**

Lower neighborhood and household socioeconomic status (SES) are associated with negative health outcomes and altered brain structure in children. It is unclear whether such findings extend to white matter and via what mechanisms.

**Objective:**

To assess whether and how neighborhood and household SES are independently associated with children’s white matter microstructure and examine whether obesity and cognitive performance (reflecting environmental cognitive and sensory stimulation) are plausible mediators.

**Design, Setting, and Participants:**

This cross-sectional study used baseline data from participants in the Adolescent Brain Cognitive Development (ABCD) study. Data were collected at 21 US sites, and school-based recruitment was used to represent the US population. Children aged 9 to 11 years and their parents or caregivers completed assessments between October 1, 2016, and October 31, 2018. After exclusions, 8842 of 11 875 children in the ABCD study were included in the analyses. Data analysis was conducted from July 11 to December 19, 2022.

**Exposures:**

Neighborhood disadvantage was derived from area deprivation indices at participants’ primary residence. Household SES factors were total income and highest parental educational attainment.

**Main Outcomes and Measures:**

A restriction spectrum imaging (RSI) model was used to quantify restricted normalized directional (RND; reflecting oriented myelin organization) and restricted normalized isotropic (RNI; reflecting glial and neuronal cell bodies) diffusion in 31 major white matter tracts. The RSI measurements were scanner harmonized. Obesity was assessed through body mass index (BMI; calculated as weight in kilograms divided by height in meters squared), age- and sex-adjusted BMI *z* scores, and waist circumference, and cognition was assessed through the National Institutes of Health Toolbox Cognition Battery. Analyses were adjusted for age, sex, pubertal development stage, intracranial volume, mean head motion, and twin or siblingship.

**Results:**

Among 8842 children, 4543 (51.4%) were boys, and the mean (SD) age was 9.9 (0.7) years. Linear mixed-effects models revealed that greater neighborhood disadvantage was associated with lower RSI-RND in the left superior longitudinal fasciculus (β = −0.055; 95% CI, −0.081 to −0.028) and forceps major (β = −0.040; 95% CI, −0.067 to −0.013). Lower parental educational attainment was associated with lower RSI-RND in the bilateral superior longitudinal fasciculus (eg, right hemisphere: β = 0.053; 95% CI, 0.025-0.080) and bilateral corticospinal or pyramidal tract (eg, right hemisphere: β = 0.042; 95% CI, 0.015-0.069). Structural equation models revealed that lower cognitive performance (eg, lower total cognition score and higher neighborhood disadvantage: β = −0.012; 95% CI, −0.016 to −0.009) and greater obesity (eg, higher BMI and higher neighborhood disadvantage: β = −0.004; 95% CI, −0.006 to −0.001) partially accounted for the associations between SES and RSI-RND. Lower household income was associated with higher RSI-RNI in most tracts (eg, right inferior longitudinal fasciculus: β = −0.042 [95% CI, −0.073 to −0.012]; right anterior thalamic radiations: β = −0.045 [95% CI, −0.075 to −0.014]), and greater neighborhood disadvantage had similar associations in primarily frontolimbic tracts (eg, right fornix: β = 0.046 [95% CI, 0.019-0.074]; right anterior thalamic radiations: β = 0.045 [95% CI, 0.018-0.072]). Lower parental educational attainment was associated with higher RSI-RNI in the forceps major (β = −0.048; 95% CI, −0.077 to −0.020). Greater obesity partially accounted for these SES associations with RSI-RNI (eg, higher BMI and higher neighborhood disadvantage: β = 0.015; 95% CI, 0.011-0.020). Findings were robust in sensitivity analyses and were corroborated using diffusion tensor imaging.

**Conclusions and Relevance:**

In this cross-sectional study, both neighborhood and household contexts were associated with white matter development in children, and findings suggested that obesity and cognitive performance were possible mediators in these associations. Future research on children’s brain health may benefit from considering these factors from multiple socioeconomic perspectives.

## Key Points

**Question:**

Are neighborhood and household socioeconomic status associated with children’s brain white matter microstructure and, if so, do obesity and cognitive performance (reflecting environmental stimulation) plausibly mediate the associations?

**Findings:**

In this cross-sectional study of 8842 children who participated in the Adolescent Brain Cognitive Development study, higher neighborhood disadvantage, lower household income, and lower parental educational attainment had independent associations with lower restricted directional diffusion and greater restricted isotropic diffusion in white matter. Greater body mass index and poorer cognitive performance partially explained these associations.

**Meaning:**

These findings suggest that future research on children’s brain health may benefit from considering obesity incidence and environmental cognitive enrichment from multiple socioeconomic perspectives.

## Teaser

This cross-sectional study uses data from the Adolescent Brain Cognitive Development study to assess the associations of neighborhood and household socioeconomic status with children’s white matter microstructure and examine whether obesity and cognitive performance mediate any associations.

## Introduction

Socioeconomic disadvantage (eg, poverty) during childhood robustly estimates poor physical and mental health across the lifespan.^1,2,3^ Evidence suggests that early neurobiological differences may mediate the association between socioeconomic status (SES) and both concurrent and long-term brain-supported functioning.^3,4,5,6,7,8^ Thus, it is important to understand how early-life SES might play a role in brain development.

In children, lower SES has been associated with smaller cortical and hippocampal volumes and less cortical surface area, all of which are gray matter regions.^9,10,11^ Limited research suggests an association between low SES and compromised white matter microstructure in children,^12,13,14,15,16,17^ but findings have been inconsistent regarding which tracts are implicated, likely due to small samples and heterogeneous SES measurements.^6,18^ White matter tracts, which are primarily myelin-ensheathed axonal bundles connecting distal gray matter regions, are integral to efficient information processing,^19^ and lower white matter integrity has been associated with poorer visuospatial abilities and greater psychopathological characteristics in youths.^20,21^ White matter undergoes extensive myelination and microstructural organization from childhood through young adulthood.^22^ Such protracted development potentially gives low SES a lengthy window to impinge on white matter microstructure and function.

Socioeconomic status may play a role in brain development by modulating intermediary factors that more directly impact brain health.^6,7^ Recent studies^23,24,25^ have found obesity and lower cognitive performance to mediate associations between SES, brain volumes, and functional connectivity in children. These studies interpreted obesity as a reflection of nutrition and exercise^24^ and cognition as an outcome of the social, sensory, and cognitive stimulation in the environment.^23,25^ In addition, the SES of both neighborhood (eg, area deprivation index [ADI]) and household (eg, income and parental educational attainment), although correlated, may be independently associated with brain outcomes.^11,24,25,26,27,28,29^ Families having the same household income may experience different neighborhood contexts due to different costs of living and/or structural racism barriers.^6^ Mechanistically, neighborhood SES could reflect environmental (eg, pollution and toxins) and social influences (eg, peer interactions), whereas household SES may reflect home characteristics, such as material access and parenting practices.^6,7,30^ However, it remains unknown how neighborhood and household SES are associated with white matter development and via what mechanisms. Identifying mediating factors within this framework may reveal intervention targets to promote brain health in disadvantaged children.

**Figure 1.**
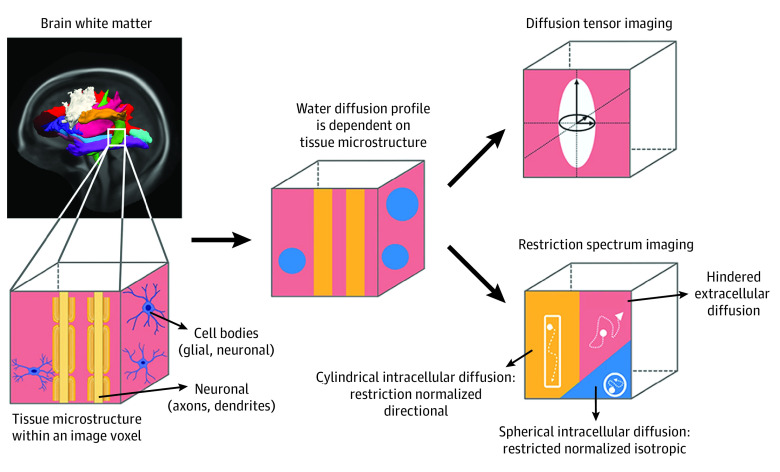
Restriction Spectrum Imaging and Diffusion Tensor Imaging Models Restriction spectrum imaging restricted normal directional and restricted normal isotropic diffusion were the primary white matter microstructure assessments, and diffusion tensor imaging fractional anisotropy and mean diffusivity were estimated as references. Details on restriction spectrum imaging specification are available in eMethods in Supplement 1. This image was adapted from Burnor et al.^33^

In this study, we examined associations between SES and white matter microstructure in 8842 children from the Adolescent Brain Cognitive Development (ABCD) study. We used the restriction spectrum imaging (RSI) technique, which distinguishes restricted (intracellular) from hindered (extracellular) water diffusion, thus modeling different tissue compartments.^31,32^ We focused on RSI restricted normalized directional (RSI-RND) diffusion, which purportedly reflects oriented axons and dendrites, and RSI restricted normalized isotropic (RSI-RNI) diffusion, which purportedly reflects glial and neuronal cell bodies^32,33,34,35^ (Figure 1). First, given that material deprivation has been associated with reduced myelination and elevated microglial activity,^17,36^ we hypothesized that lower SES would be associated with lower RSI-RND and greater RSI-RNI. Second, we hypothesized that lower neighborhood and household SES would be independently associated with white matter microstructural differences, consistent with findings in gray matter. Third, we hypothesized that obesity and cognition would account for covariance in the associations between SES and white matter microstructure, thereby supporting the plausibility of these factors being mediators.

## Methods

### Participants

Participants were from the ABCD study, a 10-year cohort study tracking child brain development across 21 US sites, with school-based recruitment used to represent the US population.^37,38^ Participants receive ongoing annual clinical interviews and biennial neuroimaging and cognitive testing.^32,39,40,41^ We analyzed the ABCD study baseline data (release 4.0), which were collected between October 1, 2016, and October 31, 2018. In addition to the ABCD study exclusions,^37^ we excluded participants with (1) missing age, sex, or anthropometric data; (2) T1 or diffusion-weighted magnetic resonance imaging (MRI) scans that failed quality control or had clinically significant incidental findings^32^; and (3) a history of severe neurological or psychiatric conditions, diabetes, or eating disorders (eMethods in Supplement 1). These criteria identified 8842 of 11 875 total children for inclusion (eFigure 1 and eTable 1 in Supplement 1). Data analysis was conducted from July 11 to December 19, 2022. Institutional review boards at study sites approved the procedures. All parents or caregivers and children provided written informed consent and assent. This study followed the Strengthening the Reporting of Observational Studies in Epidemiology (STROBE) guideline for cross-sectional studies.

### Measures

Descriptions of how study variables correspond to ABCD study instrument names are shown in eTable 2 in Supplement 1.

#### Neighborhood SES

The participant’s primary home address was geocoded into a Census tract, from which the 17 American Community Survey (2011-2015) ADI estimates were extracted.^42^ Following previous studies,^11,25^ we created a factor-analyzed latent neighborhood disadvantage score, which included 10 modern housing market–independent ADI constructs with high loadings (eTable 3 in Supplement 1). Higher neighborhood disadvantage scores suggested lower educational attainment, income, and property ownership and higher unemployment, poverty, income disparity, and percentage of single-parent households, all at the community level.

#### Household SES

Household income was defined as the combined annual income from all family members. Because income was assessed in ordinal ranges, we divided the midpoint of each income bracket by $10 000, creating a continuous variable.^28^ Parental educational attainment was defined as the highest educational level among parents or caregivers, which was recoded into years of schooling estimated per US convention^26,29^ (eMethods in Supplement 1).

#### Neuroimaging

Participant T1-weighted structural scans and multishell diffusion-weighted images (DWIs) were collected following standardized protocols across 3T scanners (Prisma and Prisma Fit [Siemens Healthineers], Discovery MR750 [GE HealthCare], and Achieva dStream and Ingenia [Koninklijke Philips]) at study sites.^41^ As part of the ABCD study’s centralized neuroimaging processing (eMethods in Supplement 1),^32^ RSI was fitted to fiber orientation density functions derived from distortion-corrected DWIs to model RSI-RND and RSI-RNI. To evaluate the convergent validity of our novel RSI-based results, we repeated analyses with diffusion tensor imaging (DTI) fractional anisotropy (DTI-FA) and DTI mean diffusivity (DTI-MD). Previous work suggested a positive association between RSI-RND and DTI-FA because both reflect anisotropic diffusion and myelin organization.^34,43^ Low SES has been consistently associated with lower DTI-FA,^12,13,14,15,16^ but findings have been mixed for DTI-MD. Thus, we expected a similar pattern of results between RSI-RND and DTI-FA.

Major white matter tracts were delineated by matching the prior probabilities and diffusion orientations of tracts in the AtlasTrack template to those in participant DWIs.^32,44^ Measurements for RSI and DTI were extracted from 31 tracts,^44^ including the corpus callosum, with forceps major and forceps minor subregions; bilateral fornix (Fx); bilateral cingulate cingulum; bilateral parahippocampal cingulum (CgH); bilateral corticospinal (or pyramidal) tract (CST); bilateral anterior thalamic radiations (ATRs); bilateral uncinate fasciculus (Unc); bilateral inferior longitudinal fasciculus (ILF); bilateral inferior frontal occipital fasciculus; bilateral superior longitudinal fasciculus (SLF), with bilateral temporal (tSLF; ie, arcuate fasciculus) and bilateral parietal (pSLF) subregions; bilateral superior corticostriatal tract (SCS); bilateral striatal to inferior frontal cortical tract; and bilateral inferior frontal to superior frontal cortical tract. Individual tracts are shown in eFigure 2 in Supplement 1.

#### Obesity-Related Measures

Participant waist circumference, height, and weight were each averaged across up to three measurements. Body mass index (BMI) was calculated as weight in kilograms divided by height in meters squared. Age and sex-corrected BMI *z* scores were computed using the 2000 Centers for Disease Control and Prevention growth charts.^45^ We used different obesity-related measures to account for their varied accuracy in reflecting adiposity in children.^46,47^

#### Cognitive Performance

We used the age-corrected total cognition score from the National Institutes of Health Toolbox Cognition Battery, which reflected performance on executive functioning, memory, language, and processing speed tasks^40^ (eMethods in Supplement 1). Follow-up analyses using individual task scores and crystallized and fluid cognition scores were conducted to explore whether associations were specific to certain cognitive domains.

### Statistical Analysis

Analyses were performed using R software, version 4.2.1 (R Foundation for Statistical Computing). Outliers that were greater than or less than 4 SD away from the mean were removed. Because the missingness of SES variables seemed to pertain to demographic characteristics (eFigure 3 in Supplement 1), we generated 50 imputed data sets using the mice package in R software.^48^ Neuroimaging and cognitive variables, being primary outcomes, were not imputed (eTable 4 in Supplement 1). Imputation did not change data distribution (eTable 5 in Supplement 1). Furthermore, we harmonized RSI and DTI data using the batch-adjustment algorithm ComBat, version 1.0.13,^49^ reducing scanner differences while preserving inherent associations between neuroimaging metrics and demographics (eMethods and eTable 6 in Supplement 1).

#### Associations With SES

Associations between SES and white matter microstructure were assessed using linear mixed-effects models (lme4 package in R software^50^) in which neighborhood disadvantage, household income, and parental educational attainment were independent variables (in the same model), and white matter microstructure was the dependent variable (each RSI or DTI measurement in a separate model). Age, sex, pubertal development stage,^51^ intracranial volume (ICV; estimated from T1-weighted images), and mean head motion (ie, the mean DWI framewise displacement) were covaried due to potential confounding,^34,52,53^ and family was modeled as a random effect. Associations between SES and obesity and cognitive measures were examined similarly, except without covarying for ICV and mean head motion. Because SES was highly confounded with parent- or caregiver-reported participant race and ethnicity (eTable 7 in Supplement 1), we did not adjust for race and ethnicity to preserve SES-related variance.^54^ Covarying for race and ethnicity produced more restricted findings (eTable 11 in Supplement 1). All models were checked for normality of residuals, homoscedasticity, and low multicollinearity (variance inflation factors were ≤1.89). Estimates were standardized β coefficients with 95% CIs pooled across imputed data sets. Multiple comparison correction was performed within each group of models and by each SES indicator using false discovery rate (FDR) at 2-tailed *P* ≤ .05. Because missing neuroimaging and cognition data were not imputed, sample size varied across models (eTable 8 in Supplement 1).

In sensitivity analyses, we assessed associations between SES and white matter microstructure in subsamples that further limited potential confounders, which included (1) participants with mean head motion of 2.5 mm or lower, (2) participants without adverse childhood experiences, (3) participants without common psychiatric diagnoses, and (4) participants with full-term birth (eMethods in Supplement 1).

#### Indirect Associations

We estimated the indirect associations between SES and white matter microstructure through obesity-related measures and cognitive performance using structural equation models (lavaan package in R software^55^). Such analyses, while based on cross-sectional data, can establish the plausibility of factors as potential mediators that could be confirmed in future longitudinal studies. To reduce data dimensionality, principal component (PC) analyses were applied to RSI or DTI measurements in tracts that had significant associations with SES. Structural equation models specified SES factors as independent variables, obesity or cognitive measure as the mediator, and each of the white matter microstructure PCs as the dependent variable. Covariates included age, sex, pubertal development stage, ICV, and mean head motion. Because multilevel modeling was not feasible in the lavaan package, findings were confirmed in randomly selected unrelated participants that eliminated twin or siblingship confounders (eTable 21 in Supplement 1). Extending previous research,^11,24,29^ we tested additional models with white matter microstructure PCs as mediators and cognitive performance as the dependent variable. Standardized estimates were computed with SEs and 95% CIs (from 20 000 Monte Carlo simulations). Statistical significance was set at 2-tailed *P* ≤ .05 corrected for FDR across all tested models.

## Results

Among 8842 children, 4299 (48.6%) were girls, 4543 (51.4%) were boys, and the mean (SD) age was 9.9 (0.7) years (Table). The 3 SES factors (neighborhood disadvantage, household income, and parental educational attainment) had moderate to high correlations with each other (eFigure 4 in Supplement 1).

**Table.** Participant Characteristics

Characteristic	Participants, No. (%)^a^		*P* value^b^
	Current sample (n = 8842)	Full ABCD study (n = 11 875)	
Age, mean (SD), mo	119 (8)	119 (7)	.48
Sex			
Female	4299 (48.6)	5641 (47.5)	.23
Male	4543 (51.4)	6170 (52.0)	
Race and ethnicity			
Asian	183 (2.1)	251 (2.1)	.11
Black	1212 (13.7)	1757 (14.8)	
Hispanic	1805 (20.4)	2404 (20.2)	
White	4738 (53.6)	6153 (51.8)	
Other^c^	902 (10.2)	1244 (10.5)	
PDS			
Prepuberty	4425 (50.0)	5837 (49.2)	.91
Early puberty	2009 (22.7)	2713 (22.8)	
Midpuberty	1966 (22.2)	2673 (22.5)	
Late puberty	124 (1.4)	171 (1.4)	
Postpuberty	7 (0.1)	10 (0.1)	
Neighborhood disadvantage, mean (SD) [range]^d^	0 (8.3) [−14 to 37]	0 (8.2) [−14 to 36]	.99
Household income, mean (SD)^e^	10.0 (6.2)	9.7 (6.2)	<.001
Parental educational attainment, mean (SD), y	15.9 (2.8)	15.8 (2.9)	.002
BMI, mean (SD)	18.6 (4.0)	18.8 (4.2)	.002
Waist circumference, mean (SD), cm	66.8 (10.2)	67.3 (10.9)	.001
BMI *z* score, mean (SD)	0.4 (1.2)	0.4 (1.4)	.30
Obesity status^f^			
Underweight	367 (4.2)	468 (3.9)	.12
Normal weight	5759 (65.1)	7550 (63.6)	
Overweight	1322 (15.0)	1784 (15.0)	
Obesity	1391 (15.7)	1997 (16.8)	
Total cognition score, mean (SD)	101.4 (17.6)	100.4 (18.0)	<.001
Head motion, mean (SD), mm	1.3 (0.4)	1.4 (0.6)	<.001
ICV, mean (SD), mm^3^	1 492 802 (142 204)	1 489 474 (143 907)	.10

Abbreviations: ABCD, Adolescent Brain Cognitive Development; BMI, body mass index (calculated as weight in kilograms divided by height in meters squared); ICV, intracranial volume; PDS, pubertal development stage.

^a^ For some variables, numbers may not sum to the total and percentages may not sum to 100 due to missing data.

^b^ Comparisons were performed using a 2-tailed Pearson χ^2^ test for categorical variables or a *t* test for continuous variables.

^c^ Includes parent- or caregiver-reported American Indian or Alaska Native, Native Hawaiian or other Pacific Islander, multiple races and/or ethnicities, and unknown race and ethnicity.

^d^ Because neighborhood disadvantage was a scaled variable, its minimum and maximum values are also reported for reference.

^e^ Household income was defined as the combined annual income from all family members. Because income was assessed in ordinal ranges, the midpoint of each income bracket was divided by $10 000, creating a continuous variable.

^f^ Obesity status was derived from the participant’s age- and sex-adjusted BMI percentiles, with underweight defined as lower than 5th percentile, normal weight as 5th percentile to lower than 85th percentile, overweight as 85th percentile to lower than 95th percentile, and obesity as 95th percentile or higher.^45^

### Associations Between SES and White Matter Microstructure

Among 31 total tracts, lower SES was associated with lower RSI-RND in 5 tracts (bilateral CST, forceps major, and bilateral SLF [including bilateral tSLF and bilateral pSLF]), greater RSI-RNI in 24 tracts (bilateral Fx, bilateral cingulate cingulum, bilateral CgH, bilateral CST, bilateral ATRs, bilateral Unc, bilateral ILF, bilateral inferior frontal occipital fasciculus, corpus callosum [including forceps major and forceps minor], bilateral SLF [including left tSLF and bilateral pSLF], left SCS, bilateral striatal to inferior frontal cortical tract, and bilateral inferior frontal to superior frontal cortical tract), and lower DTI-FA in 6 tracts (left Fx, bilateral CST, bilateral SLF [including bilateral tSLF and bilateral pSLF], and left SCS) (full statistics are available in eTable 9, and a heat map is available in eFigure 5 in Supplement 1). Findings were generally consistent in the sensitivity analyses (eTable 10 in Supplement 1).

#### Associations With RSI-RND

**Figure 2.**
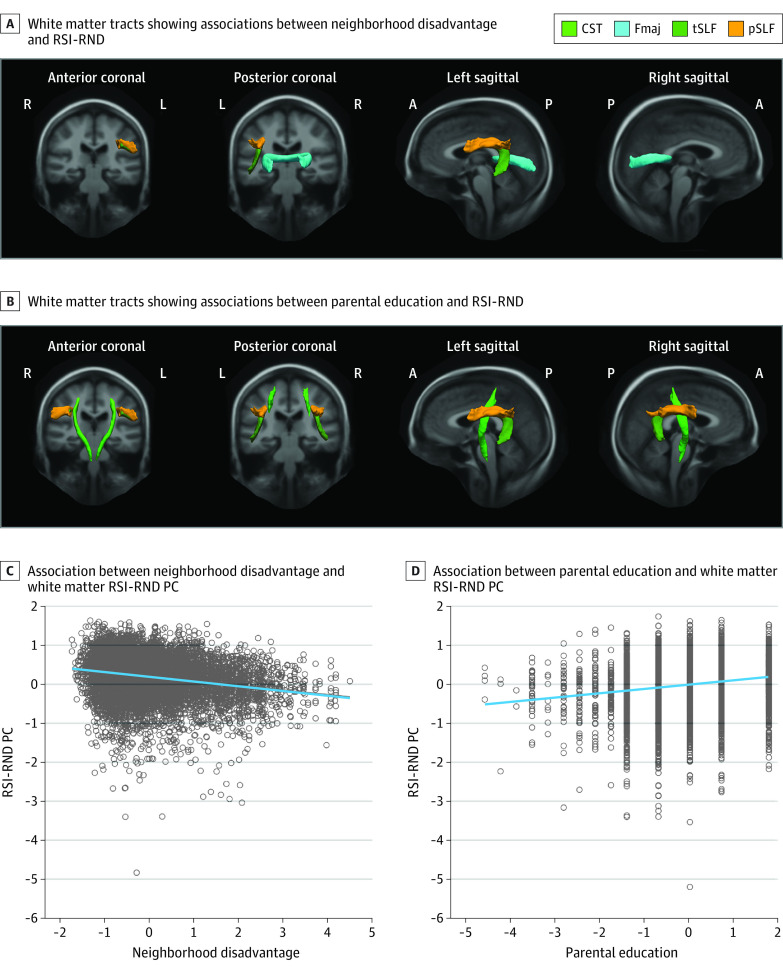
Associations Between Socioeconomic Status and White Matter Restriction Spectrum Imaging (RSI) Restricted Normalized Directional (RND) Diffusion Tracts were visualized using the AtlasTrack template.^32,44^ The principal components (PCs) summarized RSI-RND in the involved tracts. In scatterplots, linear regression lines were adjusted for covariates and flanked by shaded 95% confidence intervals. Data points were standardized residuals extracted from a randomly selected multiply imputed data set (50 total data sets) as reference. Covariates included age, sex, pubertal development stage, intracranial volume, and head motion, with family as the random effect. Detailed statistics are shown in eTable 9 and eTable 13 in Supplement 1. A indicates anterior; CST, corticospinal or pyramidal tract; Fmaj, forceps major; L, left hemisphere; P, posterior; R, right hemisphere; and SLF, superior longitudinal fasciculus, including parietal (pSLF) and temporal (tSLF) subregions.

Higher neighborhood disadvantage was independently associated with lower RSI-RND in the forceps major (β = −0.040; 95% CI, −0.067 to −0.013; *P* = .03 corrected for FDR) and left SLF (β = −0.055; 95% CI, −0.081 to −0.028; *P* = .001 corrected for FDR) (Figure 2; eTable 9 in Supplement 1). Lower parental educational attainment was independently associated with lower RSI-RND in the bilateral CST (right hemisphere: β = 0.042; 95% CI, 0.015-0.069; *P* = .01 corrected for FDR) and bilateral SLF (right hemisphere: β = 0.053; 95% CI, 0.025-0.080; *P* = .002 corrected for FDR). No association was observed for household income (eg, forceps major: β = 0.022 [95% CI, −0.008 to 0.053; *P* = .51 corrected for FDR]; left SLF: β = 0.014 [95% CI, −0.016 to 0.044; *P* = .55 corrected for FDR]).

#### Associations With RSI-RNI

**Figure 3.**
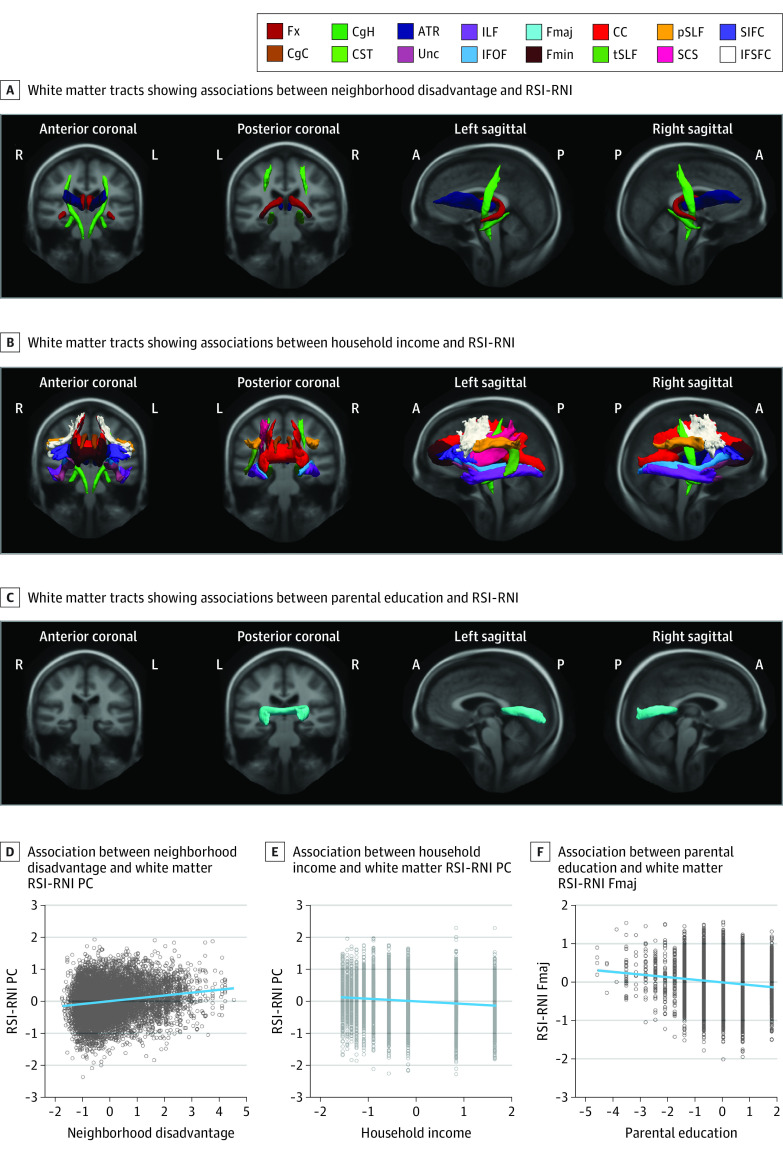
Associations Between Socioeconomic Status and White Matter Restriction Spectrum Imaging (RSI) Restricted Normalized Isotropic (RNI) Diffusion Tracts were visualized using the AtlasTrack template.^32,44^ The principal components (PCs) summarized RSI-RNI in the involved tracts. In scatterplots, linear regression lines were adjusted for covariates and flanked by shaded 95% CIs. Data points were standardized residuals extracted from a randomly selected multiply imputed data set (50 total data sets) as reference. Covariates included age, sex, pubertal development stage, intracranial volume, and head motion, with family as the random effect. Detailed statistics are shown in eTables 9 and 13 in Supplement 1. A indicates anterior; ATR, anterior thalamic radiation; CC, corpus callosum; CgC, cingulate cingulum; CgH, parahippocampal cingulum; CST, corticospinal or pyramidal tract; Fmaj, forceps major; Fmin, forceps minor; Fx, fornix; IFOF, inferior frontal occipital fasciculus; IFSFC, inferior frontal to superior frontal cortical tract; ILF, inferior longitudinal fasciculus; L, left hemisphere; P, posterior; R, right hemisphere; SCS, superior corticostriatal tract; SIFC, striatal to inferior-frontal cortical tract; SLF, superior longitudinal fasciculus, including temporal (tSLF) and parietal (pSLF) subregions; and Unc, uncinate fasciculus.

All 3 SES factors were independently associated with white matter RSI-RNI (Figure 3; eTable 9 in Supplement 1). Lower household income was associated with higher RSI-RNI in almost every tract, including the right ILF (β = −0.042; 95% CI, −0.073 to −0.012; *P* = .01 corrected for FDR) and right ATRs (β = −0.045; 95% CI, −0.075 to −0.014; *P* = .01 corrected for FDR), and except for the bilateral Fx (eg, right hemisphere: β = 0.008; 95% CI, −0.023 to 0.040; *P* = .63 corrected for FDR), forceps major (β = −0.005; 95% CI, −0.036 to 0.025; *P* = .75 corrected for FDR), right tSLF (β = −0.026; 95% CI, −0.056 to 0.004; *P* = .10 corrected for FDR), and right SCS (β = −0.022; 95% CI, −0.052 to 0.008; *P* = .17 corrected for FDR). Higher neighborhood disadvantage was associated with greater RSI-RNI in the bilateral Fx (eg, right hemisphere: β = 0.046; 95% CI, 0.019-0.074; *P* = .01 corrected for FDR) and, overlapping with household income, in the bilateral CgH (eg, right hemisphere: β = 0.061; 95% CI, 0.034-0.088; *P* < .001 corrected for FDR), bilateral CST (eg, right hemisphere: β = 0.037; 95% CI, 0.010-0.065; *P* = .03 corrected for FDR), and bilateral ATRs (eg, right hemisphere: β = 0.045; 95% CI, 0.018-0.072; *P* = .01 corrected for FDR). Lower parental educational attainment was associated with higher RSI-RNI in the forceps major only (β = −0.048; 95% CI, −0.077 to −0.020; *P* = .03 corrected for FDR).

#### Associations With DTI-FA and DTI-MD

Associations of SES with DTI-FA largely resembled those with RSI-RND. Higher neighborhood disadvantage had independent associations with lower DTI-FA in the left SLF (β = −0.066; 95% CI, −0.094 to −0.039; *P* < .001 corrected for FDR), and lower parental educational attainment had independent associations with lower DTI-FA in the left Fx (β = 0.042; 95% CI, 0.013-0.070; *P* = .01 corrected for FDR), bilateral CST (eg, right hemisphere: β = 0.045; 95% CI, 0.018-0.073; *P* = .01 corrected for FDR), bilateral SLF (eg, right hemisphere: β = 0.050; 95% CI, 0.021-0.078; *P* = .005 corrected for FDR), and left SCS (β = 0.039; 95% CI, 0.011-0.067; *P* = .02 corrected for FDR) (eFigure 6 and eTable 9 in Supplement 1). Household income was not associated with DTI-FA (eg, right SLF: β = −0.002; 95% CI, −0.033 to 0.030; *P* = .96 corrected for FDR), and none of the SES factors was associated with DTI-MD (eg, parental educational attainment and right Fx: β = 0.009; 95% CI, −0.019 to 0.038; *P* = .76 corrected for FDR).

### Analyses of Indirect Associations

The first PCs carried substantial loadings from all involved tracts, captured 58% to 95% of variance, and were associated with SES in similar direction and magnitude, as individual tracts did (eTable 12 and eTable 13 in Supplement 1). These PCs were used to represent SES-associated white matter microstructural differences. All structural equation models had good fit (eTable 18 in Supplement 1).

#### Indirect Associations via Obesity-Related Measures

**Figure 4.**
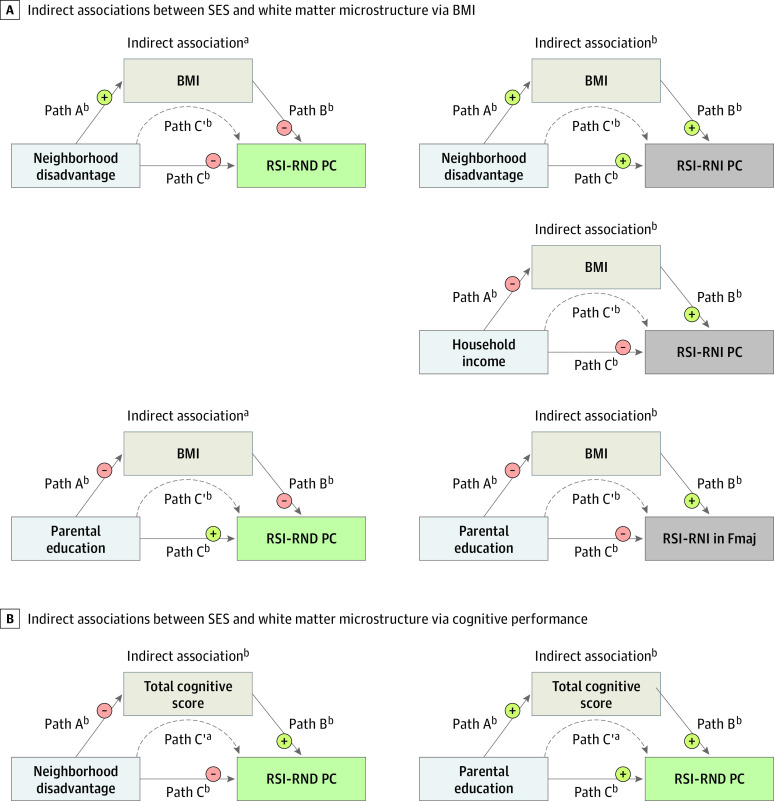
Indirect Associations Between Socioeconomic Status (SES) and White Matter Microstructure via Obesity and Cognition In each structural equation model, covariates included age, sex, pubertal development stage, intracranial volume, and head motion, as well as the SES factors that were not the independent variable in question (eg, household income and parental educational attainment were covariates in models of neighborhood disadvantage). Detailed statistics are available in eTables 15 and 17 in Supplement 1, and model fit indices are available in eTable 18 in Supplement 1. For indirect associations, *P* values were corrected using false discovery rate (FDR). BMI indicates body mass index (calculated as weight in kilograms divided by height in meters squared); Fmaj, forceps major; PC, principal component; RND, restricted normalized directional; RNI, restricted normalized isotropic; and RSI, restriction spectrum imaging. ^a^*P* ≤ .01 corrected for FDR. ^b^*P* ≤ .001 corrected for FDR.

Lower SES indexed by all 3 SES factors had independent associations with higher values for all obesity-related measures (eTable 14 in Supplement 1). Higher BMI partially accounted for covariance in associations between higher neighborhood disadvantage and a lower RSI-RND PC (β = −0.004; 95% CI, −0.006 to −0.001; *P* = .003 corrected for FDR) and a greater RSI-RNI PC (β = 0.015; 95% CI, 0.011-0.020; *P* < .001 corrected for FDR), between lower household income and a greater RSI-RNI PC (β = −0.008; 95% CI, −0.011 to −0.004; *P* < .001 corrected for FDR), and between lower parental educational attainment and a lower RSI-RND PC (β = 0.005; 95% CI, 0.003-0.008; *P* = .007 corrected for FDR) and greater RSI-RNI in the forceps major (β = −0.009; 95% CI, −0.012 to −0.006; *P* < .001 corrected for FDR) (Figure 4A; eTable 15 in Supplement 1). No indirect association was observed with DTI-FA PCs (eg, neighborhood disadvantage: β = 0; 95% CI, −0.003 to 0.002; *P* = .91 corrected for FDR). Results were consistent with waist circumference and age- and sex-adjusted BMI *z* scores (eTable 15 in Supplement 1).

#### Indirect Associations via Cognitive Performance

Lower SES indexed by all 3 SES factors was independently associated with lower total cognition score (eTable 16 in Supplement 1). Lower total cognition score partially accounted for covariance in associations of both higher neighborhood disadvantage and lower parental educational attainment with lower RSI-RND PCs (neighborhood disadvantage: β = −0.012 [95% CI, −0.016 to −0.009; *P* < .001 corrected for FDR]; parental educational attainment: β = 0.019 [95% CI, 0.013-0.025; *P* < .001 corrected for FDR]) and DTI-FA PCs (neighborhood disadvantage: β = −0.010 [95% CI, −0.014 to −0.007; *P* < .001 corrected for FDR]; parental educational attainment: β = 0.016 [95% CI, 0.010-0.022; *P* < .001 corrected for FDR]) (Figure 4B; full statistics in eTable 17 in Supplement 1). The total cognition score was not associated with RSI-RNI PCs; thus, indirect associations were not tested in these models. Results were consistent across most analyses using composite and individual task scores, suggesting that the observed indirect associations pertained to general and not domain-specific cognition (eTable 19 in Supplement 1). In models in which cognition was the dependent variable, we observed indirect associations between lower SES and poorer cognition via lower RSI-RND (eg, neighborhood disadvantage: β = −0.004 [95% CI, −0.007 to −0.002; *P* < .001 corrected for FDR]; parental educational attainment: β = 0.004 [95% CI, 0.002-0.006; *P* < .001 corrected for FDR]) and DTI-FA (eg, neighborhood disadvantage: β = −0.004 [95% CI, −0.007 to −0.002; *P* < .001 corrected for FDR]; parental educational attainment: β = 0.003 [95% CI, 0.002-0.005; *P* = .001 corrected for FDR]) PCs, also broadly across cognitive domains (eTable 20 in Supplement 1).

## Discussion

In this cross-sectional study of 8842 children aged 9 to 11 years, we found that greater neighborhood disadvantage and lower household SES were independently associated with lower RSI-RND, greater RSI-RNI, and lower DTI-FA in partially overlapping white matter regions. Furthermore, there was evidence that greater BMI and related anthropometric features as well as poorer cognitive performance plausibly mediated the association between lower SES and white matter microstructural differences. Given the large and diverse sample, these results suggested that both neighborhood and household SES might be important for children’s white matter development. Future research may investigate obesity and cognition as possible mechanistic mediators.

Lower SES was associated with lower RSI-RND in the SLF, forceps major, and CST. Because RSI-RND models anisotropic intracellular water diffusion,^31^ these findings might reflect more oriented axonal or dendritic organization in these tracts.^34,35^ Matching results were seen with DTI-FA in our study and previous reports of lower DTI-FA in the SLF^13,15,56,57^ and CST^15,57^ in children from families with lower SES. Lower integrity of these tracts has been associated with worse cognition. The SLF connects the frontal, temporal, and parietal cortices and is associated with language abilities, social cognition, and attention.^57,58^ The forceps major connects bilateral occipital lobes, facilitating interhemispheric visual information transfer critical for visuospatial processing.^59^ The CST, primarily a motor pathway, fine tunes somatosensory-motor integration underlying fast processing speed.^60^ Accordingly, we found that lower cognition scores partially explained the associations between lower SES and reduced integrity in these tracts. These associations might be rationalized by the fact that the amount of cognitive stimulation a child receives is somewhat dependent on SES,^30,61,62^ although our cross-sectional data precluded inference of directionality. In fact, when white matter microstructure was used as the mediator, results also supported the possibility that SES may be first associated with neurodevelopmental outcomes, which then play a role in cognition.^11,24^ Cognitive performance likely has both sociocultural and neurobiological foundations; as such, it could be conceptualized as either an underlying factor in or manifestation of brain development,^23,30,62^ and future research may dissociate these using more specific measurements. In addition, because cortical regions connected to the white matter tracts identified here have also been associated with SES and cognition in children,^10,11,23,24,27,28,29^ our findings might be part of a larger neurodevelopmental pattern rather than an isolated process.^63^

Contrasting the localized findings seen with RSI-RND, lower SES was associated with greater RSI-RNI in almost every white matter tract, suggesting that SES might modulate glial and/or neuronal cell presence in a brainwide fashion. Our observation that greater BMI and related anthropometric features partially accounted for these associations proposes some potential mechanisms. First, obesity induces systemic inflammation, which upregulates circulating proinflammatory molecules^64^ that can infiltrate brain tissue through a weakened blood-brain barrier.^65,66^ The brain’s astrocytes and microglia undergo reactive gliosis, an immune response marked by their proliferation and enlargement as seen in rodent models of obesity.^66,67,68,69^ In diffusion MRI, this neuroinflammatory phenotype could manifest as increases in isotropic intracellular diffusion, a phenomenon observed in the striatum,^70,71,72,73^ hypothalamus,^73^ and widespread white matter tracts^73^ in human obesity. Thus, it is possible that low SES might be associated with neuroinflammation via obesity.^3^ Also, microglia dysregulation may cause myelin damage,^74^ which could explain our observed association between obesity and RSI-RND. Second, because RSI-RNI increases across typical development,^34^ our findings of greater RSI-RNI after covarying for age may reflect accelerated white matter maturation in those with socioeconomic disadvantage. This interpretation is consistent with reports of higher gray matter–derived brain age among adolescents with low SES^75,76,77^ and parallels the stress acceleration hypothesis, which proposes prioritized neuroadaptation to adversity.^78^ The associations between SES and the largest differences in RSI-RNI were seen in frontolimbic pathways (Fx, CgH, ATRs, and Unc) relevant to emotion processing, stress, and depression.^79,80,81^ Notably, obesity has been associated with earlier pubertal onset^82^ and advanced brain aging^83,84^ and thus might embody a way in which SES impacts brain development. Future animal research comparing RSI with cellular or tissue imaging may help elucidate the exact neurobiological basis underlying our observed SES-associated white matter microstructural differences.

The role of obesity and cognitive performance in the association of SES with white matter development, if confirmed in longitudinal analyses, could motivate future work to examine how to promote brain health in children with low SES. Weight loss, low-fat dieting, and aerobic training have been found to increase cortical volumes and white matter density^85,86,87,88^ and attenuate neuroinflammation.^89^ Environments rich in social and sensory stimuli are known to promote myelination in hypoxic rodents^61^ and aging adults.^90^ Because both neighborhood and household SES were independently associated with white matter microstructure, interventions may need to consider multiple socioeconomic perspectives. Improved neighborhood access to healthy food outlets and playgrounds may limit obesity among children living in low-SES areas.^91,92^ Household factors, such as buying power and fitness awareness, are also important for maintaining healthy weight.^92,93^ For cognition, school sports and music programs have been noted to improve children’s academic achievement and cognitive performance.^94,95^ At home, enriching parent-child interactions, such as reading and play, benefit children’s cognitive and overall well-being.^96^ Many of these potential mediating mechanisms are filtered through structural forces, such as occupation, economic circumstances (eg, job market and housing market), and allocation of government resources; thus, their management should be considered in terms of social policy. Further research is warranted to characterize the specific consequences of these factors together with other unexplored factors, such as crime and pollution,^6,7,33^ for brain development.

### Limitations

This study has several limitations, including its cross-sectional design, which precludes causal inference and does not address whether findings are temporary or long lasting. Longitudinal investigations will help assess how white matter microstructural differences evolve over time and are associated with health outcomes. In addition, SES factors, especially educational attainment, have genetic backgrounds^97,98^; research is needed to dissociate environmental from genetic roles in white matter development. In addition, SES factors were not estimators of brain structure at the individual level; findings were seen only in a large population-based sample. Future studies are also warranted to examine other facets of SES as well as potential interactions.^27^ We only studied major white matter tracts due to the higher reliability of RSI in these regions; future work is needed to assess whether our findings extend to superficial or pericortical white matter.

## Conclusions

In this large cross-sectional study, neighborhood and household socioeconomic adversity were independently associated with white matter microstructural differences in children. These associations were partially explained by obesity and cognition. Future studies could explore interventions from neighborhood and household perspectives to assess whether these factors improve white matter health in disadvantaged youth. Our findings join previous research on gray matter^10,11,23,24,25,26,27,28^ to highlight the complex pathways through which SES might play a role in brain development.

## Notes

Supplement 1. **eMethods.** Inclusion and Exclusion Criteria of the ABCD Study and the Current Study, Derivation of Household SES Factors, Neuroimaging Methods, Cognitive Assessments, Data Harmonization, and Sensitivity Analyses **eFigure 1.** Participant Selection Flowchart **eTable 1.** Characteristics of Excluded ABCD Study Participants **eTable 2.** Lookup List for Relevant ABCD Study Instrument Names **eTable 3.** Factor Loadings of ADI Constructs **eFigure 2.** Isosurface Renderings of White Matter Tracts **eFigure 3.** Relationships Between Missingness of Key Variables **eTable 4.** Missing Data and Roles of Variables in Imputation **eTable 5.** Distribution of Imputed Variables Before and After Imputation of Missing Data **eTable 6.** Associations Between Preserved Variables and Pre- and Postharmonization RSI and DTI Measurements **eTable 7.** Associations Between SES and Race and Ethnicity **eTable 8.** Sample Sizes for Models Testing Associations Between SES, RSI and DTI Measurements, and Cognitive Performance **eFigure 4.** Correlations Between SES Factors **eTable 9.** Associations Between SES and White Matter Microstructure **eFigure 5.** Heat Map of Associations Between SES and White Matter Microstructure **eTable 10.** Sensitivity Analyses on Associations Between SES and White Matter Microstructure **eFigure 6.** Associations Between SES and White Matter DTI FA **eTable 11.** Associations Between SES and White Matter Microstructure Adjusted for Race and Ethnicity **eTable 12.** PCA Loadings of RSI and DTI Measurements That Were Significantly Associated With SES **eTable 13.** Associations Between SES and White Matter Microstructure PCs **eTable 14.** Associations Between SES and Obesity-Related Measures **eTable 15.** Indirect Associations Between SES and White Matter Microstructure via Obesity-Related Measures **eTable 16.** Associations Between SES and Cognitive Performance **eTable 17.** Indirect Associations Between SES and White Matter Microstructure via Total Cognition Scores **eTable 18.** Fit Indices for Main Structural Equation Models **eTable 19.** Indirect Associations Between SES and White Matter Microstructure via Cognitive Task Scores **eTable 20.** Indirect Associations Between SES and Cognitive Performance via White Matter Microstructure **eTable 21.** Indirect Associations in Random Subsample With 1 Participant per Family
eReferences

Supplement 2.
Data Sharing Statement
