# Supplementary material for: Associations Between Socioeconomic Status, Obesity, Cognition, and White Matter Microstructure in Children
Source: JAMA Netw Open. 2023 Jun 27;6(6):e2320276. doi: 10.1001/jamanetworkopen.2023.20276 (PMC10300724; doi:10.1001/jamanetworkopen.2023.20276)
Supplement: Supplement 1. — eMethods. Inclusion and Exclusion Criteria of the ABCD Study and the Current Study, Derivation of Household SES Factors, Neuroimaging Methods, Cognitive Assessments, Data Harmonization, and Sensitivity Analyses eFigure 1. Participant Selection Flowchart eTable 1. Characteristics of Excluded ABCD Study Participants eTable 2. Lookup List for Relevant ABCD Study Instrument Names eTable 3. Factor Loadings of ADI Constructs eFigure 2. Isosurface Renderings of White Matter Tracts eFigure 3. Relationships Between Missingness of Key Variables eTable 4. Missing Data and Roles of Variables in Imputation eTable 5. Distribution of Imputed Variables Before and After Imputation of Missing Data eTable 6. Associations Between Preserved Variables and Pre- and Postharmonization RSI and DTI Measurements eTable 7. Associations Between SES and Race and Ethnicity eTable 8. Sample Sizes for Models Testing Associations Between SES, RSI and DTI Measurements, and Cognitive Performance eFigure 4. Correlations Between SES Factors eTable 9. Associations Between SES and White Matter Microstructure eFigure 5. Heat Map of Associations Between SES and White Matter Microstructure eTable 10. Sensitivity Analyses on Associations Between SES and White Matter Microstructure eFigure 6. Associations Between SES and White Matter DTI FA eTable 11. Associations Between SES and White Matter Microstructure Adjusted for Race and Ethnicity eTable 12. PCA Loadings of RSI and DTI Measurements That Were Significantly Associated With SES eTable 13. Associations Between SES and White Matter Microstructure PCs eTable 14. Associations Between SES and Obesity-Related Measures eTable 15. Indirect Associations Between SES and White Matter Microstructure via Obesity-Related Measures eTable 16. Associations Between SES and Cognitive Performance eTable 17. Indirect Associations Between SES and White Matter Microstructure via Total Cognition Scores eTable 18. Fit Indices for Main Structural Equation Models eTable 19. Indirect [file jamanetwopen-e2320276-s001.pdf]

Supplementary Online Content

Li ZA, Cai Y, Taylor RL, et al. Associations between socioeconomic status, obesity, cognition, and white matter microstructure in children. *JAMA Netw Open*. 2023;6(6):e2320276. doi:10.1001/jamanetworkopen.2023.20276

eMethods. Inclusion and Exclusion Criteria of the ABCD Study and the Current Study, Derivation of Household SES Factors, Neuroimaging Methods, Cognitive Assessments, Data Harmonization, and Sensitivity Analyses

eFigure 1. Participant Selection Flowchart

eTable 1. Characteristics of Excluded ABCD Study Participants

eTable 2. Lookup List for Relevant ABCD Study Instrument Names

eTable 3. Factor Loadings of ADI Constructs

eFigure 2. Isosurface Renderings of White Matter Tracts

eFigure 3. Relationships Between Missingness of Key Variables

eTable 4. Missing Data and Roles of Variables in Imputation

eTable 5. Distribution of Imputed Variables Before and After Imputation of Missing Data

eTable 6. Associations Between Preserved Variables and Pre- and Postharmonization RSI and DTI Measurements

eTable 7. Associations Between SES and Race and Ethnicity

eTable 8. Sample Sizes for Models Testing Associations Between SES, RSI and DTI Measurements, and Cognitive Performance

eFigure 4. Correlations Between SES Factors

eTable 9. Associations Between SES and White Matter Microstructure

eFigure 5. Heat Map of Associations Between SES and White Matter Microstructure

eTable 10. Sensitivity Analyses on Associations Between SES and White Matter Microstructure

eFigure 6. Associations Between SES and White Matter DTI FA

eTable 11. Associations Between SES and White Matter Microstructure Adjusted for Race and Ethnicity

eTable 12. PCA Loadings of RSI and DTI Measurements That Were Significantly Associated With SES

eTable 13. Associations Between SES and White Matter Microstructure PCs

eTable 14. Associations Between SES and Obesity-Related Measures

eTable 15. Indirect Associations Between SES and White Matter Microstructure via Obesity-Related Measures

eTable 16. Associations Between SES and Cognitive Performance

eTable 17. Indirect Associations Between SES and White Matter Microstructure via Total Cognition Scores

eTable 18. Fit Indices for Main Structural Equation Models

eTable 19. Indirect Associations Between SES and White Matter Microstructure via Cognitive Task Scores

eTable 20. Indirect Associations Between SES and Cognitive Performance via White Matter Microstructure

eTable 21. Indirect Associations in Random Subsample With 1 Participant per Family

eReferences

This supplemental material has been provided by the authors to give readers additional information about their work.

© 2023 Li ZA et al. *JAMA Network Open*.

eMethods. Inclusion and Exclusion Criteria of the ABCD Study and the Current Study, Derivation of Household SES Factors, Neuroimaging Methods, Cognitive Assessments, Data Harmonization, and Sensitivity Analyses

Inclusion/exclusion criteria of the ABCD Study and the current study. Details on participant recruitment in the Adolescent Brain Cognitive Development (ABCD) Study have been published elsewhere[1]. The ABCD Study was conducted at 21 sites distributed across the U.S., and children were enrolled using a school-based recruitment system. The recruitment catchment of these sites had demographics that reflected the U.S. national, and together encompassed over 20% of U.S. children of the same age. The ABCD Study included children who were between 9 to 11 years of age at baseline, fluent in English, and able to complete the baseline assessments. In the current study, we excluded participants who had 1) missing age, sex, weight, height, or waist circumference data; 2) T1 or diffusion-weighted magnetic resonance imaging (MRI) scans that failed quality control (i.e., scans were required to satisfy visual inspection, FreeSurfer quality control, low head motion, and availability of complete unwarp b0 data)[2] or had clinically significant incidental findings[3]; and 3) history of severe neurological conditions (including cerebral palsy, brain tumor, stroke, aneurysm, brain hemorrhage, hematoma, epilepsy, seizures, intellectual disability, traumatic brain injury, lead poisoning, muscular dystrophy, and multiple sclerosis) or psychiatric conditions (including schizophrenia, autism spectrum disorder, and substance use disorder). We also excluded participants with history of diabetes (the ABCD Study did not specify whether type 1 or 2) or eating disorders (including binge eating disorder, bulimia, and anorexia nervosa), as these conditions may be comorbidities of obesity and confound neurobiological findings[4,5].

Derivation of household income. The ABCD Study defined household income as the total combined income from all household members in the past 12 months, including pre-tax income, wages, rent, social security, benefits, compensation, help from others, etc. Household income was reported in income brackets at non-regular intervals: Less than $5,000; $5,000 through $11,999; $12,000 through $15,999; $16,000 through $24,999; $25,000 through $34,999; $35,000 through $49,999; $50,000 through $74,999; $75,000 through $99,999; $100,000 through $199,999; and $200,000 and greater. Because our indirect association analyses required all variables of interest to be continuous, we followed the procedure in Hackman et al. (2021) that divided the midpoint of each income bracket by $10,000 and scaled the results[6]. The household income midpoints after division were: 0.25, 0.85, 1.4, 2.05, 3, 4.25, 6.25, 8.75, 15, and 20.

Derivation of parental education. The parental education levels reported by the ABCD Study were converted into estimated years of schooling per U.S. convention, as previously used by Rakesh et al. (2021)[7]. This procedure was meant to meet the requirement that variables must be continuous in indirect association analyses.

Education level	Estimated years	Education level	Estimated years	Education level	Estimated years
**Never attended/ Kindergarten only**	0	**8th grade**	8	**Associate degree: Occupational**	14
**1st grade**	1	**9th grade**	9	**Associate degree: Academic Program**	14
**2nd grade**	2	**10th grade**	10	**Bachelor's degree**	16
**3rd grade**	3	**11th grade**	11	**Master's degree**	18
**4th grade**	4	**12th grade**	12	**Professional School degree (ex. MD)**	20
**5th grade**	5	**High school graduate**	12	**Doctoral degree (ex. PhD)**	22
**6th grade**	6	**GED or equivalent Diploma**	12		
**7th grade**	7	**Some college**	14		

MRI sequence specification[2]**.** T1-weighted anatomical images were collected as a 3D T1-weighted inversion prepared RF-spoiled gradient echo scan, with voxel resolution of 1 mm^3 isotropic. Diffusion-weighed images (DWIs) were collected using a multi-shell, multiband echo-planar imaging sequence (acquisition time = 7:31, repetition time = 4100 ms, echo time = 88 ms, matrix size = 140 × 140 × 81, flip angle = 90°, acceleration factor = 3, and voxel resolution = 1.7 mm^3 isotropic). DWIs were imaged with 7 b = 0 frames and 96 gradient directions (b's = 500, 1000, 2000, 3000 s/mm^2 with 6, 15, 15, and 60 directions, respectively).

Restriction spectrum imaging (RSI) model. As part of the centralized neuroimaging processing and analyses performed by the ABCD Study Data Analysis, Informatics, and Research Center[2,8], the RSI model was fitted to DWIs. Within each voxel, the RSI model estimated the relative contributions of water diffusion compartments to the diffusion signal. The compartment of interest in the current study corresponded to restricted diffusion, which could be characterized by the non-Gaussian displacement of water on a typical diffusion length scale < 10 μm; this property was thought reflect the restricted water diffusion profile within cell membrane[9]. The fact that the diffusion signal at different b values was differentially sensitive to different diffusion compartments required multi-shell DWIs, which the ABCD Study acquired[2,9]. RSI modeled the restricted compartment using a fourth-order spherical harmonic function with fixed axial diffusivity of 1×10^{-3} mm^2/s and radial diffusivity of 0 mm^2/s[9]. The restricted normalized isotropic (RNI) metric, thought to reflect water diffusion within spherical neural structures such glia and soma of neurons, was the 0th-order coefficient of the spherical harmonic function of the restricted compartment[9,10]. The restricted normalized directional (RND) metric, thought to reflect anisotropic water diffusion within cylindrical neural structures such as axons and dendrites, was the 2nd-order order coefficient of the spherical harmonic function of the restricted compartment[9,10]. Both RSI-RNI and RND were normalized by all diffusion compartments; as such, they represented the relative contributions of restricted isotropic and directional diffusion to the overall diffusion signal.

Cognitive assessments. Cognitive performance was assessed using the NIH Toolbox Cognition Battery. This suite of seven tests included: flanker inhibitory control test (assessing cognitive control, attention), list sorting working memory test (assessing working memory), dimensional change card sort (assessing executive functioning), oral reading recognition test (assessing reading and language ability), pattern

© 2023 Li ZA et al. *JAMA Network Open*.

comparison test (assessing processing speed), picture sequencing test (assessing episodic memory), and picture vocabulary test (assessing verbal ability). The crystallized cognition score is a composite of the oral reading recognition and picture vocabulary tests, and assesses learned experience. The fluid cognition score is a composite of the flanker inhibitory control, list sorting working memory, dimension change card sort, pattern comparison, and picture sequencing tests, and assesses ability for new learning and information processing[11,12]. The total cognition score is a composite of all seven individual tests. Age-corrected scores were used in the current analyses.

Data harmonization. Technical differences between scanners accounted for substantial variance in RSI and DTI metrics (R^2's = 0.06 to 0.55, p's < .001) and could contaminate statistical inference[13,14]. Because socioeconomic status (SES) variables were unevenly distributed across scanner sites (R^2's ≥ 0.09, p's < .001), the conventional approach of analyzing multi-site ABCD Study data, i.e., including scanner as a random effect in linear mixed-effects models and thus analyzing the effect of each scanner separately, could mean that some scanners only perform analyses over a limited, biased range of SES levels. Thus, this approach may underestimate meaningful SES-related variance. Instead, we harmonized RSI and DTI data using the batch-adjustment algorithm ComBat[14]. ComBat implements an empirical Bayes framework to estimate the additive and multiplicative site effects in a location and scale adjustment model[14]. In our analyses, we implemented the ComBat scripts (https://github.com/Jfortin1/neuroCombat_Rpackage) in R software. Because multiple imputation of missing values took place before this step, we ran ComBat for each of the 50 imputed datasets. All RSI and DTI metrics were the data to be harmonized, scanner was the batch effect to be corrected, and participant age, sex, race/ethnicity, pubertal development stage (PDS), neighborhood disadvantage, household income, and parental education were covariates such that their associations with RSI and DTI metrics would not be incorrectly identified as scanner-related variance and adjusted. ComBat substantially reduced scanner effects (post-harmonization R^2's ≤ 0.002 (> 30-fold reduction compared to pre-harmonization), p's = .007 to > .99). In comparison, associations between RSI and DTI metrics and covariates were mostly preserved, as can be seen in **eTable 6** in the **Supplement**.

Sensitivity analyses. To assess the robustness of our observed associations between socioeconomic status and white matter microstructure, we conducted sensitivity analyses that excluded data from participants with potential neuroimaging confounds. Linear mixed-effects models testing associations between SES and white matter microstructure were re-run within 1) participants with mean head motion during diffusion-weighted MRI scans ≤ 2.5 mm; 2) participants without adverse childhood experiences, defined as having zero parent/caregiver-reported traumatic experience outlined in the Kiddie-Structured Assessment for Affective Disorders and Schizophrenia (K-SADS) for DSM-5, which included experiences of accident, disaster, war, terrorism, community violence, physical abuse, emotional abuse, sexual abuse, witnessing domestic violence, traumatic grief, and bullying[15,16]; 3) participants without common psychiatric diagnoses, including attention-deficit/hyperactivity disorder, depression, bipolar disorder, anxiety, and phobias; and 4) participants with full-term birth.

© 2023 Li ZA et al. *JAMA Network Open*.

eFigure 1. Participant Selection Flowchart

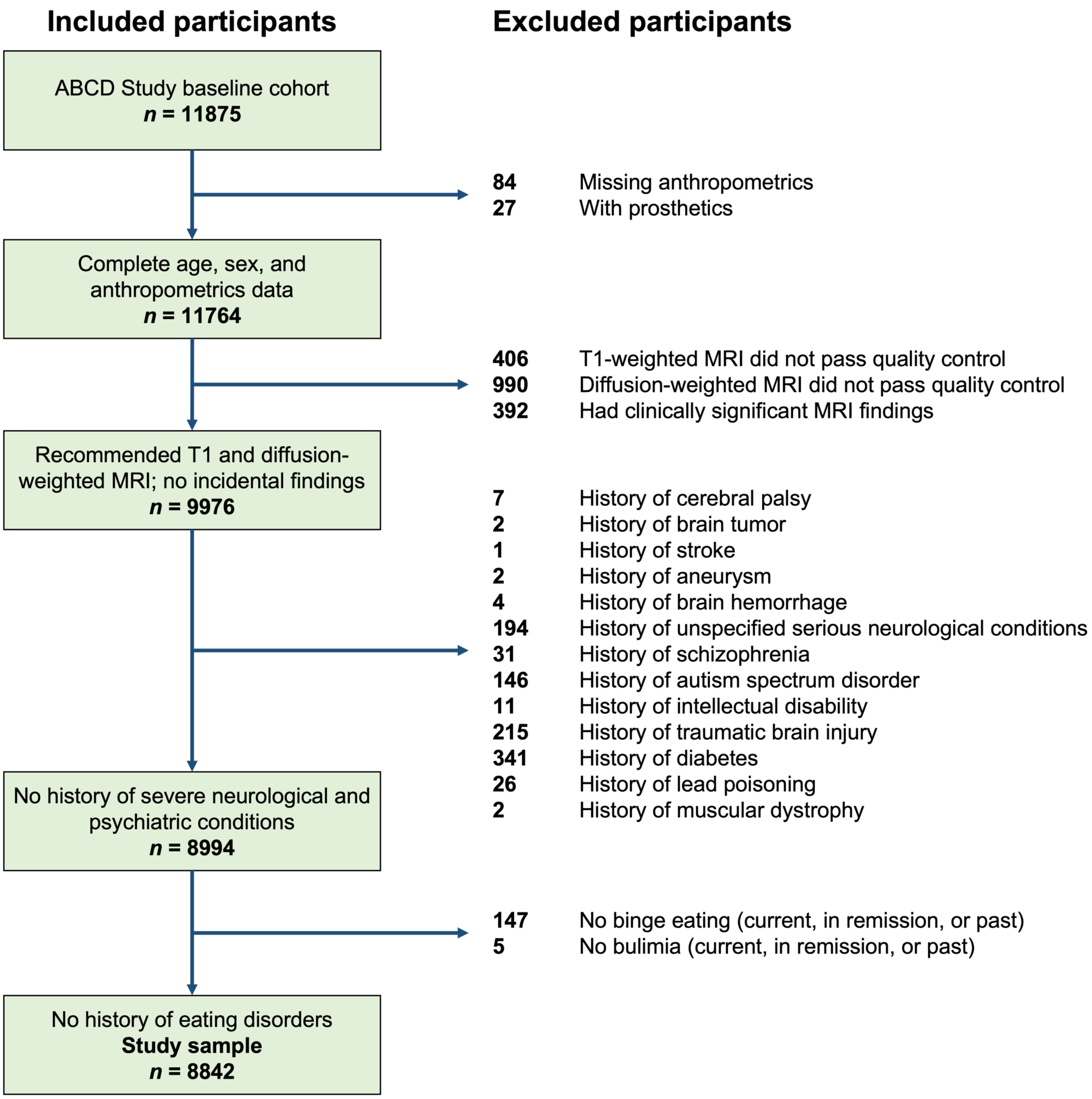

Note. Items that were screened (e.g., history of anorexia nervosa) but had no participant for exclusion are omitted. ABCD, Adolescent Brain Cognitive Development; MRI, magnetic resonance imaging

© 2023 Li ZA et al. *JAMA Network Open*.

eTable 1. Characteristics of Excluded ABCD Study Participants[a]

Measure	Current sample, No. (%)	Excluded participants, No. (%)	*p*-value[b]
**Total No. of participants**	8842	3033	NA
Demographic variables			
**Age, mean (SD), mo**	119 (8)	119 (7)	.06
**Sex**			
Female	4299 (48.6%)	1342 (44.3%)	.001
Male	4543 (51.4%)	1627 (53.6%)	
**Race/ethnicity**			
Asian	183 (2.1%)	68 (2.2%)	< .001
Black	1212 (13.7%)	545 (18.0%)	
Hispanic	1805 (20.4%)	599 (19.7%)	
White	4738 (53.6%)	1415 (46.7%)	
Other[c]	902 (10.2%)	342 (11.3%)	
**PDS**			
Pre-puberty	4425 (50.0%)	1394 (46.0%)	< .001
Early puberty	2009 (22.7%)	686 (22.6%)	
Mid-puberty	1966 (22.2%)	687 (22.7%)	
Late puberty	124 (1.4%)	43 (1.4%)	
Post-puberty	7 (0.1%)	3 (0.1%)	
SES indicators			
**Neighborhood disadvantage, mean (SD) [range]**[d]	0 (8.3) [-14 – 37]	0.9 (8.7) [-13 – 36]	< .001
**Household income, mean (SD)**	10.0 (6.2)	8.8 (6.3)	< .001
**Parental education, mean (SD), y**	15.9 (2.8)	15.5 (2.9)	< .001
Obesity-related measures			
**BMI, mean (SD), kg/m^2**	18.6 (4.0)	19.3 (4.7)	< .001
**Waist circumference, mean (SD), in**	26.3 (4.0)	26.9 (4.7)	< .001
**BMI *z*-score, mean (SD)**	0.4 (1.2)	0.6 (1.2)	< .001
**Obesity status**[e]			
Underweight	367 (4.2%)	100 (3.3%)	< .001
Normal weight	5759 (65.1%)	1791 (59.1%)	
Overweight	1322 (15.0%)	462 (15.2%)	
Obesity	1391 (15.7%)	605 (20.0%)	
Cognitive performance			
**Total cognition score, mean (SD)**	101.4 (17.6)	97.4 (18.2)	< .001
Neuroimaging covariates			
**Mean head motion, mean (SD), mm**	1.3 (0.4)	1.6 (0.9)	< .001
**ICV, mean (SD), mm^3**	1492802 (142204)	1479391 (148517)	< .001

Abbreviations: ABCD, Adolescent Brain Cognitive Development; BMI, body mass index; ICV, intracranial volume; NA, not applicable; PDS, pubertal development stage; SD, standard deviation; SES, socioeconomic status.

[a] Numbers for some variables may not sum up to the total due to missing data.

[b] Comparisons were performed using two-tailed Pearson's χ^2 test (categorical variables) or Student's *t* test (continuous variables).

[c] The "Other" race/ethnicity category included participants who had parent/caregiver-reported American Indian, Alaskan Native, Native Hawaiian, other Pacific Islander, mixed, and otherwise not listed race/ethnicity.

[d] Because neighborhood disadvantage was a scaled variable, its minimum and maximum are also reported for reference.

[e] Obesity status, shown for context, was derived from the participant's age and sex-adjusted BMI percentiles: underweight (BMI < 5th percentile), normal weight (5th to < 85th percentile), overweight (85th to < 95th percentile), and obesity (≥ 95th percentile)[17].

© 2023 Li ZA et al. *JAMA Network Open*.

eTable 2. Lookup List for Relevant ABCD Study Instrument Names[a]

Variable	Data structure (instrument)	Data element (item)
Variables included in statistical analyses		
**Age**	ABCD Youth Anthropometrics (Modified from PhenX)	interview_age
**Sex**	ABCD Youth Anthropometrics (Modified from PhenX)	sex
**Obesity-related measures**	ABCD Youth Anthropometrics (Modified from PhenX)	anthroheightcalc, anthroweight1lb, anthroweight2lb, anthro_waist_cm, anthroweightcast
**Family ID**	ABCD ACS Post Stratification Weights	rel_family_id
**Race/ethnicity**	ABCD ACS Post Stratification Weights	race_ethnicity
**Pubertal development scale (PDS)**	ABCD Sum Scores Physical Health Parent	pds_p_ss_female_category_2, pds_p_ss_male_category_2
**Intracranial volume (ICV)**	ABCD sMRI Part 1	smri_vol_scs_intracranialv
**Neighborhood disadvantage**	Residential History Derived Scores	reshist_addr1_valid, reshist_addr1_adi_edu_l, reshist_addr1_adi_edu_h, reshist_addr1_adi_work_c, reshist_addr1_adi_income, reshist_addr1_adi_in_dis, reshist_addr1_adi_home_v, reshist_addr1_adi_rent, reshist_addr1_adi_mortg, reshist_addr1_adi_home_o, reshist_addr1_adi_crowd, reshist_addr1_adi_unemp, reshist_addr1_adi_pov, reshist_addr1_adi_b138, reshist_addr1_adi_sp, reshist_addr1_adi_ncar, reshist_addr1_adi_ntel, reshist_addr1_adi_nplumb
**Household income**	ABCD Parent Demographics Survey	demo_comb_income_v2
**Parental education**	ABCD Parent Demographics Survey	demo_prnt_ed_v2, demo_prtnr_ed_v2
**Mean head motion**	ABCD dMRI RSI Part 1	dmri_rsi_meanmotion
**White matter RSI-RNI**	ABCD dMRI RSI Part 1	All that starts with dmri_rsirni_fib_...
**White matter RSI-RND**	ABCD dMRI RSI Part 2	All that starts with dmri_rsirnd_fib_...
**White matter DTI-FA**	ABCD dMRI DTI Part 1	All that starts with dmri_dtifa_fiberat_...
**White matter DTI-MD**	ABCD dMRI DTI Part 1	All that starts with dmri_dtimd_fiberat_...
**NIH Toolbox cognition**	ABCD Youth NIH TB Summary Scores	nihtbx_picvocab_agecorrected, nihtbx_flanker_agecorrected, nihtbx_list_agecorrected, nihtbx_cardsort_agecorrected, nihtbx_pattern_agecorrected, nihtbx_picture_agecorrected, nihtbx_reading_agecorrected, nihtbx_fluidcomp_agecorrected, nihtbx_cryst_agecorrected, nihtbx_totalcomp_agecorrected
**Scanner ID**	ABCD MRI Info	mri_info_deviceserialnumber
**Diagnosis of common psychiatric conditions**	ABCD Screener	scrn_commondx
**Adverse childhood experience (ACE)**	ABCD Parent Diagnostic Interview for DSM-5 (KSADS) Traumatic Events	ksads_ptsd_raw_754_p, ksads_ptsd_raw_755_p, ksads_ptsd_raw_756_p, ksads_ptsd_raw_757_p, ksads_ptsd_raw_758_p, ksads_ptsd_raw_759_p, ksads_ptsd_raw_760_p, ksads_ptsd_raw_761_p, ksads_ptsd_raw_762_p, ksads_ptsd_raw_763_p, ksads_ptsd_raw_764_p, ksads_ptsd_raw_765_p, ksads_ptsd_raw_766_p, ksads_ptsd_raw_767_p, ksads_ptsd_raw_768_p, ksads_ptsd_raw_769_p, ksads_ptsd_raw_770_p
**Preterm birth**	ABCD Developmental History Questionnaire	devhx_12a_p
Variables as part of inclusion/exclusion criteria		
**MRI quality control**	ABCD Recommended Imaging Inclusion	imgincl_t1w_include, imgincl_dmri_include
**MRI incidental findings**	ABCD MR Findings	mrif_score
**History of severe neurological conditions**	ABCD Screener	scrn_cpalsy, scrn_tumor, scrn_stroke, scrn_aneurysm, scrn_hemorrhage, scrn_hemotoma, scrn_medcond_other, scrn_epls, scrn_seizure, scrn_con_excl, scrn_intdisab, scrn_tbi_loc, scrn_tbi_mem, scrn_tbi_scan
**History of severe neurological conditions**	ABCD Parent Medical History Questionnaire	medhx_2k, medhx_2l, medhx_2m
**History of severe psychiatric conditions**	ABCD Screener	scrn_schiz, scrn_asd, scrn_sud
**History of obesity comorbidities**	ABCD Parent Medical History Questionnaire	medhx_2g
**History of obesity comorbidities**	ABCD Parent Diagnostic Interview for DSM-5 Full (KSADS-5)	ksads_13_929_p, ksads_13_930_p, ksads_13_931_p, ksads_13_932_p, ksads_13_933_p, ksads_13_934_p, ksads_13_935_p, ksads_13_936_p, ksads_13_937_p, ksads_13_938_p, ksads_13_939_p, ksads_13_940_p

Abbreviations: DTI, diffusion tensor imaging; FA, fractional anisotropy; MD, mean diffusivity; MRI, magnetic resonance imaging; RND, restricted normalized directional; RNI, restricted normalized isotropic; RSI, restriction spectrum imaging

[a] Data elements are listed in the they appear in the ABCD Data Dictionary at https://nda.nih.gov/data_dictionary.html?source=ABCD%2BRelease%2B4.0.

© 2023 Li ZA et al. *JAMA Network Open*.

eTable 3. Factor Loadings of ADI Constructs[a]

**Area Deprivation Index (ADI) construct description**	**Factor loading**	**Inclusion in neighborhood disadvantage**
**Percentage of population aged ≥ 25 years with < 9 years of education**	0.63	Yes
**Percentage of population aged ≥ 25 years with at least a high school diploma**	0.77[b]	Yes
Percentage of employed persons aged ≥ 16 years in white collar occupations	0.18[b]	No
**Median family income**	0.79[b]	Yes
**Income disparity, defined by Singh as the log of 100 × ratio of the number of households with < $10000 annual income to the number of households with > $50000 annual income.**	0.86	Yes
Median home value	0.45[b]	No
Median gross rent	0.52	No
Median monthly mortgage	0.51	No
**Percentage of homeowners**	0.75[b]	Yes
Percentage of occupied housing units with > 1 person per room (i.e., crowding)	0.52	No
**Percentage of civilian labor force aged ≥ 16 years unemployed (i.e., unemployment rate)**	0.73	Yes
**Percentage of families below the poverty level**	0.94	Yes
**Percentage of population below 138% of the poverty threshold**	0.98	Yes
**Percentage of single parent households**	0.83	Yes
**Percentage of occupied housing units without a motor vehicle**	0.70	Yes
Percentage of occupied housing units without a telephone	0.40	No
Percentage of occupied housing units without complete plumbing	0.22	No

[a] An exploratory factor analysis based on 7912 participants with complete data of all 17 Area Deprivation Index (ADI) metrics suggested a one-factor solution; factors (in bold) with strong loadings (≥ 0.63) were included in the neighborhood disadvantage variable. Kaiser-Meyer-Olkin (KMO) factor adequacy = 0.9; Bartlett's test of sphericity chi-sq = 131708.7, *p* < .001; and positive determinant = 5.802e-08 all suggested factorability.

[b]Metric was reverse-coded to give consistent direction amongst metrics such that higher neighborhood disadvantage reflected greater impoverishment.

© 2023 Li ZA et al. *JAMA Network Open*.

eFigure 2. Isosurface Renderings of White Matter Tracts

A **3D visualization of all tracts in AtlasTrack atlas** (to relative scale)

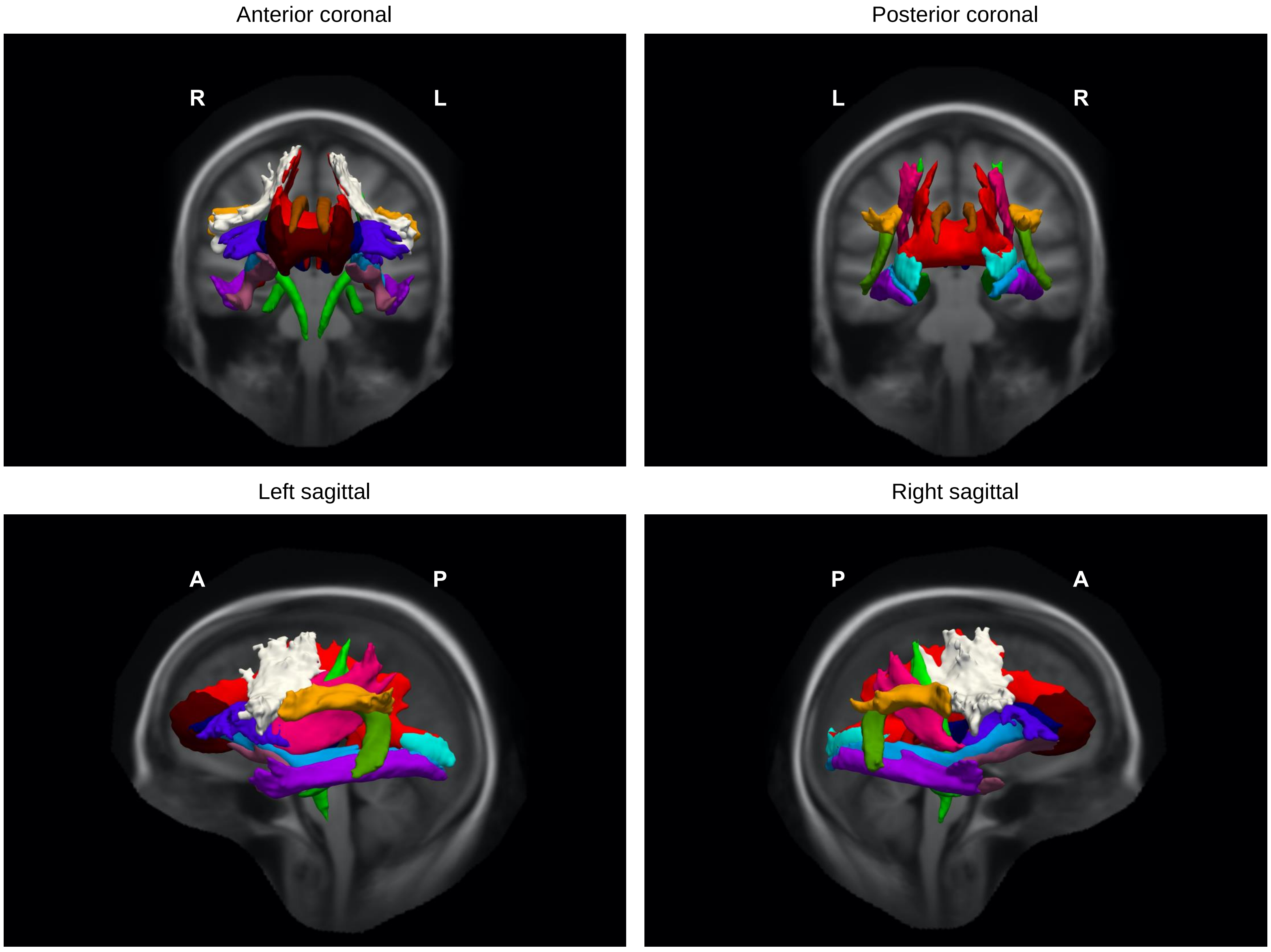

B **3D visualization of individual tracts in AtlasTrack atlas** (not to scale; *left*, anterior coronal view; *right*, left sagittal view)

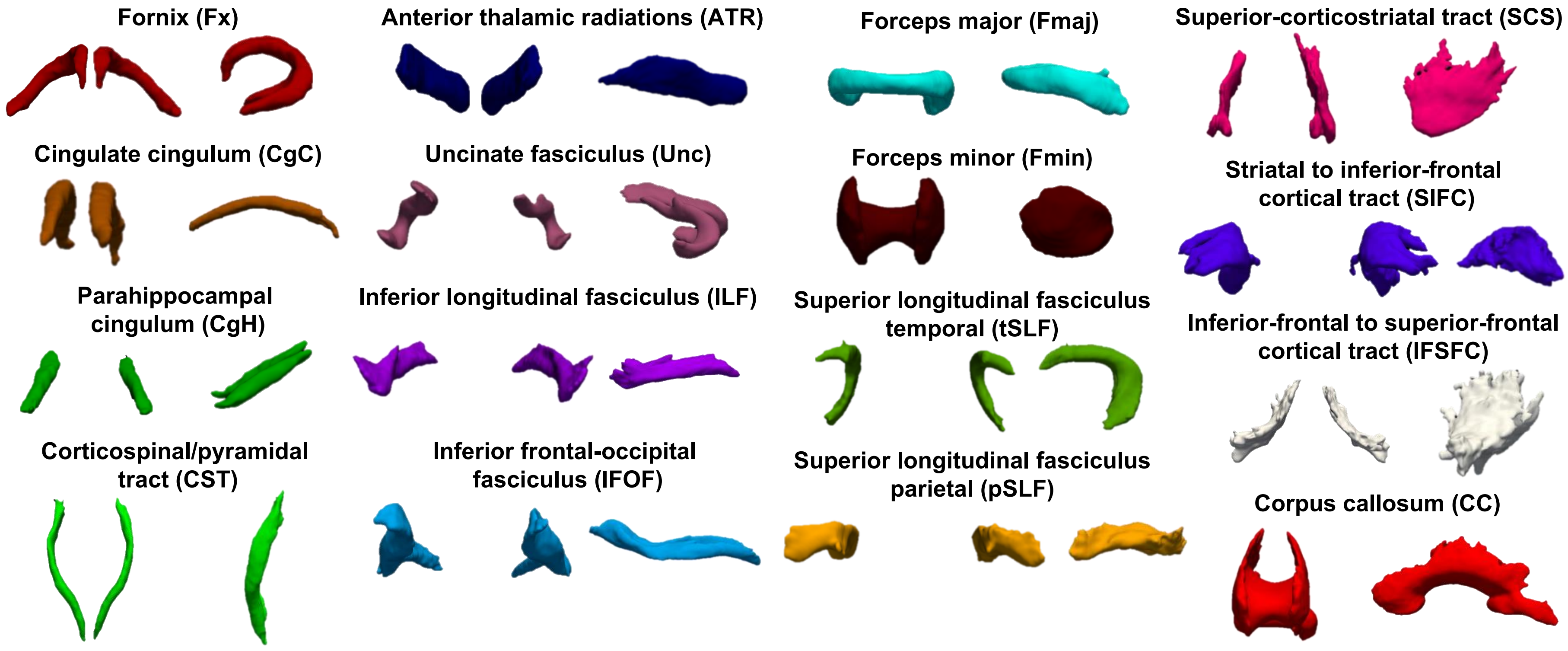

Note. Coronal and sagittal views of all (in scale relative to each other) and individual major white matter tracts (each scaled differently for ease of visualization). The forceps major (Fmaj) and forceps minor (Fmin) are portions of the corpus callosum (CC). The superior longitudinal fasciculus (not shown) was partitioned into temporal (tSLF) and parietal (pSLF) subregions for each hemisphere. R, right; L, left; A, anterior; P, posterior.

© 2023 Li ZA et al. *JAMA Network Open*.

eFigure 3. Relationships Between Missingness of Key Variables[a]

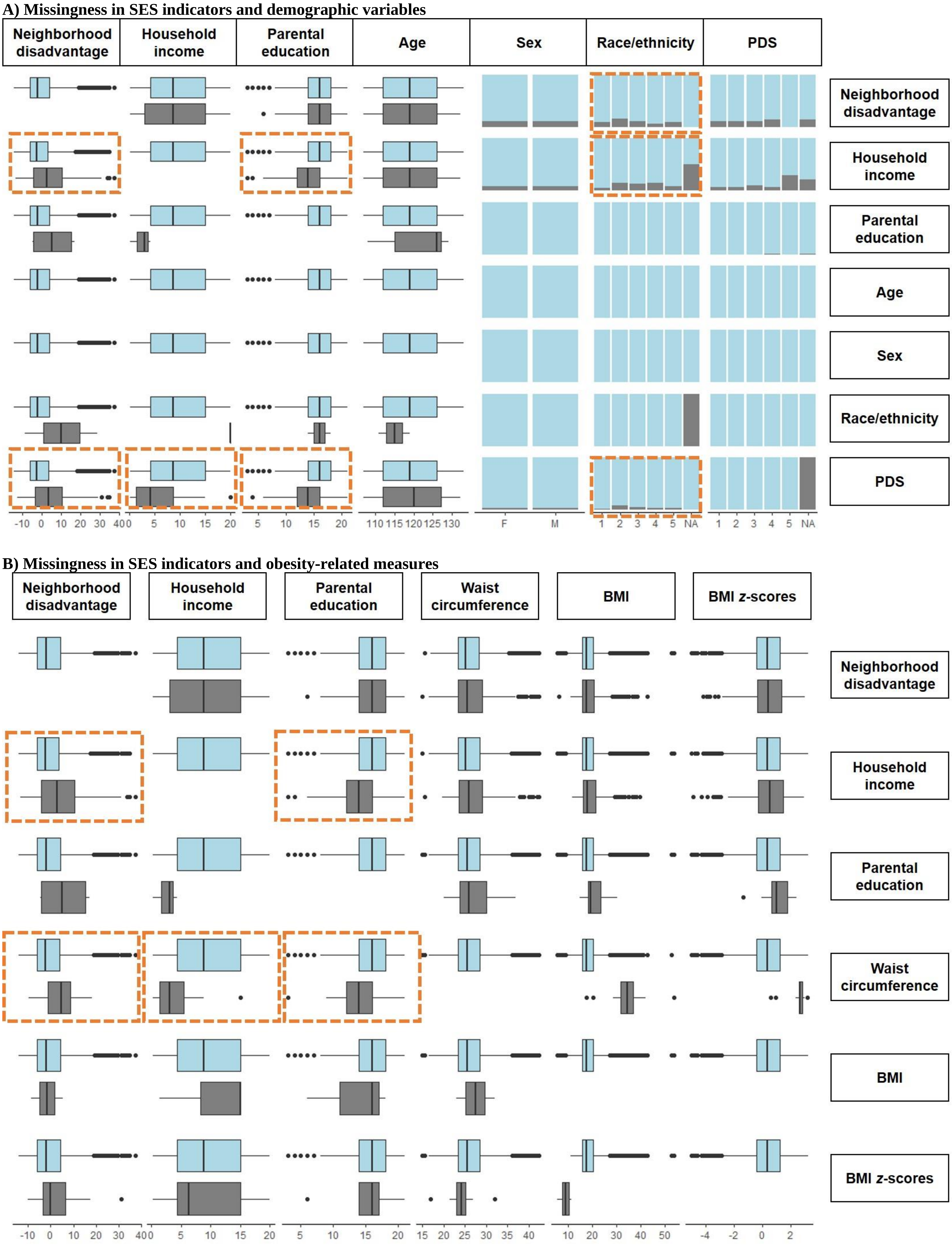

© 2023 Li ZA et al. *JAMA Network Open*.

C) Missingness in SES indicators and cognition

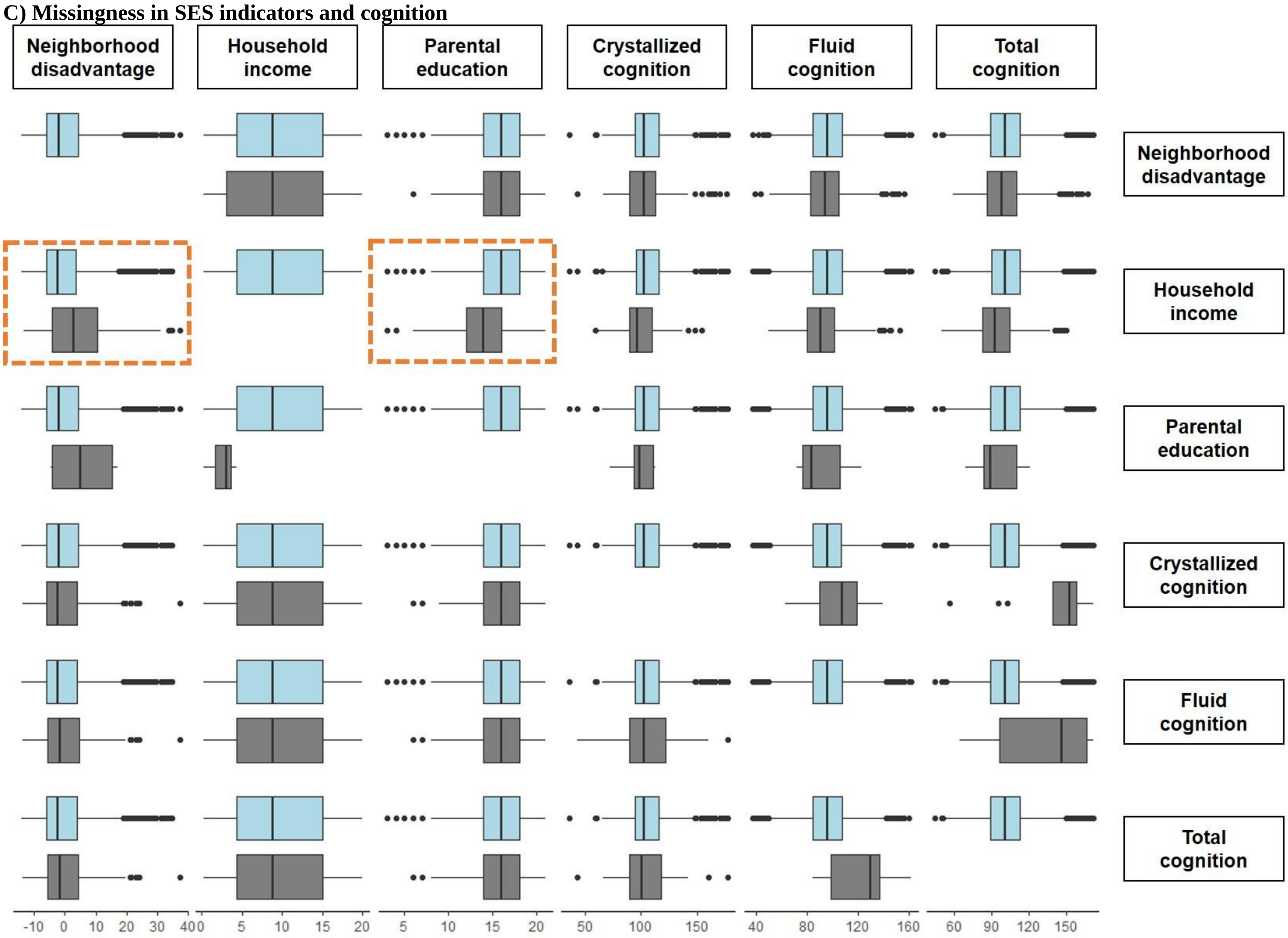

Abbreviations: BMI, body mass index; PDS, pubertal development stage; SES, socioeconomic status

[a] While all relevant variables in the current study had at most 8.29% of missing cases (see **eTable 4** in the **Supplement** for tally), selecting only complete data would trim our sample to 6513 participants (a 26% reduction), considerably hampering statistical power. Furthermore, analyses using the "finalfit" package[18], as shown here, suggested some associations between missingness of SES indicators and demographics. Missingness was represented by grey color. Each column shows the distribution of a variable grouped by whether the row variable data is present (in blue) or missing (in gray). Instances where the missingness of the row variable appeared to be confounded by levels of the column variable are outlined in orange. For example, in panel A, the missingness in neighborhood disadvantage (row 1) appeared unevenly distributed across race/ethnicity groups (column 6). Similarly, participants with missing household income data (row 2) had greater neighborhood disadvantage (column 1), lower parental education (column 3), and were disproportionally of non-White race/ethnicity (column 6). Panel B shows that participants with missing waist circumference data (row 4) had greater neighborhood disadvantage, lower household income, and lower parental education. These data suggested that the missingness in SES data was related to demographics, and that the missingness in waist circumference was related to SES. In comparison, missingness in cognitive performance (panel C) was unrelated to SES, and so was the case for neuroimaging metrics (not shown here for brevity). We did not examine variables with very low missingness (e.g., race/ethnicity had only two missing cases) as statistical inference was likely to be unreliable.

© 2023 Li ZA et al. *JAMA Network Open*.

eTable 4. Missing Data and Roles of Variables in Imputation[a]

Variable	**Missing cases** (out of 8842)	Imputed?	Predictor in imputation?
**Age**	0 (0%)	No	Yes
**Sex**	0 (0%)	No	Yes
**Race/ethnicity**	2 (0.02%)	Yes	Yes
**PDS**	311 (3.52%)	Yes	Yes
**Scanner ID**	0 (0%)	No	No
**Family ID**	0 (0%)	No	No
**Neighborhood disadvantage**	930 (10.52%)	Yes	Yes
**Household income**	733 (8.29%)	Yes	Yes
**Parental education**	9 (0.10%)	Yes	Yes
**White matter microstructure**			
**RSI-RND**	1 – 25 (0.01% – 0.28%)	No	Yes
**RSI-RNI**	6 – 59 (0.07% – 0.67%)	No	Yes
**DTI-FA**	1 – 11 (0.01% – 0.12%)	No	Yes
**DTI-MD**	2 – 44 (0.02% – 0.50%)	No	Yes
**Mean head motion**	0 (0%)	No	Yes
**ICV**	0 (0%)	No	Yes
**Obesity-related measures**[b]			
**Waist circumference**	23 (0.26%)	Yes	Yes
**BMI**	3 (0.03%)	Yes	Yes
**BMI *z*-score**	23 (0.26%)	Yes	Yes
**Cognition**			
**Flanker inhibitory control**	123 (1.39%)	No	Yes
**Picture sequencing**	117 (1.32%)	No	Yes
**List sorting working memory**	148 (1.67%)	No	Yes
**Picture vocabulary**	129 (1.46%)	No	Yes
**Oral reading**	149 (1.69%)	No	Yes
**Dimensional change card sort**	131 (1.48%)	No	Yes
**Pattern comparison**	132 (1.49%)	No	Yes
**Crystallized cognition composite**	276 (3.12%)	No	Yes
**Fluid cognition composite**	302 (3.42%)	No	Yes
**Total cognition composite**	302 (3.42%)	No	Yes
**ACE total number**	133 (1.50%)	No	No
**Common psychiatric diagnoses status**	0 (0%)	No	No
**Preterm birth status**	92 (1.04%)	No	No

Abbreviations: ACE, adverse childhood experience; BMI, body mass index; DTI, diffusion tensor imaging; FA, fractional anisotropy; ICV, intracranial volume; MD, mean diffusivity; PDS, pubertal development stage; RND, restricted normalized directional; RNI, restricted normalized isotropic; RSI, restriction spectrum imaging

[a] Missing cases are reported as count (frequency). White matter microstructure and cognitive variables were not imputed as they served as outcome variables in analyses; other variables that were not imputed include those that did not have missing cases and those that were used only in sensitivity analyses. To reduce computational cost, Scanner ID and family ID were not modeled as predictors used to impute missing cases.

[b] While we had excluded participants with missing data on obesity-related measures, these missing data were introduced due to outlier removal.

© 2023 Li ZA et al. *JAMA Network Open*.

eTable 5. Distribution of Imputed Variables Before and After Imputation of Missing Data[a]

Variable	Original (non-imputed) dataset	Multiply imputed datasets
Demographic variables		
**Race/ethnicity**		
White	4738 (53.6%)	4739 (53.6%)
Black	1212 (13.7%)	1213 (13.7%)
Hispanic	1805 (20.4%)	1805 (20.4%)
Asian	183 (2.1%)	183 (2.1%)
Other	902 (10.2%)	902 (10.2%)
**PDS**		
Pre-puberty	4425 (51.9%)	4561 (51.6%)
Early puberty	2009 (23.5%)	2088 (23.6%)
Mid-puberty	1966 (23.0%)	2054 (23.2%)
Late puberty	124 (1.5%)	131 (1.5%)
Post-puberty	7 (0.1%)	7 (0.1%)
SES indicators		
**Neighborhood disadvantage**	0 ± 8.2 (range: -14.3 to 37.5)	0 ± 8.3 (range: -14.3 to 37.5)
**Household income**	10.0 ± 6.2 (range: 0.3 to 20.0)	9.8 ± 6.2 (range: 0.3 to 20.0)
**Parental education (years)**	15.9 ± 2.8 (range: 3.0 to 21.0)	15.9 ± 2.8 (range: 3.0 to 21.0)
Obesity-related measures		
**Waist circumference (in)**	26.3 ± 4.0 (range: 15.0 to 42.5)	26.3 ± 4.0 (range: 15.0 to 42.5)
**BMI (kg/m^2)**	18.6 ± 4.1 (range: 5.3 to 53.9)	18.6 ± 4.1 (range: 5.3 to 53.9)
**BMI *z*-score**	0.4 ± 1.2 (range: -5.0 to 3.2)	0.4 ± 1.2 (range: -5.0 to 3.2)

Abbreviations: BMI, body mass index; PDS, pubertal development stage; SES, socioeconomic status

[a] Distributions of imputed demographic variables, SES indicators, and obesity-related measures were consistent before and after imputation of missing data. Results are shown as mean ± standard deviation for continuous variables and count (frequency) for categorical variables. Variables that were not imputed are not shown here.

eTable 6. Associations Between Preserved Variables and Pre- and Postharmonization RSI and DTI Measurements[a]

Variable (IV)	Association with RSI and DTI metrics (DVs) before harmonization, R^2 (%)				Association with RSI and DTI metrics (DVs) after harmonization, R^2 (%)			
	Mean	SD	Min	Max	Mean	SD	Min	Max
**Age**	1.33	1.04	0.01	4.61	1.84	1.36	-0.01	5.63
**Sex**	0.75	1.05	-0.01	3.73	0.95	1.22	-0.01	4.32
**Race/ethnicity**	0.67	0.45	0	2.58	0.68	0.59	-0.02	3.05
**PDS**	0.54	0.65	-0.04	3.16	0.74	0.87	-0.04	3.51
**Neighborhood disadvantage**	0.40	0.43	-0.01	1.60	0.26	0.32	-0.01	1.45
**Household income**	0.18	0.17	-0.01	1.00	0.31	0.24	-0.01	0.94
**Parental education**	0.22	0.22	-0.01	1.21	0.30	0.29	-0.01	1.20

Abbreviations: DTI, diffusion tensor imaging; DV, dependent variable; IV, independent variable; PDS, pubertal development stage; RSI, restriction spectrum imaging; SD, standard deviation

[a] Associations between preserved variables and RSI and DTI metrics seemed only limitedly affected by ComBat harmonization. Associations were estimated in linear regression models in which each of the RSI and DTI metrics (as DV) was regressed onto each of the IVs. The post-harmonization associations were tested in a randomly chosen dataset out of 50 imputed datasets.

© 2023 Li ZA et al. *JAMA Network Open*.

eTable 7. Associations Between SES and Race and Ethnicity[a]

Race/ethnicity (IV)	SES indicator (DV)					
	**Neighborhood disadvantage** (higher = lower SES)		**Household income** (higher = higher SES)		**Parental education** (higher = higher SES)	
	β	**Partial R^2**	***β***	**Partial R^2**	***β***	**Partial R^2**
**Black**	1.26 ***	0.17	-1.07 ***	0.13	-0.74 ***	0.06
**Hispanic**	0.88 ***	0.13	-0.75 ***	0.10	-0.64 ***	0.06
**Asian**	-0.05	< 0.01	0.13 *	< 0.01	0.29 ***	< 0.01
**Other**	0.36 ***	0.02	-0.31 ***	0.01	-0.20 ***	< 0.01

Abbreviations: DV, dependent variable; IV, independent variable; SES, socioeconomic status

[a] SES was highly entangled with race/ethnicity. Comparing association magnitudes across the three SES indicators, it appears that neighborhood disadvantage is most confounded with race/ethnicity, followed by household income and parental education. This may explain why many white matter microstructure associations with neighborhood disadvantage and household income diminished more in magnitude once analyses controlled for race/ethnicity (**eTable 11** in the **Supplement**). Analyses were linear regression models where race/ethnicity was the IV of interest and each of the SES indicator was the DV. Results were in reference to White participants and were standardized. *, $p < .05$; ***, $p < .001$.

eTable 8. Sample Sizes for Models Testing Associations Between SES, RSI and DTI Measurements, and Cognitive Performance[a]

**Metric/Region**	**Sample size (*n*)**			
**White matter tract**	**RSI-RND**	**RSI-RNI**	**DTI-FA**	**DTI-MD**
Fx (right)	8832	8812	8840	8807
Fx (left)	8829	8793	8841	8799
CgC (right)	8837	8815	8839	8832
CgC (left)	8826	8803	8839	8829
CgH (right)	8836	8819	8838	8814
CgH (left)	8828	8799	8836	8818
CST (right)	8824	8825	8837	8827
CST (left)	8823	8817	8837	8822
ATR (right)	8839	8789	8839	8823
ATR (left)	8833	8786	8838	8821
Unc (right)	8830	8826	8833	8837
Unc (left)	8824	8826	8831	8833
ILF (right)	8830	8834	8840	8839
ILF (left)	8834	8829	8838	8836
IFOF (right)	8835	8830	8838	8834
IFOF (left)	8839	8801	8838	8831
Fmaj	8822	8798	8832	8815
Fmin	8824	8819	8832	8814
CC	8824	8820	8835	8831
SLF (right)	8830	8835	8839	8840
SLF (left)	8822	8824	8838	8839
tSLF (right)	8830	8835	8840	8839
tSLF (left)	8826	8821	8839	8838
pSLF (right)	8827	8836	8839	8840
pSLF (left)	8817	8826	8835	8838
SCS (right)	8841	8830	8840	8839
SCS (left)	8839	8824	8840	8838
SIFC (right)	8838	8829	8839	8830
SIFC (left)	8840	8828	8838	8818
IFSFC (right)	8829	8833	8840	8838
IFSFC (left)	8826	8833	8839	8837
**Cognition**	**Score**			
Picture vocabulary	8713			
Flanker inhibitory control	8719			
List sorting	8694			
Dimensional card sort	8711			
Pattern comparison	8710			
Picture sequence	8725			
Oral reading	8693			
Fluid cognition	8540			
Crystallized cognition	8566			
Total cognition	8540			

Abbreviations: ATR, anterior thalamic radiations; CC, corpus callosum; CgC, cingulate cingulum; CgH, parahippocampal cingulum; CST, corticospinal/pyramidal tract; DTI, diffusion tensor imaging; FA, fractional anisotropy; Fmaj, forceps major; Fmin, forceps minor; Fx, fornix; IFOF, inferior frontal-occipital fasciculus; IFSFC, inferior-frontal to superior-frontal cortical tract; ILF, inferior longitudinal fasciculus; MD, mean diffusivity; pSLF, parietal superior longitudinal fasciculus; RND, restricted normalized directional; RNI, restricted normalized isotropic; RSI, restriction spectrum imaging; SCS, superior-corticostriatal tract; SIFC, striatal to inferior-frontal cortical tract; SLF, superior longitudinal fasciculus; tSLF, temporal superior longitudinal fasciculus; Unc, uncinate fasciculus

[a] Sample sizes for models testing associations between SES and RSI/DTI metrics and cognitive performance varied as both imaging and cognitive measures had unimputed missing values. In comparison, models testing associations between SES and obesity-related measures had sample sizes of 8824 due to complete imputation.

© 2023 Li ZA et al. *JAMA Network Open*.

eFigure 4. Correlations Between SES Factors

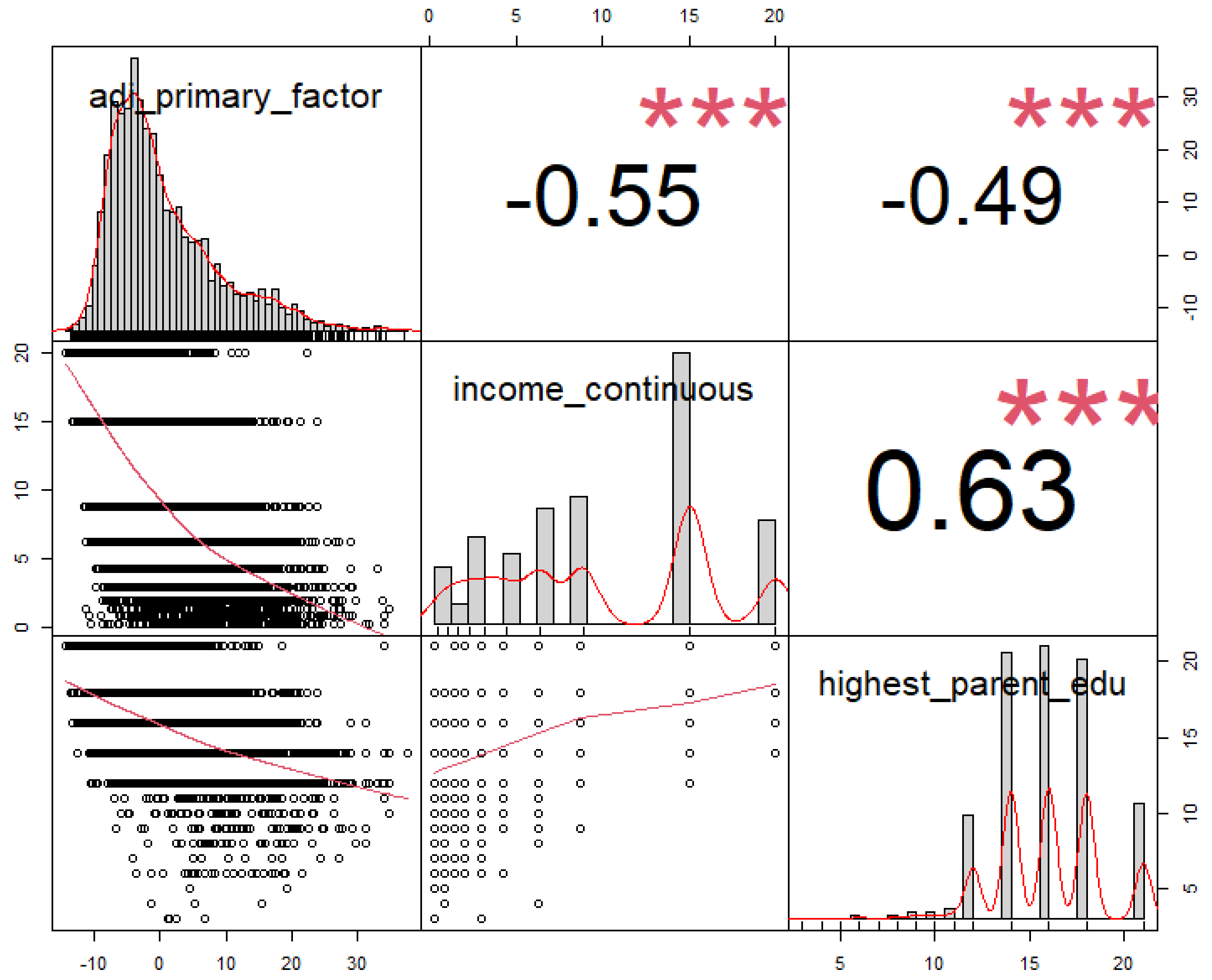

Note. Moderate-to-high bivariate correlations (Pearson's *r*) were observed between socioeconomic status (SES) indicators (neighborhood disadvantage (adi_primary_factor); household income (income_continuous); parental education (highest_parent_edu)) in a randomly selected multiply imputed dataset out of 50. ***, $p < .001$.

© 2023 Li ZA et al. *JAMA Network Open*.

eTable 9. Associations Between SES and White Matter Microstructure

White matter tracts	SES indicators (IVs)								
	Neighborhood disadvantage (higher = lower SES)			Household income (higher = higher SES)			Parental education (higher = higher SES)		
	β **(95% CI)**	***p*-value** (nominal)	***p*-value** (FDR)	***β*** **(95% CI)**	***p*-value** (nominal)	***p*-value** (FDR)	***β*** **(95% CI)**	***p*-value** (nominal)	***p*-value** (FDR)
**A) RSI-RND (DV)**									
Fx (right)	0.001 (-0.026 to 0.028)	.94	.97	0.024 (-0.007 to 0.055)	.13	.51	0.026 (-0.003 to 0.054)	.08	.15
Fx (left)	-0.009 (-0.037 to 0.018)	.50	.68	0.014 (-0.018 to 0.045)	.39	.56	0.031 (0.002 to 0.060)	.04	.09
CgC (right)	0.032 (0.005 to 0.059)	.02	.11	-0.033 (-0.064 to -0.003)	.03	.39	0.010 (-0.018 to 0.038)	.48	.55
CgC (left)	0.017 (-0.010 to 0.044)	.21	.45	-0.023 (-0.053 to 0.007)	.13	.51	-0.013 (-0.041 to 0.015)	.37	.50
CgH (right)	-0.013 (-0.040 to 0.014)	.34	.52	-0.010 (-0.040 to 0.021)	.54	.73	0.033 (0.005 to 0.061)	.02	.08
CgH (left)	-0.002 (-0.029 to 0.024)	.87	.93	0.015 (-0.015 to 0.045)	.32	.54	0.030 (0.002 to 0.058)	.04	.09
CST (right)	0.012 (-0.014 to 0.039)	.35	.52	0.013 (-0.017 to 0.043)	.39	.56	0.042 (0.015 to 0.069)	.003	**.01**
CST (left)	0.026 (0.000 to 0.053)	.05	.21	0.007 (-0.023 to 0.037)	.64	.83	0.049 (0.021 to 0.077)	.001	**.004**
ATR (right)	0.030 (0.003 to 0.057)	.03	.16	0.019 (-0.012 to 0.050)	.24	.52	0.018 (-0.010 to 0.047)	.21	.29
ATR (left)	0.007 (-0.021 to 0.035)	.61	.79	0.026 (-0.005 to 0.057)	.10	.51	0.019 (-0.010 to 0.047)	.19	.29
Unc (right)	0.024 (-0.003 to 0.051)	.08	.26	-0.046 (-0.076 to 0.016)	.002	.07	0.009 (-0.018 to 0.037)	.50	.56
Unc (left)	0.029 (0.001 to 0.056)	.04	.18	-0.032 (-0.062 to -0.002)	.04	.39	0.010 (-0.018 to 0.038)	.47	.55
ILF (right)	0.002 (-0.025 to 0.029)	.87	.93	-0.016 (-0.046 to 0.014)	.30	.54	0.021 (-0.007 to 0.048)	.15	.24
ILF (left)	0.019 (-0.008 to 0.046)	.17	.44	-0.001 (-0.031 to 0.029)	.94	.98	0.020 (-0.008 to 0.047)	.16	.25
IFOF (right)	0.004 (-0.024 to 0.030)	.79	.93	-0.028 (-0.058 to 0.002)	.07	.51	0.026 (-0.002 to 0.054)	.06	.13
IFOF (left)	0.002 (-0.024 to 0.029)	.86	.93	-0.020 (-0.050 to 0.009)	.18	.52	0.027 (-0.001 to 0.054)	.06	.12
Fmaj	-0.040 (-0.067 to -0.013)	.004	**.03**	0.022 (-0.008 to 0.053)	.15	.51	-0.012 (-0.040 to 0.016)	.40	.52
Fmin	0.019 (-0.008 to 0.045)	.17	.44	-0.018 (-0.048 to 0.012)	.24	.52	-0.010 (-0.038 to 0.017)	.47	.55
CC	-0.014 (-0.041 to 0.012)	.29	.50	0.001 (-0.029 to 0.030)	.97	.98	-0.004 (-0.032 to 0.023)	.75	.77
SLF (right)	-0.017 (-0.044 to 0.010)	.22	.45	-0.001 (-0.031 to 0.029)	.96	.98	0.053 (0.025 to 0.080)	< .001	**.002**
SLF (left)	-0.055 (-0.081 to -0.028)	< .001	**.001**	0.014 (-0.016 to 0.044)	.35	.55	0.050 (0.022 to 0.077)	< .001	**.003**
tSLF (right)	-0.018 (-0.045 to 0.009)	.19	.44	0.000 (-0.031 to 0.030)	.98	.98	0.044 (0.016 to 0.072)	.002	**.01**
tSLF (left)	-0.056 (-0.083 to -0.030)	< .001	**.001**	0.019 (-0.011 to 0.049)	.22	.52	0.042 (0.015 to 0.070)	.003	**.01**
pSLF (right)	-0.018 (-0.045 to 0.009)	.19	.44	-0.001 (-0.031 to 0.029)	.96	.98	0.054 (0.026 to 0.081)	< .001	**.002**
pSLF (left)	-0.040 (-0.066 to 0.013)	.003	**.03**	0.003 (-0.027 to 0.032)	.86	.98	0.059 (0.032 to 0.086)	< .001	**.001**
SCS (right)	0.015 (-0.012 to 0.042)	.27	.50	-0.015 (-0.046 to 0.015)	.33	.54	0.032 (0.004 to 0.060)	.03	.09
SCS (left)	0.000 (-0.026 to 0.027)	.98	.98	0.004 (-0.025 to 0.034)	.77	.95	0.030 (0.002 to 0.058)	.03	.09
SIFC (right)	0.014 (-0.013 to 0.042)	.31	.51	-0.023 (-0.054 to 0.007)	.13	.51	-0.009 (-0.037 to 0.019)	.52	.56
SIFC (left)	-0.003 (-0.030 to 0.024)	.81	.93	-0.017 (-0.047 to 0.012)	.25	.52	-0.004 (-0.032 to 0.024)	.77	.77
IFSFC (right)	-0.011 (-0.038 to 0.015)	.40	.57	0.019 (-0.010 to 0.049)	.20	.52	0.020 (-0.007 to 0.048)	.14	.24
IFSFC (left)	-0.014 (-0.040 to 0.012)	.29	.50	0.015 (-0.015 to 0.044)	.33	.54	0.021 (-0.006 to 0.049)	.12	.22

© 2023 Li ZA et al. *JAMA Network Open*.

White matter tracts	SES indicators (IVs)								
	**Neighborhood disadvantage** (higher = lower SES)			**Household income** (higher = higher SES)			**Parental education** (higher = higher SES)		
	***β* (95% CI)**	***p*-value** (nominal)	***p*-value** (FDR)	***β* (95% CI)**	***p*-value** (nominal)	***p*-value** (FDR)	***β* (95% CI)**	***p*-value** (nominal)	***p*-value** (FDR)
**B) RSI-RNI (DV)**									
Fx (right)	0.046 (0.019 to 0.074)	.001	**.01**	0.008 (-0.023 to 0.040)	.59	.63	-0.013 (-0.042 to 0.016)	.38	.95
Fx (left)	0.047 (0.020 to 0.074)	.001	**.01**	-0.005 (-0.036 to 0.027)	.78	.78	-0.015 (-0.044 to 0.014)	.31	.95
CgC (right)	0.026 (0.000 to 0.053)	.05	.18	-0.033 (-0.063 to -0.003)	.03	**.05**	-0.012 (-0.041 to 0.016)	.39	.95
CgC (left)	0.019 (-0.009 to 0.046)	.18	.52	-0.036 (-0.067 to -0.005)	.02	**.04**	-0.018 (-0.046 to 0.011)	.23	.95
CgH (right)	0.061 (0.034 to 0.088)	< .001	**< .001**	-0.031 (-0.062 to -0.001)	.04	**.05**	0.006 (-0.022 to 0.034)	.68	.95
CgH (left)	0.066 (0.039 to 0.094)	< .001	**< .001**	-0.050 (-0.081 to -0.020)	.001	**.01**	-0.002 (-0.030 to 0.026)	.88	.95
CST (right)	0.037 (0.010 to 0.065)	.01	**.03**	-0.031 (-0.062 to -0.001)	.05	**.05**	-0.001 (-0.029 to 0.027)	.95	.95
CST (left)	0.042 (0.015 to 0.069)	.002	**.01**	-0.044 (-0.074 to -0.013)	.01	**.01**	-0.003 (-0.031 to 0.025)	.83	.95
ATR (right)	0.045 (0.018 to 0.072)	.001	**.01**	-0.045 (-0.075 to -0.014)	.004	**.01**	0.003 (-0.026 to 0.031)	.85	.95
ATR (left)	0.039 (0.012 to 0.066)	.005	**.02**	-0.040 (-0.071 to -0.009)	.01	**.02**	-0.013 (-0.041 to 0.015)	.36	.95
Unc (right)	0.022 (-0.005 to 0.049)	.11	.36	-0.062 (-0.092 to -0.031)	< .001	**.002**	0.005 (-0.023 to 0.034)	.71	.95
Unc (left)	0.015 (-0.012 to 0.042)	.28	.57	-0.052 (-0.083 to -0.022)	.001	**.01**	-0.003 (-0.031 to 0.026)	.86	.95
ILF (right)	-0.004 (-0.031 to 0.022)	.75	.97	-0.042 (-0.073 to -0.012)	.01	**.01**	-0.003 (-0.031 to 0.025)	.83	.95
ILF (left)	-0.001 (-0.028 to 0.026)	.93	.97	-0.033 (-0.063 to -0.002)	.04	**.05**	-0.014 (-0.042 to 0.014)	.33	.95
IFOF (right)	0.001 (-0.026 to 0.028)	.94	.97	-0.056 (-0.086 to -0.026)	< .001	**.004**	-0.007 (-0.034 to 0.021)	.65	.95
IFOF (left)	0.002 (-0.024 to 0.029)	.87	.97	-0.052 (-0.083 to -0.022)	.001	**.01**	-0.011 (-0.039 to 0.017)	.43	.95
Fmaj	0.002 (-0.025 to 0.030)	.88	.97	-0.005 (-0.036 to 0.025)	.73	.75	-0.048 (-0.077 to -0.020)	.001	**.03**
Fmin	0.015 (-0.013 to 0.042)	.29	.57	-0.045 (-0.076 to -0.013)	.005	**.01**	-0.039 (-0.068 to -0.010)	.01	.13
CC	0.004 (-0.024 to 0.031)	.80	.97	-0.037 (-0.068 to -0.006)	.02	**.03**	-0.025 (-0.053 to 0.004)	.09	.89
SLF (right)	-0.004 (-0.030 to 0.023)	.78	.97	-0.032 (-0.061 to -0.002)	.04	**.05**	-0.006 (-0.042 to 0.022)	.67	.95
SLF (left)	0.014 (-0.012 to 0.041)	.29	.57	-0.045 (-0.075 to -0.015)	.004	**.01**	-0.001 (-0.029 to 0.027)	.94	.95
tSLF (right)	-0.007 (-0.034 to 0.019)	.58	.95	-0.026 (-0.056 to 0.004)	.09	.10	-0.001 (-0.029 to 0.026)	.92	.95
tSLF (left)	0.013 (-0.014 to 0.040)	.33	.61	-0.044 (-0.074 to -0.014)	.004	**.01**	0.002 (-0.026 to 0.029)	.91	.95
pSLF (right)	-0.001 (-0.028 to 0.025)	.91	.97	-0.033 (-0.063 to -0.003)	.03	**.04**	-0.005 (-0.033 to 0.022)	.70	.95
pSLF (left)	0.016 (-0.011 to 0.043)	.23	.57	-0.046 (-0.077 to -0.016)	.003	**.01**	0.002 (-0.026 to 0.029)	.91	.95
SCS (right)	-0.016 (-0.042 to 0.011)	.24	.57	-0.022 (-0.052 to 0.008)	.15	.17	0.009 (-0.019 to 0.037)	.52	.95
SCS (left)	0.002 (-0.024 to 0.029)	.86	.97	-0.040 (-0.069 to -0.010)	.01	**.02**	0.004 (-0.024 to 0.031)	.79	.95
SIFC (right)	-0.008 (-0.035 to 0.019)	.57	.95	-0.045 (-0.076 to -0.014)	.004	**.01**	-0.007 (-0.035 to 0.022)	.65	.95
SIFC (left)	0.000 (-0.027 to 0.027)	.99	.99	-0.038 (-0.069 to -0.007)	.02	**.03**	-0.009 (-0.038 to 0.019)	.52	.95
IFSFC (right)	0.003 (-0.024 to 0.029)	.85	.97	-0.046 (-0.077 to -0.016)	.003	**.01**	0.008 (-0.019 to 0.036)	.55	.95
IFSFC (left)	-0.003 (-0.030 to 0.024)	.84	.97	-0.046 (-0.077 to -0.015)	.003	**.01**	0.011 (-0.017 to 0.039)	.44	.95

© 2023 Li ZA et al. *JAMA Network Open*.

White matter tracts	SES indicators (IVs)								
	**Neighborhood disadvantage** (higher = lower SES)			**Household income** (higher = higher SES)			**Parental education** (higher = higher SES)		
	***β* (95% CI)**	***p*-value** (nominal)	***p*-value** (FDR)	***β* (95% CI)**	***p*-value** (nominal)	***p*-value** (FDR)	***β* (95% CI)**	***p*-value** (nominal)	***p*-value** (FDR)
**C) DTI-FA (DV)**									
Fx (right)	-0.011 (-0.038 to 0.017)	.45	.57	0.029 (-0.002 to 0.060)	.07	.35	0.027 (-0.002 to 0.055)	.07	.13
Fx (left)	-0.024 (-0.051 to 0.003)	.09	.22	0.008 (-0.023 to 0.039)	.60	.93	0.042 (0.013 to 0.070)	.004	**.01**
CgC (right)	0.028 (0.000 to 0.055)	.05	.18	-0.041 (-0.072 to 0.010)	.01	.27	0.007 (-0.021 to 0.035)	.63	.75
CgC (left)	0.017 (-0.010 to 0.044)	.22	.43	-0.028 (-0.059 to 0.002)	.07	.35	-0.013 (-0.041 to 0.015)	.37	.50
CgH (right)	-0.015 (-0.042 to 0.012)	.28	.48	-0.001 (-0.031 to 0.030)	.96	.96	0.011 (-0.017 to 0.039)	.45	.57
CgH (left)	-0.010 (-0.037 to 0.017)	.47	.57	0.021 (-0.009 to 0.051)	.17	.67	0.028 (0.000 to 0.057)	.05	.11
CST (right)	0.012 (-0.014 to 0.039)	.36	.56	-0.003 (-0.033 to 0.027)	.86	.96	0.045 (0.018 to 0.073)	.001	**.01**
CST (left)	0.031 (0.005 to 0.058)	.02	.10	-0.001 (-0.032 to 0.029)	.92	.96	0.047 (0.019 to 0.075)	.001	**.01**
ATR (right)	0.025 (-0.002 to 0.052)	.07	.22	0.013 (-0.018 to 0.044)	.43	.79	0.021 (-0.007 to 0.050)	.15	.24
ATR (left)	0.005 (-0.023 to 0.033)	.74	.79	0.030 (-0.001 to 0.062)	.06	.35	0.021 (-0.008 to 0.049)	.15	.24
Unc (right)	0.018 (-0.010 to 0.046)	.21	.43	-0.035 (-0.066 to 0.005)	.02	.35	0.002 (-0.026 to 0.030)	.88	.88
Unc (left)	0.012 (-0.016 to 0.040)	.42	.57	-0.020 (-0.051 to 0.011)	.21	.69	0.006 (-0.022 to 0.035)	.67	.77
ILF (right)	-0.001 (-0.028 to 0.026)	.93	.93	-0.004 (-0.035 to 0.026)	.78	.96	0.019 (-0.009 to 0.047)	.17	.24
ILF (left)	0.012 (-0.016 to 0.039)	.40	.57	0.009 (-0.022 to 0.040)	.57	.93	0.020 (-0.008 to 0.048)	.16	.24
IFOF (right)	0.005 (-0.022 to 0.032)	.73	.79	-0.025 (-0.056 to 0.005)	.10	.44	0.031 (0.003 to 0.059)	.03	.07
IFOF (left)	0.010 (-0.017 to 0.037)	.48	.57	-0.007 (-0.037 to 0.023)	.63	.93	0.028 (0.000 to 0.056)	.05	.11
Fmaj	-0.024 (-0.052 to 0.003)	.08	.22	0.013 (-0.017 to 0.044)	.40	.79	0.007 (-0.021 to 0.035)	.63	.75
Fmin	0.014 (-0.015 to 0.042)	.35	.56	-0.015 (-0.046 to 0.016)	.34	.79	-0.023 (-0.052 to 0.006)	.11	.22
CC	-0.011 (-0.038 to 0.017)	.45	.57	-0.003 (-0.034 to 0.027)	.84	.96	-0.003 (-0.031 to 0.025)	.83	.88
SLF (right)	-0.037 (-0.065 to -0.009)	.01	.06	-0.002 (-0.033 to 0.030)	.91	.96	0.050 (0.021 to 0.078)	.001	**.005**
SLF (left)	-0.066 (-0.094 to -0.039)	< .001	**< .001**	0.015 (-0.016 to 0.046)	.34	.79	0.050 (0.021 to 0.078)	.001	**.005**
tSLF (right)	-0.035 (-0.063 to -0.007)	.01	.07	-0.006 (-0.038 to 0.025)	.69	.93	0.042 (0.014 to 0.071)	.004	**.01**
tSLF (left)	-0.065 (-0.093 to -0.038)	< .001	**< .001**	0.019 (-0.012 to 0.050)	.22	.69	0.042 (0.014 to 0.071)	.003	**.01**
pSLF (right)	-0.037 (-0.064 to -0.009)	.01	.06	0.001 (-0.030 to 0.032)	.94	.96	0.050 (0.021 to 0.078)	.001	**.005**
pSLF (left)	-0.056 (-0.083 to -0.028)	< .001	**.001**	0.006 (-0.024 to 0.037)	.69	.93	0.058 (0.030 to 0.087)	< .001	**.002**
SCS (right)	0.020 (-0.007 to 0.047)	.15	.33	-0.015 (-0.045 to 0.016)	.34	.79	0.033 (0.004 to 0.061)	.02	.07
SCS (left)	0.006 (-0.012 to 0.044)	.27	.75	0.003 (-0.027 to 0.033)	.85	.96	0.039 (0.011 to 0.067)	.01	**.02**
SIFC (right)	0.016 (-0.012 to 0.044)	.27	.48	-0.032 (-0.063 to -0.002)	.04	.35	0.002 (-0.026 to 0.031)	.87	.88
SIFC (left)	-0.002 (-0.030 to 0.026)	.89	.92	-0.012 (-0.042 to 0.019)	.46	.79	-0.004 (-0.033 to 0.024)	.77	.86
IFSFC (right)	-0.023 (-0.050 to 0.005)	.10	.25	0.014 (-0.017 to 0.044)	.39	.79	0.022 (-0.007 to 0.050)	.13	.24
IFSFC (left)	-0.027 (-0.054 to 0.001)	.06	.20	0.012 (-0.019 to 0.043)	.45	.79	0.020 (-0.008 to 0.048)	.17	.24

© 2023 Li ZA et al. *JAMA Network Open*.

White matter tracts	SES indicators (IVs)								
	Neighborhood disadvantage (higher = lower SES)			Household income (higher = higher SES)			Parental education (higher = higher SES)		
	β **(95% CI)**	***p*-value** (nominal)	***p*-value** (FDR)	***β*** **(95% CI)**	***p*-value** (nominal)	***p*-value** (FDR)	***β*** **(95% CI)**	***p*-value** (nominal)	***p*-value** (FDR)
**D) DTI-MD (DV)**									
Fx (right)	0.003 (-0.024 to 0.030)	.82	.82	-0.027 (-0.058 to 0.004)	.09	.24	0.009 (-0.019 to 0.038)	.52	.76
Fx (left)	-0.003 (-0.031 to 0.024)	.81	.82	-0.010 (-0.041 to 0.021)	.54	.59	0.008 (-0.021 to 0.036)	.60	.78
CgC (right)	-0.013 (-0.041 to 0.014)	.35	.51	0.009 (-0.022 to 0.041)	.56	.59	0.011 (-0.017 to 0.040)	.44	.76
CgC (left)	-0.012 (-0.039 to 0.016)	.40	.54	0.020 (-0.011 to 0.050)	.20	.32	0.003 (-0.026 to 0.031)	.85	.91
CgH (right)	-0.016 (-0.044 to 0.012)	.25	.41	0.020 (-0.011 to 0.051)	.21	.32	-0.008 (-0.037 to 0.020)	.56	.76
CgH (left)	-0.020 (-0.047 to 0.007)	.15	.33	0.039 (-0.008 to 0.069)	.01	.13	-0.004 (-0.032 to 0.024)	.78	.91
CST (right)	-0.022 (-0.050 to 0.005)	.11	.32	0.035 (-0.004 to 0.066)	.03	.13	-0.007 (-0.035 to 0.022)	.65	.81
CST (left)	-0.033 (-0.061 to -0.004)	.02	.12	0.029 (-0.002 to 0.061)	.07	.23	0.002 (-0.026 to 0.031)	.88	.91
ATR (right)	-0.012 (-0.040 to 0.017)	.42	.54	0.040 (0.009 to 0.071)	.01	.13	-0.014 (-0.042 to 0.014)	.33	.73
ATR (left)	-0.008 (-0.035 to 0.019)	.56	.67	0.028 (-0.003 to 0.058)	.08	.23	0.004 (-0.024 to 0.032)	.79	.91
Unc (right)	0.016 (-0.012 to 0.043)	.27	.42	0.037 (0.005 to 0.068)	.02	.13	-0.009 (-0.037 to 0.020)	.54	.76
Unc (left)	0.004 (-0.024 to 0.032)	.78	.82	0.027 (-0.005 to 0.058)	.09	.24	0.001 (-0.027 to 0.030)	.92	.92
ILF (right)	0.022 (-0.005 to 0.049)	.10	.32	0.018 (-0.013 to 0.048)	.25	.33	-0.016 (-0.044 to 0.012)	.27	.73
ILF (left)	0.020 (-0.006 to 0.047)	.14	.32	0.009 (-0.022 to 0.040)	.57	.59	-0.016 (-0.044 to 0.012)	.26	.73
IFOF (right)	0.026 (-0.001 to 0.053)	.06	.20	0.036 (0.006 to 0.066)	.02	.13	-0.011 (-0.039 to 0.016)	.42	.76
IFOF (left)	0.019 (-0.008 to 0.046)	.17	.33	0.031 (0.001 to 0.062)	.04	.20	-0.009 (-0.037 to 0.018)	.50	.76
Fmaj	0.019 (-0.009 to 0.047)	.18	.33	-0.016 (-0.047 to 0.015)	.31	.37	0.039 (0.011 to 0.068)	.01	.23
Fmin	-0.004 (-0.032 to 0.024)	.79	.82	0.010 (-0.021 to 0.042)	.52	.59	0.031 (0.002 to 0.060)	.04	.51
CC	0.021 (-0.006 to 0.049)	.13	.32	0.006 (-0.025 to 0.037)	.71	.71	0.021 (-0.007 to 0.050)	.14	.71
SLF (right)	0.034 (0.007 to 0.061)	.01	.12	0.023 (-0.007 to 0.054)	.13	.30	-0.025 (-0.053 to 0.003)	.09	.65
SLF (left)	0.034 (0.006 to 0.062)	.02	.12	0.018 (-0.013 to 0.049)	.25	.33	-0.016 (-0.044 to 0.012)	.27	.73
tSLF (right)	0.029 (0.002 to 0.056)	.04	.16	0.029 (-0.002 to 0.060)	.06	.23	-0.028 (-0.056 to 0.000)	.05	.51
tSLF (left)	0.032 (0.004 to 0.060)	.02	.12	0.019 (-0.012 to 0.050)	.23	.33	-0.016 (-0.044 to 0.012)	.27	.73
pSLF (right)	0.035 (0.008 to 0.063)	.01	.12	0.023 (-0.008 to 0.054)	.15	.30	-0.023 (-0.051 to 0.005)	.11	.65
pSLF (left)	0.036 (0.008 to 0.064)	.01	.12	0.020 (-0.011 to 0.051)	.21	.32	-0.015 (-0.043 to 0.013)	.30	.73
SCS (right)	0.027 (0.000 to 0.055)	.05	.19	0.024 (-0.007 to 0.055)	.13	.30	-0.015 (-0.043 to 0.013)	.29	.73
SCS (left)	0.008 (-0.019 to 0.036)	.56	.67	0.019 (-0.011 to 0.050)	.22	.32	-0.010 (-0.038 to 0.019)	.50	.76
SIFC (right)	0.012 (-0.016 to 0.040)	.39	.54	0.037 (0.007 to 0.068)	.02	.13	-0.015 (-0.043 to 0.014)	.31	.73
SIFC (left)	-0.004 (-0.032 to 0.024)	.78	.82	0.020 (-0.011 to 0.051)	.21	.32	0.012 (-0.017 to 0.040)	.41	.76
IFSFC (right)	0.018 (-0.009 to 0.046)	.19	.33	0.018 (-0.013 to 0.049)	.26	.33	-0.012 (-0.041 to 0.016)	.39	.76
IFSFC (left)	0.019 (-0.008 to 0.047)	.17	.33	0.021 (-0.010 to 0.052)	.19	.32	-0.002 (-0.031 to 0.026)	.88	.91

Abbreviations: ATR, anterior thalamic radiations; CC, corpus callosum; CgC, cingulate cingulum; CgH, parahippocampal cingulum; CI, confidence interval; CST, corticospinal/pyramidal tract; DTI, diffusion tensor imaging; DV, dependent variable; FA, fractional anisotropy; FDR, false discovery rate; Fmaj, forceps major; Fmin, forceps minor; Fx, fornix; IFOF, inferior frontal-occipital fasciculus; IFSFC, inferior-frontal to

© 2023 Li ZA et al. *JAMA Network Open*.

superior-frontal cortical tract; ILF, inferior longitudinal fasciculus; IV, independent variable; MD, mean diffusivity; pSLF, parietal superior longitudinal fasciculus; RND, restricted normalized directional; RNI, restricted normalized isotropic; RSI, restriction spectrum imaging; SCS, superior-corticostriatal tract; SES, socioeconomic status; SIFC, striatal to inferior-frontal cortical tract; SLF, superior longitudinal fasciculus; tSLF, temporal superior longitudinal fasciculus; Unc, uncinate fasciculus

[a] Linear mixed effects models included all three SES indicators simultaneously as IVs and RSI and DTI metrics in each of the white matter tracts as the dependent variable DV. Models were adjusted for participant age, sex, pubertal development stage, intracranial volume, and mean head motion, and were nested by family. Multiple comparison correction was conducted within each neuroimaging metric and by each SES indicator, giving 31 tests to correct for in each group of models. Associations that survived FDR-corrected *p*-value $\leq .05$ were considered statistically significant and are highlighted. Estimates were standardized β's with 95% CIs.

© 2023 Li ZA et al. *JAMA Network Open*.

eFigure 5. Heat Map of Associations Between SES and White Matter Microstructure

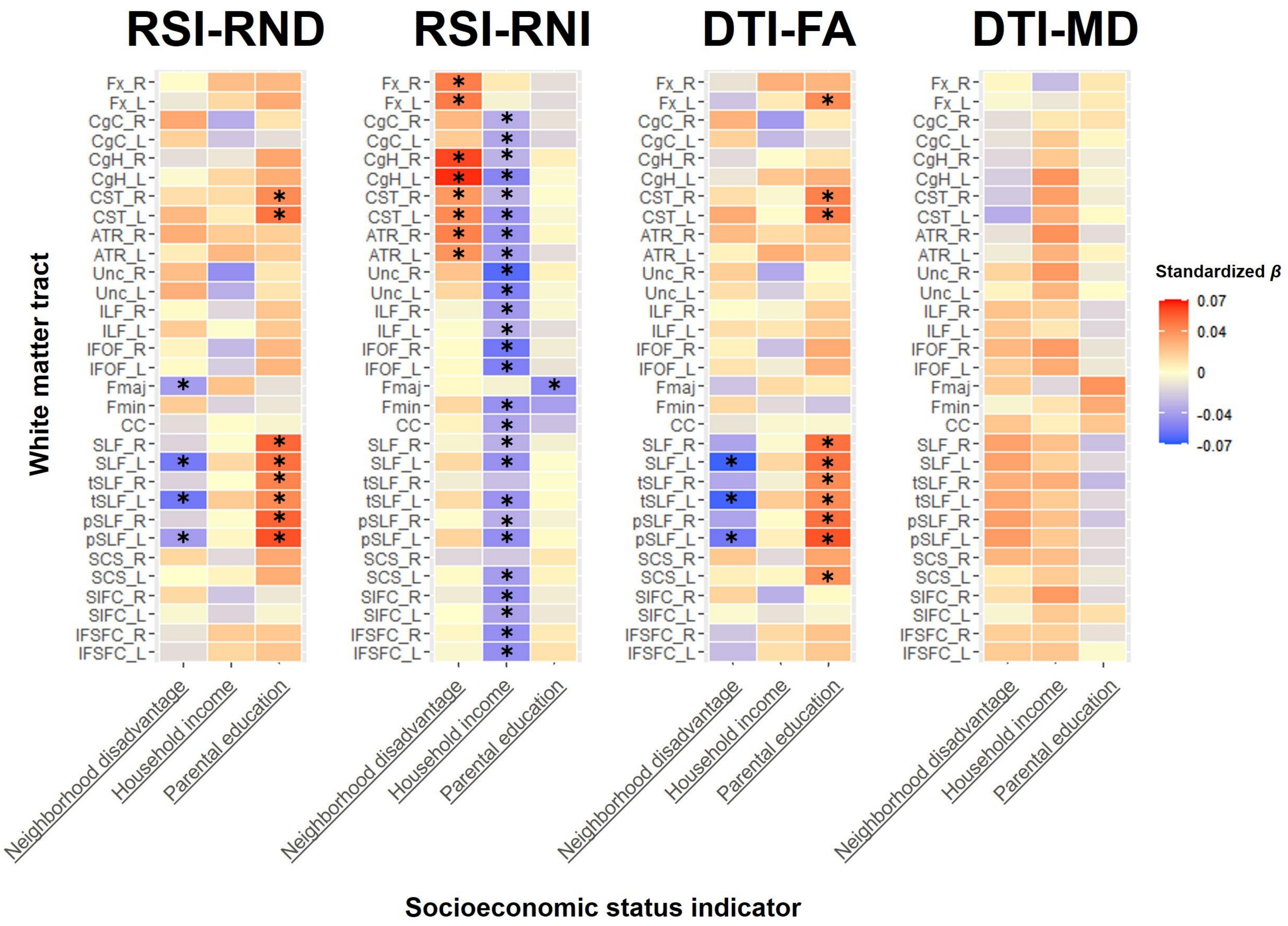

Note. Standardized β's of the independent associations between socioeconomic status indicators and RSI and DTI metrics in each of the white matter tracts are shown (full statistics in **eTable 9** in the **Supplement**). *, false discovery rate (FDR)-corrected *p*-value ≤ .05. Abbreviations: ATR, anterior thalamic radiations; CC, corpus callosum; CgC, cingulate cingulum; CgH, parahippocampal cingulum; CST, corticospinal/pyramidal tract; DTI, diffusion tensor imaging; FA, fractional anisotropy; Fmaj, forceps major; Fmin, forceps minor; Fx, fornix; IFOF, inferior frontal-occipital fasciculus; IFSFC, inferior-frontal to superior-frontal cortical tract; ILF, inferior longitudinal fasciculus; L, left; MD, mean diffusivity; pSLF, parietal superior longitudinal fasciculus; R, right; RND, restricted normalized directional; RNI, restricted normalized isotropic; RSI, restriction spectrum imaging; SCS, superior-corticostriatal tract; SES, socioeconomic status; SIFC, striatal to inferior-frontal cortical tract; SLF, superior longitudinal fasciculus; tSLF, temporal superior longitudinal fasciculus; Unc, uncinate fasciculus

© 2023 Li ZA et al. *JAMA Network Open*.

eTable 10. Sensitivity Analyses on Associations Between SES and White Matter Microstructure[a]

1) Using only scans with low head motion (mean motion ≤ 2.5 mm)

**White matter tracts**	**SES indicators (IVs)**						**Sample size (n)**
	**Neighborhood disadvantage** (higher = lower SES)		**Household income** (higher = higher SES)		**Parental education** (higher = higher SES)		
	**β (95% CI)**	***p*-value** (nominal)	**β (95% CI)**	***p*-value** (nominal)	**β (95% CI)**	***p*-value** (nominal)	
**A) RSI-RND (DV)**							
CST (right)	0.010 (-0.017 to 0.037)	.46	0.014 (-0.016 to 0.044)	.36	0.041 (0.013 to 0.069)	.005	8618
CST (left)	0.026 (-0.002 to 0.053)	.06	0.008 (-0.022 to 0.039)	.60	0.046 (0.018 to 0.075)	.001	8616
Fmaj	-0.043 (-0.071 to -0.015)	.003	0.022 (-0.009 to 0.054)	.16	-0.012 (-0.041 to 0.017)	.42	8612
SLF (right)	-0.021 (-0.049 to 0.006)	.13	-0.003 (-0.034 to 0.029)	.87	0.053 (0.024 to 0.082)	< .001	8623
SLF (left)	-0.064 (-0.091 to -0.036)	< .001	0.013 (-0.018 to 0.044)	.41	0.048 (0.020 to 0.077)	.001	8619
tSLF (right)	-0.021 (-0.049 to 0.006)	.13	-0.003 (-0.034 to 0.028)	.85	0.045 (0.016 to 0.074)	.002	8624
tSLF (left)	-0.065 (-0.093 to -0.038)	< .001	0.018 (-0.013 to 0.049)	.26	0.040 (0.011 to 0.068)	.01	8622
pSLF (right)	-0.023 (-0.050 to 0.005)	.10	-0.003 (-0.034 to 0.029)	.87	0.054 (0.025 to 0.082)	< .001	8621
pSLF (left)	-0.048 (-0.075 to -0.020)	.001	0.001 (-0.030 to 0.032)	.96	0.058 (0.030 to 0.087)	< .001	8614
**B) RSI-RNI (DV)**							
Fx (right)	0.047 (0.020 to 0.075)	.001	0.010 (-0.021 to 0.041)	.53	-0.016 (-0.045 to 0.013)	.29	8601
Fx (left)	0.049 (0.021 to 0.076)	.001	-0.001 (-0.033 to 0.030)	.93	-0.016 (-0.045 to 0.013)	.29	8584
CgC (right)	0.027 (0.000 to 0.054)	.05	-0.034 (-0.065 to -0.004)	.03	-0.014 (-0.042 to 0.015)	.35	8605
CgC (left)	0.018 (-0.010 to 0.045)	.21	-0.034 (-0.065 to -0.003)	.03	-0.019 (-0.048 to 0.010)	.20	8594
CgH (right)	0.061 (0.033 to 0.088)	< .001	-0.032 (-0.062 to -0.001)	.03	0.004 (-0.024 to 0.032)	.78	8608
CgH (left)	0.065 (0.038 to 0.093)	< .001	-0.051 (-0.082 to -0.021)	.001	-0.005 (-0.034 to 0.023)	.71	8590
CST (right)	0.038 (0.010 to 0.065)	.01	-0.032 (-0.063 to -0.001)	.05	-0.002 (-0.031 to 0.026)	.87	8615
CST (left)	0.041 (0.014 to 0.068)	.003	-0.045 (-0.076 to -0.013)	.01	-0.004 (-0.032 to 0.025)	.79	8607
ATR (right)	0.047 (0.020 to 0.074)	.001	-0.046 (-0.077 to -0.015)	.003	0.004 (-0.024 to 0.033)	.76	8579
ATR (left)	0.040 (0.012 to 0.067)	.005	-0.038 (-0.070 to -0.007)	.02	-0.013 (-0.041 to 0.016)	.38	8576
Unc (right)	0.021 (-0.006 to 0.049)	.12	-0.062 (-0.093 to -0.032)	< .001	0.002 (-0.026 to 0.031)	.87	8616
Unc (left)	0.012 (-0.015 to 0.040)	.38	-0.056 (-0.087 to -0.025)	< .001	-0.005 (-0.034 to 0.024)	.74	8615
ILF (right)	-0.007 (-0.034 to 0.019)	.59	-0.044 (-0.075 to -0.014)	.005	-0.004 (-0.032 to 0.024)	.79	8623
ILF (left)	-0.005 (-0.032 to 0.022)	.74	-0.034 (-0.065 to -0.003)	.03	-0.015 (-0.043 to 0.013)	.30	8618
IFOF (right)	0.001 (-0.025 to 0.028)	.92	-0.055 (-0.085 to -0.025)	< .001	-0.009 (-0.037 to 0.019)	.52	8619
IFOF (left)	0.000 (-0.026 to 0.027)	.97	-0.051 (-0.082 to -0.020)	.001	-0.014 (-0.042 to 0.014)	.33	8591
Fmaj	0.001 (-0.027 to 0.028)	.96	-0.005 (-0.036 to 0.026)	.74	-0.051 (-0.080 to -0.023)	< .001	8588
Fmin	0.013 (-0.014 to 0.041)	.34	-0.045 (-0.076 to 0.013)	.01	-0.039 (-0.068 to -0.010)	.01	8608
CC	0.002 (-0.025 to 0.030)	.87	-0.037 (-0.068 to -0.005)	.02	-0.027 (-0.055 to 0.002)	.07	8609
SLF (right)	-0.005 (-0.031 to 0.022)	.73	-0.031 (-0.061 to -0.001)	.05	-0.007 (-0.035 to 0.021)	.63	8624

© 2023 Li ZA et al. *JAMA Network Open*.

SLF (left)	0.011 (-0.016 to 0.038)	.42	-0.046 (-0.077 to -0.016)	.003	-0.003 (-0.031 to 0.025)	.83	8613
tSLF (left)	0.010 (-0.017 to 0.037)	.46	-0.045 (-0.076 to -0.015)	.004	-0.001 (-0.029 to 0.028)	.97	8610
pSLF (right)	-0.003 (-0.029 to 0.024)	.85	-0.033 (-0.065 to -0.003)	.03	-0.006 (-0.034 to 0.022)	.67	8625
pSLF (left)	0.011 (-0.016 to 0.038)	.42	-0.047 (-0.078 to -0.017)	.003	-0.001 (-0.030 to 0.027)	.93	8617
SCS (left)	0.001 (-0.025 to 0.028)	.93	-0.038 (-0.069 to -0.008)	.01	0.003 (-0.025 to 0.031)	.83	8614
SIFC (right)	-0.006 (-0.033 to 0.022)	.69	-0.043 (-0.074 to -0.012)	.01	-0.008 (-0.037 to 0.021)	.58	8618
SIFC (left)	0.001 (-0.027 to 0.028)	.96	-0.038 (-0.069 to -0.006)	.02	-0.012 (-0.040 to 0.017)	.43	8619
IFSFC (right)	0.001 (-0.026 to 0.027)	.97	-0.047 (-0.078 to -0.016)	.003	0.006 (-0.022 to 0.035)	.66	8622
IFSFC (left)	-0.006 (-0.033 to 0.021)	.66	-0.047 (-0.078 to -0.016)	.003	0.007 (-0.021 to 0.036)	.61	8624
**C) DTI-FA (DV)**							
Fx (left)	-0.028 (-0.055 to 0.000)	.05	0.013 (-0.018 to 0.044)	.42	0.037 (0.008 to 0.066)	.01	8630
CST (right)	0.010 (-0.017 to 0.037)	.49	-0.003 (-0.033 to 0.028)	.85	0.045 (0.017 to 0.074)	.002	8626
CST (left)	0.026 (-0.001 to 0.053)	.06	-0.002 (-0.032 to 0.029)	.92	0.044 (0.015 to 0.072)	.003	8627
SLF (right)	-0.042 (-0.070 to -0.014)	.004	-0.004 (-0.036 to 0.028)	.80	0.051 (0.022 to 0.080)	.001	8628
SLF (left)	-0.074 (-0.102 to -0.046)	< .001	0.014 (-0.017 to 0.045)	.38	0.048 (0.019 to 0.077)	.001	8627
tSLF (right)	-0.038 (-0.066 to -0.010)	.01	-0.009 (-0.041 to 0.023)	.58	0.043 (0.014 to 0.072)	.004	8629
tSLF (left)	-0.072 (-0.100 to -0.044)	< .001	0.019 (-0.013 to 0.050)	.24	0.040 (0.012 to 0.069)	.01	8628
pSLF (right)	-0.042 (-0.070 to -0.014)	.004	-0.001 (-0.033 to 0.031)	.95	0.050 (0.021 to 0.079)	.001	8628
pSLF (left)	-0.063 (-0.091 to -0.035)	< .001	0.005 (-0.027 to 0.036)	.77	0.056 (0.028 to 0.085)	< .001	8624
SCS (left)	0.002 (-0.025 to 0.029)	.90	0.001 (-0.029 to 0.032)	.93	0.032 (0.004 to 0.060)	.03	8629

2) Using only participants without reported adverse childhood experiences

**White matter tracts**	**SES indicators (IVs)**						**Sample size (*n*)**
	**Neighborhood disadvantage** (higher = lower SES)		**Household income** (higher = higher SES)		**Parental education** (higher = higher SES)		
	***β* (95% CI)**	***p*-value** (nominal)	***β* (95% CI)**	***p*-value** (nominal)	***β* (95% CI)**	***p*-value** (nominal)	
**A) RSI-RND (DV)**							
CST (right)	-0.002 (-0.035 to 0.031)	.90	-0.001 (-0.039 to 0.036)	.95	0.034 (-0.001 to 0.068)	.06	5670
CST (left)	0.019 (-0.015 to 0.054)	.26	0.002 (-0.036 to 0.040)	.92	0.037 (0.003 to 0.072)	.04	5668
Fmaj	-0.023 (-0.057 to 0.012)	.19	0.032 (-0.007 to 0.070)	.11	-0.003 (-0.039 to 0.032)	.85	5669
SLF (right)	-0.010 (-0.044 to 0.023)	.54	-0.010 (-0.048 to 0.027)	.59	0.062 (0.027 to 0.097)	< .001	5674
SLF (left)	-0.051 (-0.084 to -0.018)	.003	0.011 (-0.026 to 0.049)	.55	0.053 (0.018 to 0.087)	.003	5671
tSLF (right)	-0.017 (-0.051 to 0.016)	.31	-0.016 (-0.054 to 0.022)	.40	0.056 (0.020 to 0.091)	.002	5675
tSLF (left)	-0.053 (-0.086 to -0.019)	.002	0.014 (-0.023 to 0.052)	.46	0.045 (0.010 to 0.079)	.01	5673
pSLF (right)	-0.010 (-0.043 to 0.023)	.54	-0.008 (-0.046 to 0.029)	.66	0.063 (0.028 to 0.097)	< .001	5674
pSLF (left)	-0.035 (-0.068 to -0.002)	.04	0.002 (-0.035 to 0.039)	.91	0.062 (0.028 to 0.096)	< .001	5669
**B) RSI-RNI (DV)**							

© 2023 Li ZA et al. *JAMA Network Open*.

Fx (right)	0.051 (0.016 to 0.085)	.004	0.003 (-0.036 to 0.042)	.88	-0.004 (-0.040 to 0.032)	.81	5663
Fx (left)	0.048 (0.013 to 0.082)	.01	-0.015 (-0.054 to 0.024)	.46	-0.007 (-0.043 to 0.029)	.69	5654
CgC (right)	0.046 (0.012 to 0.080)	.01	-0.028 (-0.066 to 0.010)	.14	-0.010 (-0.045 to 0.026)	.59	5669
CgC (left)	0.035 (0.001 to 0.069)	.05	-0.025 (-0.064 to 0.014)	.21	-0.010 (-0.046 to 0.026)	.60	5661
CgH (right)	0.077 (0.043 to 0.110)	< .001	-0.012 (-0.050 to 0.025)	.52	0.003 (-0.033 to 0.038)	.88	5669
CgH (left)	0.070 (0.036 to 0.104)	< .001	-0.029 (-0.066 to 0.009)	.14	-0.014 (-0.049 to 0.021)	.43	5654
CST (right)	0.057 (0.023 to 0.091)	.001	-0.017 (-0.055 to 0.021)	.38	0.012 (-0.024 to 0.047)	.51	5672
CST (left)	0.056 (0.022 to 0.090)	.001	-0.033 (-0.071 to 0.005)	.09	0.009 (-0.026 to 0.044)	.61	5670
ATR (right)	0.052 (0.018 to 0.085)	.003	-0.051 (-0.089 to -0.013)	.01	0.010 (-0.025 to 0.046)	.57	5647
ATR (left)	0.051 (0.017 to 0.085)	.003	-0.042 (-0.080 to -0.003)	.03	-0.011 (-0.047 to 0.024)	.53	5648
Unc (right)	0.030 (-0.003 to 0.064)	.08	-0.046 (-0.085 to -0.008)	.02	-0.003 (-0.038 to 0.033)	.89	5672
Unc (left)	0.028 (-0.025 to 0.062)	.10	-0.037 (-0.076 to 0.001)	.06	-0.013 (-0.049 to 0.022)	.46	5670
ILF (right)	0.006 (-0.028 to 0.039)	.74	-0.031 (-0.069 to 0.008)	.12	-0.007 (-0.042 to 0.028)	.69	5677
ILF (left)	0.011 (-0.022 to 0.044)	.51	-0.021 (-0.060 to 0.017)	.28	-0.019 (-0.054 to 0.017)	.30	5673
IFOF (right)	0.001 (-0.032 to 0.034)	.95	-0.055 (-0.093 to -0.017)	.004	-0.010 (-0.045 to 0.025)	.59	5675
IFOF (left)	0.010 (-0.023 to 0.043)	.56	-0.049 (-0.087 to -0.011)	.01	-0.013 (-0.049 to 0.022)	.46	5655
Fmaj	0.008 (-0.027 to 0.042)	.67	-0.011 (-0.050 to 0.027)	.57	-0.040 (-0.075 to -0.004)	.03	5653
Fmin	0.028 (-0.006 to 0.063)	.10	-0.046 (-0.085 to -0.007)	.02	-0.040 (-0.076 to -0.004)	.03	5666
CC	0.017 (-0.017 to 0.051)	.32	-0.036 (-0.075 to 0.003)	.07	-0.025 (-0.061 to 0.011)	.17	5666
SLF (right)	0.002 (-0.031 to 0.035)	.89	-0.021 (-0.058 to 0.017)	.28	-0.014 (-0.049 to 0.020)	.42	5676
SLF (left)	0.028 (-0.006 to 0.063)	.10	-0.032 (-0.070 to 0.006)	.10	-0.004 (-0.039 to 0.031)	.81	5671
tSLF (left)	0.026 (-0.007 to 0.060)	.12	-0.029 (-0.067 to 0.009)	.13	-0.002 (-0.037 to 0.033)	.93	5670
pSLF (right)	0.004 (-0.029 to 0.037)	.79	-0.024 (-0.062 to 0.013)	.20	-0.015 (-0.050 to 0.020)	.40	5677
pSLF (left)	0.028 (-0.006 to 0.061)	.10	-0.037 (-0.075 to 0.001)	.06	-0.004 (-0.039 to 0.031)	.83	5673
SCS (left)	0.011 (-0.022 to 0.044)	.51	-0.027 (-0.065 to 0.010)	.15	-0.001 (-0.035 to 0.034)	.97	5669
SIFC (right)	0.000 (-0.034 to 0.034)	.99	-0.034 (-0.072 to 0.004)	.08	-0.016 (-0.052 to 0.020)	.38	5672
SIFC (left)	0.020 (-0.014 to 0.053)	.25	-0.023 (-0.061 to 0.016)	.25	-0.019 (-0.055 to 0.016)	.29	5672
IFSFC (right)	0.013 (-0.020 to 0.046)	.44	-0.036 (-0.074 to 0.002)	.06	0.004 (-0.031 to 0.039)	.83	5675
IFSFC (left)	0.014 (-0.019 to 0.048)	.40	-0.036 (-0.074 to 0.002)	.06	0.007 (-0.028 to 0.043)	.68	5676
**C) DTI-FA (DV)**							
Fx (left)	-0.011 (-0.046 to 0.023)	.51	-0.003 (-0.041 to 0.036)	.89	0.055 (0.019 to 0.090)	.003	5681
CST (right)	0.002 (-0.032 to 0.035)	.92	-0.014 (-0.052 to 0.024)	.47	0.041 (0.006 to 0.076)	.02	5679
CST (left)	0.029 (-0.005 to 0.064)	.10	-0.004 (-0.043 to 0.034)	.83	0.040 (0.005 to 0.075)	.02	5678
SLF (right)	-0.033 (-0.067 to 0.002)	.06	-0.006 (-0.045 to 0.033)	.77	0.059 (0.023 to 0.095)	.001	5680
SLF (left)	-0.064 (-0.099 to -0.030)	< .001	0.016 (-0.023 to 0.055)	.42	0.047 (0.012 to 0.082)	.01	5680
tSLF (right)	-0.035 (-0.069 to -0.001)	.05	-0.018 (-0.057 to 0.021)	.36	0.051 (0.016 to 0.087)	.005	5680
tSLF (left)	-0.064 (-0.099 to -0.030)	< .001	0.017 (-0.022 to 0.055)	.40	0.040 (0.005 to 0.075)	.03	5681
pSLF (right)	-0.031 (-0.065 to 0.003)	.08	0.000 (-0.038 to 0.039)	.98	0.059 (0.023 to 0.094)	.001	5680
pSLF (left)	-0.052 (-0.086 to -0.017)	.003	0.013 (-0.025 to 0.052)	.50	0.054 (0.019 to 0.090)	.003	5680
SCS (left)	0.019 (-0.015 to 0.053)	.28	0.009 (-0.029 to 0.047)	.65	0.028 (-0.007 to 0.063)	.12	5680

© 2023 Li ZA et al. *JAMA Network Open*.

3) Using only participants without common psychiatric diagnoses

White matter tracts	SES indicators (IVs)						Sample size (*n*)
	Neighborhood disadvantage (higher = lower SES)		Household income (higher = higher SES)		Parental education (higher = higher SES)		
	β (95% CI)	*p*-value (nominal)	*β* (95% CI)	*p*-value (nominal)	*β* (95% CI)	*p*-value (nominal)	
**A) RSI-RND (DV)**							
CST (right)	0.006 (-0.022 to 0.034)	.66	0.010 (-0.021 to 0.042)	.52	0.040 (0.010 to 0.069)	.01	7672
CST (left)	0.024 (-0.005 to 0.052)	.10	0.008 (-0.024 to 0.040)	.61	0.045 (0.015 to 0.074)	.003	7672
Fmaj	-0.041 (-0.070 to -0.012)	.01	0.026 (-0.006 to 0.059)	.11	-0.011 (-0.041 to 0.019)	.49	7669
SLF (right)	-0.021 (-0.050 to 0.008)	.15	-0.003 (-0.035 to 0.029)	.85	0.056 (0.026 to 0.086)	< .001	7677
SLF (left)	-0.056 (-0.084 to -0.028)	< .001	0.012 (-0.019 to 0.044)	.45	0.052 (0.022 to 0.081)	.001	7671
tSLF (right)	-0.018 (-0.047 to 0.011)	.21	-0.004 (-0.036 to 0.028)	.81	0.053 (0.023 to 0.083)	.001	7676
tSLF (left)	-0.055 (-0.083 to -0.026)	< .001	0.017 (-0.015 to 0.049)	.30	0.046 (0.016 to 0.075)	.002	7673
pSLF (right)	-0.023 (-0.052 to 0.005)	.11	-0.002 (-0.034 to 0.030)	.90	0.055 (0.026 to 0.084)	< .001	7673
pSLF (left)	-0.044 (-0.072 to -0.016)	.002	0.000 (-0.031 to 0.031)	> .99	0.059 (0.029 to 0.088)	< .001	7667
**B) RSI-RNI (DV)**							
Fx (right)	0.054 (0.025 to 0.083)	< .001	0.005 (-0.028 to 0.038)	.75	-0.001 (-0.031 to 0.030)	.96	7661
Fx (left)	0.051 (0.022 to 0.080)	.001	-0.004 (-0.038 to 0.029)	.79	-0.007 (-0.037 to 0.024)	.67	7643
CgC (right)	0.032 (0.003 to 0.061)	.03	-0.036 (-0.069 to -0.004)	.03	-0.013 (-0.043 to 0.017)	.39	7664
CgC (left)	0.022 (-0.007 to 0.051)	.14	-0.032 (-0.065 to 0.000)	.05	-0.025 (-0.055 to 0.006)	.11	7653
CgH (right)	0.061 (0.033 to 0.090)	< .001	-0.035 (-0.067 to -0.003)	.03	0.008 (-0.022 to 0.038)	.61	7666
CgH (left)	0.068 (0.040 to 0.097)	< .001	-0.052 (-0.084 to -0.019)	.002	-0.003 (-0.032 to 0.027)	.87	7648
CST (right)	0.040 (0.011 to 0.069)	.01	-0.027 (-0.060 to 0.006)	.11	0.001 (-0.029 to 0.031)	.94	7673
CST (left)	0.048 (0.019 to 0.076)	.001	-0.041 (-0.073 to -0.008)	.02	0.004 (-0.026 to 0.034)	.79	7665
ATR (right)	0.048 (0.019 to 0.077)	.001	-0.051 (-0.083 to -0.018)	.002	0.009 (-0.021 to 0.039)	.55	7639
ATR (left)	0.043 (0.014 to 0.072)	.003	-0.040 (-0.073 to -0.007)	.02	-0.010 (-0.041 to 0.020)	.50	7640
Unc (right)	0.027 (-0.002 to 0.056)	.07	-0.064 (-0.097 to -0.032)	< .001	0.010 (-0.020 to 0.040)	.53	7674
Unc (left)	0.018 (-0.011 to 0.047)	.22	-0.051 (-0.083 to -0.018)	.003	-0.002 (-0.032 to 0.029)	.91	7674
ILF (right)	-0.003 (-0.031 to 0.026)	.84	-0.042 (-0.074 to -0.009)	.01	-0.002 (-0.032 to 0.028)	.91	7680
ILF (left)	-0.001 (-0.030 to 0.027)	.93	-0.025 (-0.058 to 0.007)	.13	-0.015 (-0.045 to 0.015)	.32	7678
IFOF (right)	0.005 (-0.023 to 0.034)	.71	-0.055 (-0.087 to -0.023)	.001	-0.003 (-0.032 to 0.027)	.86	7677
IFOF (left)	0.006 (-0.022 to 0.034)	.68	-0.047 (-0.080 to -0.015)	.004	-0.008 (-0.038 to 0.022)	.60	7652
Fmaj	0.006 (-0.023 to 0.036)	.66	0.000 (-0.033 to 0.033)	.99	-0.049 (-0.079 to -0.019)	.001	7647
Fmin	0.023 (-0.006 to 0.052)	.12	-0.038 (-0.071 to -0.005)	.02	-0.037 (-0.068 to -0.007)	.02	7670
CC	0.007 (-0.022 to 0.036)	.64	-0.029 (-0.062 to 0.004)	.09	-0.028 (-0.058 to 0.002)	.07	7668
SLF (right)	-0.001 (-0.029 to 0.027)	.95	-0.029 (-0.061 to 0.002)	.07	-0.002 (-0.032 to 0.027)	.89	7682
SLF (left)	0.020 (-0.009 to 0.048)	.18	-0.036 (-0.068 to -0.003)	.03	0.001 (-0.029 to 0.031)	.95	7672
tSLF (left)	0.017 (-0.012 to 0.045)	.25	-0.035 (-0.068 to -0.003)	.03	0.003 (-0.026 to 0.033)	.83	7668

© 2023 Li ZA et al. *JAMA Network Open*.

pSLF (right)	0.002 (-0.026 to 0.030)	.91	-0.031 (-0.062 to 0.001)	.06	-0.002 (-0.032 to 0.027)	.88	7683
pSLF (left)	0.021 (-0.007 to 0.050)	.15	-0.037 (-0.070 to -0.005)	.03	0.001 (-0.029 to 0.031)	.94	7674
SCS (left)	0.008 (-0.019 to 0.036)	.56	-0.034 (-0.066 to -0.002)	.04	0.007 (-0.022 to 0.036)	.64	7673
SIFC (right)	0.001 (-0.028 to 0.030)	.94	-0.046 (-0.078 to -0.013)	.01	-0.003 (-0.033 to 0.027)	.84	7676
SIFC (left)	0.008 (-0.020 to 0.037)	.57	-0.035 (-0.068 to -0.002)	.04	-0.007 (-0.038 to 0.023)	.63	7677
IFSFC (right)	0.007 (-0.021 to 0.035)	.62	-0.040 (-0.073 to -0.008)	.02	0.010 (-0.020 to 0.040)	.51	7680
IFSFC (left)	0.001 (-0.028 to 0.029)	.97	-0.038 (-0.071 to -0.005)	.02	0.013 (-0.017 to 0.043)	.40	7681
**C) DTI-FA (DV)**							
Fx (left)	-0.020 (-0.049 to 0.008)	.16	0.008 (-0.025 to 0.040)	.64	0.049 (0.018 to 0.079)	.002	7687
CST (right)	0.010 (-0.018 to 0.038)	.49	-0.007 (-0.039 to 0.025)	.67	0.043 (0.013 to 0.073)	.004	7683
CST (left)	0.032 (0.003 to 0.061)	.03	0.001 (-0.031 to 0.033)	.96	0.041 (0.011 to 0.071)	.01	7684
SLF (right)	-0.040 (-0.069 to -0.010)	.01	-0.007 (-0.040 to 0.026)	.69	0.057 (0.027 to 0.087)	< .001	7685
SLF (left)	-0.063 (-0.092 to -0.034)	< .001	0.012 (-0.021 to 0.044)	.49	0.053 (0.023 to 0.084)	.001	7684
tSLF (right)	-0.034 (-0.064 to -0.005)	.02	-0.013 (-0.046 to 0.020)	.45	0.054 (0.024 to 0.084)	< .001	7686
tSLF (left)	-0.060 (-0.089 to -0.031)	< .001	0.016 (-0.016 to 0.049)	.33	0.047 (0.016 to 0.077)	.002	7685
pSLF (right)	-0.040 (-0.070 to -0.011)	.01	-0.033 (-0.036 to 0.030)	.88	0.054 (0.024 to 0.085)	< .001	7685
pSLF (left)	-0.056 (-0.085 to -0.027)	< .001	0.002 (-0.031 to 0.034)	.93	0.061 (0.031 to 0.091)	< .001	7682
SCS (left)	0.009 (-0.020 to 0.038)	.55	-0.004 (-0.036 to 0.028)	.79	0.046 (0.016 to 0.076)	.002	7686

4) Using only participants with full-term birth

**White matter tracts**	**SES indicators (IVs)**						**Sample size (*n*)**
	**Neighborhood disadvantage** (higher = lower SES)		**Household income** (higher = higher SES)		**Parental education** (higher = higher SES)		
	***β* (95% CI)**	***p*-value** (nominal)	***β* (95% CI)**	***p*-value** (nominal)	***β* (95% CI)**	***p*-value** (nominal)	
**A) RSI-RND (DV)**							
CST (right)	0.012 (-0.017 to 0.041)	.43	0.006 (-0.027 to 0.039)	.71	0.051 (0.021 to 0.082)	.001	7147
CST (left)	0.025 (-0.005 to 0.055)	.10	0.008 (-0.026 to 0.041)	.64	0.055 (0.024 to 0.086)	.001	7145
Fmaj	-0.050 (-0.080 to -0.019)	.001	0.015 (-0.019 to 0.049)	.39	-0.014 (-0.045 to 0.018)	.40	7144
SLF (right)	-0.025 (-0.055 to 0.004)	.10	-0.004 (-0.038 to 0.029)	.80	0.058 (0.027 to 0.088)	< .001	7150
SLF (left)	-0.064 (-0.094 to -0.035)	< .001	0.010 (-0.023 to 0.043)	.56	0.054 (0.023 to 0.085)	.001	7143
tSLF (right)	-0.025 (-0.055 to 0.005)	.11	-0.002 (-0.036 to 0.032)	.92	0.051 (0.019 to 0.082)	.002	7149
tSLF (left)	-0.066 (-0.095 to -0.036)	< .001	0.014 (-0.019 to 0.048)	.39	0.045 (0.014 to 0.075)	.004	7144
pSLF (right)	-0.026 (-0.055 to 0.004)	.09	-0.006 (-0.039 to 0.028)	.74	0.059 (0.028 to 0.090)	< .001	7148
pSLF (left)	-0.045 (-0.074 to -0.016)	.002	0.000 (-0.032 to 0.033)	.98	0.068 (0.037 to 0.098)	< .001	7142
**B) RSI-RNI (DV)**							
Fx (right)	0.057 (0.027 to 0.087)	< .001	0.004 (-0.030 to 0.039)	.81	-0.010 (-0.042 to 0.022)	.54	7133
Fx (left)	0.059 (0.029 to 0.089)	< .001	-0.019 (-0.053 to 0.016)	.28	-0.001 (-0.033 to 0.031)	.94	7118

© 2023 Li ZA et al. *JAMA Network Open*.

CgC (right)	0.029 (0.000 to 0.059)	.05	-0.031 (-0.065 to 0.003)	.07	-0.015 (-0.046 to 0.017)	.36	7141
CgC (left)	0.027 (-0.004 to 0.057)	.08	-0.039 (-0.073 to -0.004)	.03	-0.019 (-0.051 to 0.013)	.24	7133
CgH (right)	0.066 (0.036 to 0.096)	< .001	-0.020 (-0.053 to 0.014)	.26	0.006 (-0.026 to 0.037)	.71	7139
CgH (left)	0.076 (0.046 to 0.106)	< .001	-0.036 (-0.070 to -0.002)	.04	-0.007 (-0.039 to 0.024)	.65	7127
CST (right)	0.053 (0.023 to 0.083)	.001	-0.025 (-0.059 to 0.009)	.15	-0.001 (-0.032 to 0.031)	.97	7145
CST (left)	0.055 (0.025 to 0.085)	< .001	-0.041 (-0.075 to -0.007)	.02	-0.001 (-0.033 to 0.030)	.94	7138
ATR (right)	0.053 (0.023 to 0.083)	.001	-0.041 (-0.075 to -0.007)	.02	0.000 (-0.032 to 0.031)	.98	7115
ATR (left)	0.054 (0.023 to 0.084)	.001	-0.042 (-0.077 to -0.008)	.02	-0.012 (-0.043 to 0.020)	.47	7117
Unc (right)	0.028 (-0.002 to 0.058)	.07	-0.052 (-0.086 to -0.018)	.003	-0.001 (-0.033 to 0.031)	.95	7141
Unc (left)	0.019 (-0.011 to 0.050)	.20	-0.047 (-0.081 to -0.013)	.01	-0.006 (-0.038 to 0.025)	.69	7143
ILF (right)	0.005 (-0.025 to 0.034)	.75	-0.035 (-0.069 to -0.002)	.04	0.000 (-0.031 to 0.031)	> .99	7152
ILF (left)	0.005 (-0.025 to 0.035)	.73	-0.035 (-0.070 to -0.001)	.04	-0.012 (-0.044 to 0.019)	.44	7145
IFOF (right)	0.008 (-0.022 to 0.037)	.61	-0.052 (-0.086 to -0.019)	.002	-0.005 (-0.036 to 0.026)	.75	7148
IFOF (left)	0.008 (-0.022 to 0.037)	.61	-0.056 (-0.089 to -0.022)	.001	-0.010 (-0.041 to 0.022)	.55	7131
Fmaj	0.014 (-0.017 to 0.044)	.37	-0.007 (-0.041 to 0.027)	.69	-0.044 (-0.076 to -0.012)	.01	7124
Fmin	0.021 (-0.010 to 0.051)	.19	-0.042 (-0.077 to -0.008)	.02	-0.047 (-0.079 to -0.015)	.004	7138
CC	0.014 (-0.016 to 0.044)	.36	-0.038 (-0.072 to -0.003)	.03	-0.022 (-0.054 to 0.010)	.18	7138
SLF (right)	0.000 (-0.030 to 0.029)	.98	-0.031 (-0.064 to 0.002)	.06	-0.007 (-0.038 to 0.024)	.67	7152
SLF (left)	0.021 (-0.008 to 0.051)	.16	-0.051 (-0.084 to -0.017)	.003	0.002 (-0.029 to 0.033)	.89	7144
tSLF (left)	0.020 (-0.010 to 0.050)	.19	-0.051 (-0.084 to -0.018)	.003	0.007 (-0.025 to 0.038)	.68	7142
pSLF (right)	0.002 (-0.028 to 0.031)	.91	-0.032 (-0.065 to 0.001)	.06	-0.007 (-0.038 to 0.024)	.65	7153
pSLF (left)	0.023 (-0.007 to 0.053)	.13	-0.048 (-0.081 to -0.015)	.005	0.002 (-0.029 to 0.034)	.89	7145
SCS (left)	0.009 (-0.020 to 0.038)	.55	-0.046 (-0.079 to -0.013)	.01	0.010 (-0.021 to 0.041)	.51	7144
SIFC (right)	-0.003 (-0.033 to 0.028)	.87	-0.041 (-0.075 to -0.007)	.02	-0.012 (-0.044 to 0.020)	.45	7145
SIFC (left)	0.003 (-0.026 to 0.033)	.82	-0.034 (-0.069 to 0.000)	.05	-0.014 (-0.045 to 0.018)	.40	7146
IFSFC (right)	0.010 (-0.019 to 0.040)	.49	-0.047 (-0.081 to -0.014)	.01	0.012 (-0.020 to 0.043)	.47	7150
IFSFC (left)	0.003 (-0.027 to 0.033)	.84	-0.046 (-0.079 to -0.012)	.01	0.012 (-0.020 to 0.043)	.47	7149
**C) DTI-FA (DV)**							
Fx (left)	-0.029 (-0.059 to 0.001)	.06	0.007 (-0.027 to 0.041)	.69	0.035 (0.003 to 0.067)	.03	7156
CST (right)	0.015 (-0.015 to 0.044)	.33	-0.006 (-0.039 to 0.028)	.74	0.055 (0.024 to 0.086)	.001	7155
CST (left)	0.032 (0.002 to 0.062)	.04	-0.005 (-0.039 to 0.028)	.75	0.056 (0.024 to 0.087)	< .001	7154
SLF (right)	-0.040 (-0.071 to -0.009)	.01	-0.002 (-0.036 to 0.032)	.90	0.060 (0.028 to 0.092)	< .001	7155
SLF (left)	-0.070 (-0.101 to -0.040)	< .001	0.017 (-0.017 to 0.051)	.33	0.052 (0.020 to 0.083)	.001	7154
tSLF (right)	-0.035 (-0.066 to -0.005)	.02	-0.004 (-0.038 to 0.031)	.83	0.052 (0.020 to 0.084)	.001	7155
tSLF (left)	-0.069 (-0.099 to -0.039)	< .001	0.020 (-0.014 to 0.054)	.24	0.043 (0.011 to 0.074)	.01	7155
pSLF (right)	-0.040 (-0.070 to -0.009)	.01	0.000 (-0.035 to 0.034)	.98	0.060 (0.028 to 0.092)	< .001	7155
pSLF (left)	-0.059 (-0.089 to -0.029)	< .001	0.011 (-0.023 to 0.044)	.53	0.065 (0.033 to 0.096)	< .001	7151
SCS (left)	0.005 (-0.024 to 0.035)	.72	0.004 (-0.030 to 0.037)	.82	0.042 (0.010 to 0.073)	.01	7155

© 2023 Li ZA et al. *JAMA Network Open*.

Abbreviations: ATR, anterior thalamic radiations; CC, corpus callosum; CgC, cingulate cingulum; CgH, parahippocampal cingulum; CI, confidence interval; CST, corticospinal/pyramidal tract; DTI, diffusion tensor imaging; DV, dependent variable; FA, fractional anisotropy; FDR, false discovery rate; Fmaj, forceps major; Fmin, forceps minor; Fx, fornix; IFOF, inferior frontal-occipital fasciculus; IFSFC, inferior-frontal to superior-frontal cortical tract; ILF, inferior longitudinal fasciculus; IV, independent variable; MD, mean diffusivity; pSLF, parietal superior longitudinal fasciculus; RND, restricted normalized directional; RNI, restricted normalized isotropic; RSI, restriction spectrum imaging; SCS, superior-corticostriatal tract; SES, socioeconomic status; SIFC, striatal to inferior-frontal cortical tract; SLF, superior longitudinal fasciculus; tSLF, temporal superior longitudinal fasciculus; Unc, uncinate fasciculus

[a] Sensitivity analyses were conducted on significant associations between SES and white matter microstructure that were observed in our study sample. Associations that were significant in the main analyses (**eTable 9** in the **Supplement**) and are nominally significant (*p*-value ≤ .05) here in sensitivity analyses were considered robust and are demarcated in green. Associations that were significant in the main analyses, but not nominally significant here, are demarcated in gray. Unhighlighted associations were not significant in the main analyses. Most findings were robust to sensitivity analyses. Findings that failed to replicate mostly involve associations between household income and RSI-RNI in distributed white matter tracts. Research has reported on white matter alterations in children and adolescents who experienced childhood adversity[19,20], had early-onset psychosis[21], had major depression[22], and who were born pre-term[23]. In particular, neuroinflammatory phenotypes, which RSI-RNI could potentially reflect, were seen in white matter regions in those who had adverse childhood experiences[19] or depression[22]. It is possible that these associations were reflected in our main analyses but were excluded in these more restricted subsamples. Additionally, the subsample of participants without reported adverse childhood experience had about 36% reduction in sample size relative to the whole study sample; it is thus possible that some associations became nonsignificant due to decreased statistical power. Model setup was identical to main analyses.

© 2023 Li ZA et al. *JAMA Network Open*.

eFigure 6. Associations Between SES and White Matter DTI FA

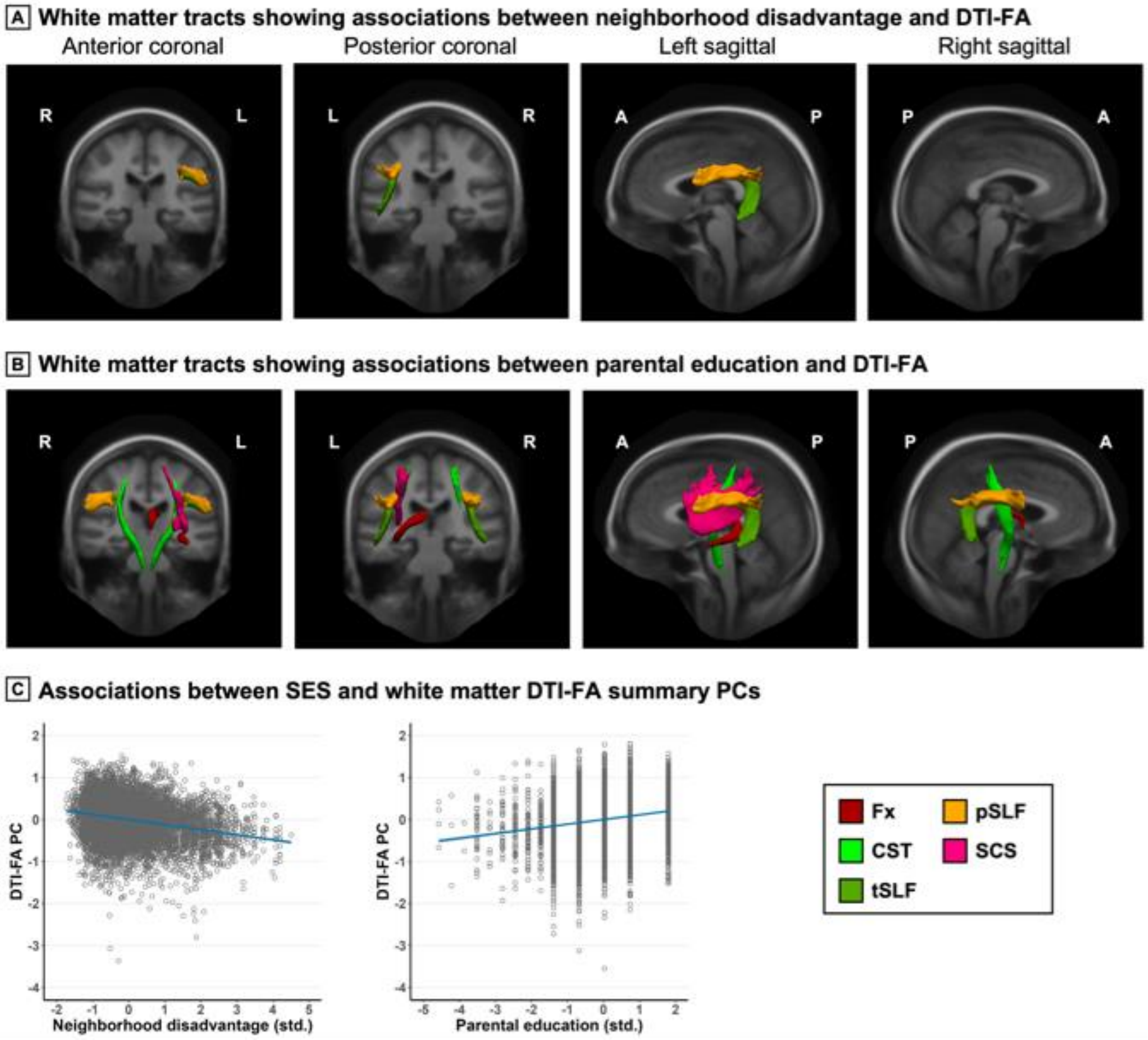

Associations between SES and white matter DTI-FA. Lower SES facets, including greater neighborhood disadvantage and lower parental education, were independently associated with lower diffusion tensor imaging – fractional anisotropy (DTI-FA) diffusion in white matter tracts. Tracts were visualized using the AtlasTrack atlas. The principal components (PCs) summarized DTI-FA in the involved tracts, as associations did not differ qualitatively between tracts. In scatterplots, linear regression lines were adjusted for covariates and flanked by shaded 95% confidence intervals. Data points were standardized (std.) residuals extracted from a randomly selected multiply imputed dataset (out of 50) as reference. Covariates included age, sex, pubertal development stage, intracranial volume, and head motion, and family was the random effect. Detailed statistics are shown in **eTable 9** and **eTable 13** in the **Supplement**. A, anterior; CST, corticospinal/pyramidal tract; Fmaj, forceps major; Fx, fornix; L, left hemisphere; P, posterior; R, right hemisphere; SES, socioeconomic status; SCS, superior-corticostriatal tract; SLF, superior longitudinal fasciculus, including temporal (t) and parietal (p) subregions

© 2023 Li ZA et al. *JAMA Network Open*.

eTable 11. Associations Between SES and White Matter Microstructure Adjusted for Race and Ethnicity[a]

White matter tracts	SES indicators (IVs)								
	Neighborhood disadvantage (higher = lower SES)			Household income (higher = higher SES)			Parental education (higher = higher SES)		
	**β (95% CI)**	***p*-value** (nominal)	***p*-value** (FDR)	**β (95% CI)**	***p*-value** (nominal)	***p*-value** (FDR)	**β (95% CI)**	***p*-value** (nominal)	***p*-value** (FDR)
**A) RSI-RND (DV)**									
Fx (right)	0.011 (-0.018 to 0.039)	.47	.85	0.020 (-0.012 to 0.051)	.22	.91	0.028 (-0.001 to 0.057)	.06	.12
Fx (left)	0.008 (-0.021 to 0.037)	.59	.85	0.005 (-0.027 to 0.037)	.75	.91	0.027 (-0.002 to 0.056)	.07	.13
CgC (right)	0.002 (-0.026 to 0.030)	.88	.93	-0.016 (-0.046 to 0.015)	.31	.91	0.015 (-0.013 to 0.043)	.31	.41
CgC (left)	-0.005 (-0.034 to 0.023)	.71	.85	-0.010 (-0.040 to 0.021)	.53	.91	-0.008 (-0.036 to 0.020)	.57	.63
CgH (right)	-0.014 (-0.042 to 0.015)	.34	.85	-0.008 (-0.039 to 0.022)	.59	.91	0.033 (0.004 to 0.061)	.02	.08
CgH (left)	0.008 (-0.020 to 0.036)	.58	.85	0.010 (-0.021 to 0.040)	.54	.91	0.030 (0.002 to 0.058)	.04	.10
CST (right)	0.006 (-0.021 to 0.034)	.66	.85	0.017 (-0.013 to 0.047)	.26	.91	0.040 (0.012 to 0.068)	.005	**.02**
CST (left)	0.016 (-0.012 to 0.044)	.26	.85	0.013 (-0.017 to 0.044)	.39	.91	0.049 (0.020 to 0.077)	.001	**.004**
ATR (right)	0.021 (-0.008 to 0.049)	.15	.85	0.024 (-0.007 to 0.056)	.13	.91	0.015 (-0.014 to 0.044)	.31	.41
ATR (left)	0.008 (-0.021 to 0.037)	.60	.85	0.027 (-0.005 to 0.058)	.10	.91	0.016 (-0.013 to 0.045)	.27	.40
Unc (right)	-0.005 (-0.034 to 0.023)	.71	.85	-0.029 (-0.059 to 0.001)	.06	.91	0.012 (-0.015 to 0.040)	.38	.48
Unc (left)	0.014 (-0.015 to 0.043)	.33	.85	-0.023 (-0.053 to 0.007)	.13	.91	0.014 (-0.015 to 0.042)	.34	.44
ILF (right)	0.003 (-0.025 to 0.031)	.82	.90	-0.017 (-0.048 to 0.013)	.26	.91	0.026 (-0.002 to 0.054)	.07	.13
ILF (left)	0.025 (-0.003 to 0.053)	.09	.66	-0.005 (-0.036 to 0.025)	.74	.91	0.021 (-0.007 to 0.049)	.14	.22
IFOF (right)	-0.020 (-0.048 to 0.008)	.17	.85	-0.013 (-0.044 to 0.017)	.38	.91	0.031 (0.003 to 0.059)	.03	.10
IFOF (left)	-0.013 (-0.041 to 0.015)	.38	.85	-0.009 (-0.039 to 0.020)	.54	.91	0.027 (0.000 to 0.055)	.05	.12
Fmaj	-0.011 (-0.040 to 0.017)	.44	.85	0.005 (-0.026 to 0.035)	.77	.91	-0.010 (-0.038 to 0.019)	.51	.58
Fmin	0.011 (-0.018 to 0.039)	.47	.85	-0.013 (-0.043 to 0.018)	.42	.91	-0.010 (-0.038 to 0.018)	.49	.58
CC	-0.006 (-0.034 to 0.022)	.68	.85	-0.005 (-0.035 to 0.025)	.74	.91	-0.003 (-0.031 to 0.025)	.83	.86
SLF (right)	-0.013 (-0.041 to 0.016)	.38	.85	-0.003 (-0.033 to 0.028)	.86	.94	0.051 (0.024 to 0.079)	< .001	**.003**
SLF (left)	-0.032 (-0.060 to -0.004)	.02	.53	0.001 (-0.029 to 0.031)	.94	.94	0.049 (0.021 to 0.076)	.001	**.004**
tSLF (right)	-0.011 (-0.040 to 0.017)	.45	.85	-0.005 (-0.035 to 0.026)	.78	.91	0.043 (0.014 to 0.071)	.003	**.02**
tSLF (left)	-0.030 (-0.058 to -0.002)	.03	.53	0.004 (-0.026 to 0.034)	.79	.91	0.040 (0.013 to 0.068)	.004	**.02**
pSLF (right)	-0.014 (-0.043 to 0.014)	.31	.85	-0.002 (-0.032 to 0.028)	.89	.94	0.052 (0.024 to 0.080)	< .001	**.003**
pSLF (left)	-0.028 (-0.055 to 0.000)	.05	.53	-0.004 (-0.034 to 0.026)	.79	.91	0.058 (0.031 to 0.086)	< .001	**.001**
SCS (right)	0.010 (-0.019 to 0.038)	.50	.85	-0.011 (-0.042 to 0.020)	.49	.91	0.030 (0.001 to 0.058)	.04	.10
SCS (left)	0.010 (-0.018 to 0.038)	.49	.85	-0.001 (-0.031 to 0.029)	.94	.94	0.029 (0.001 to 0.056)	.04	.11
SIFC (right)	0.000 (-0.030 to 0.029)	.98	.98	-0.016 (-0.046 to 0.015)	.32	.91	-0.004 (-0.032 to 0.025)	.81	.86
SIFC (left)	-0.007 (-0.036 to 0.021)	.61	.85	-0.015 (-0.045 to 0.015)	.33	.91	0.000 (-0.028 to 0.028)	> .99	> .99
IFSFC (right)	-0.004 (-0.032 to 0.024)	.76	.88	0.013 (-0.017 to 0.043)	.39	.91	0.023 (-0.004 to 0.051)	.10	.17
IFSFC (left)	-0.002 (-0.029 to 0.026)	.90	.93	0.006 (-0.022 to 0.036)	.67	.91	0.023 (-0.005 to 0.050)	.10	.17

© 2023 Li ZA et al. *JAMA Network Open*.

White matter tracts	SES indicators (IVs)								
	Neighborhood disadvantage (higher = lower SES)			Household income (higher = higher SES)			Parental education (higher = higher SES)		
	β **(95% CI)**	***p*-value** (nominal)	***p*-value** (FDR)	***β*** **(95% CI)**	***p*-value** (nominal)	***p*-value** (FDR)	***β*** **(95% CI)**	***p*-value** (nominal)	***p*-value** (FDR)
**B) RSI-RNI (DV)**									
Fx (right)	0.029 (0.001 to 0.057)	.05	.70	0.018 (-0.013 to 0.050)	.26	.31	-0.004 (-0.033 to 0.025)	.78	.96
Fx (left)	0.024 (-0.004 to 0.053)	.09	.70	0.009 (-0.023 to 0.041)	.58	.62	-0.010 (-0.039 to 0.019)	.48	.96
CgC (right)	0.008 (-0.020 to 0.036)	.56	.84	-0.026 (-0.056 to 0.005)	.10	.14	-0.002 (-0.030 to 0.026)	.90	.96
CgC (left)	0.007 (-0.021 to 0.036)	.62	.84	-0.032 (-0.063 to -0.001)	.05	.09	-0.007 (-0.036 to 0.022)	.63	.96
CgH (right)	0.018 (-0.010 to 0.046)	.20	.70	-0.007 (-0.037 to 0.023)	.65	.66	0.013 (-0.016 to 0.041)	.38	.96
CgH (left)	0.021 (-0.008 to 0.049)	.15	.70	-0.025 (-0.055 to 0.005)	.11	.14	0.005 (-0.023 to 0.033)	.71	.96
CST (right)	-0.003 (-0.031 to 0.026)	.86	.99	-0.007 (-0.038 to 0.024)	.66	.66	0.005 (-0.023 to 0.034)	.73	.96
CST (left)	-0.007 (-0.035 to 0.021)	.62	.84	-0.015 (-0.046 to 0.016)	.34	.39	0.001 (-0.027 to 0.029)	.94	.96
ATR (right)	0.018 (-0.010 to 0.046)	.21	.70	-0.031 (-0.062 to -0.001)	.05	.09	0.012 (-0.017 to 0.040)	.42	.96
ATR (left)	0.011 (-0.018 to 0.039)	.45	.83	-0.026 (-0.057 to 0.006)	.11	.14	-0.001 (-0.029 to 0.028)	.96	.96
Unc (right)	-0.006 (-0.035 to 0.022)	.66	.85	-0.046 (-0.077 to -0.015)	.003	**.05**	0.015 (-0.014 to 0.043)	.31	.96
Unc (left)	-0.004 (-0.032 to 0.025)	.79	.94	-0.042 (-0.073 to -0.011)	.01	**.05**	0.006 (-0.023 to 0.034)	.70	.96
ILF (right)	-0.019 (-0.047 to 0.009)	.19	.70	-0.034 (-0.065 to -0.003)	.03	.08	0.003 (-0.025 to 0.031)	.82	.96
ILF (left)	-0.007 (-0.036 to 0.021)	.61	.84	-0.029 (-0.060 to 0.002)	.06	.12	-0.008 (-0.036 to 0.021)	.59	.96
IFOF (right)	-0.017 (-0.045 to 0.011)	.23	.70	-0.047 (-0.077 to -0.016)	.003	**.05**	0.002 (-0.026 to 0.030)	.89	.96
IFOF (left)	-0.015 (-0.042 to 0.013)	.30	.70	-0.043 (-0.074 to -0.013)	.01	**.05**	-0.002 (-0.031 to 0.026)	.87	.96
Fmaj	0.008 (-0.021 to 0.037)	.57	.84	-0.011 (-0.042 to 0.020)	.50	.55	-0.040 (-0.068 to -0.011)	.01	.20
Fmin	0.001 (-0.028 to 0.030)	.94	.99	-0.040 (-0.071 to -0.009)	.01	.06	-0.029 (-0.058 to 0.000)	.05	.80
CC	-0.005 (-0.033 to 0.024)	.75	.93	-0.034 (-0.065 to -0.002)	.04	.09	-0.017 (-0.046 to 0.012)	.25	.96
SLF (right)	-0.013 (-0.041 to 0.015)	.37	.71	-0.027 (-0.057 to 0.003)	.08	.12	-0.004 (-0.032 to 0.024)	.76	.96
SLF (left)	0.000 (-0.028 to 0.029)	.99	.99	-0.037 (-0.068 to -0.007)	.02	.06	0.002 (-0.026 to 0.030)	.88	.96
tSLF (right)	-0.010 (-0.038 to 0.018)	.50	.84	-0.025 (-0.055 to 0.006)	.11	.14	-0.002 (-0.030 to 0.026)	.89	.96
tSLF (left)	0.001 (-0.028 to 0.029)	.95	.99	-0.037 (-0.068 to -0.007)	.02	.06	0.004 (-0.024 to 0.032)	.79	.96
pSLF (right)	-0.014 (-0.042 to 0.014)	.34	.70	-0.027 (-0.057 to 0.003)	.08	.12	-0.003 (-0.031 to 0.025)	.83	.96
pSLF (left)	-0.001 (-0.029 to 0.028)	.97	.99	-0.037 (-0.068 to -0.007)	.02	.06	0.006 (-0.022 to 0.034)	.69	.96
SCS (right)	-0.015 (-0.043 to 0.013)	.29	.70	-0.022 (-0.053 to 0.008)	.15	.19	0.014 (-0.014 to 0.042)	.34	.96
SCS (left)	-0.015 (-0.042 to 0.013)	.29	.70	-0.029 (-0.060 to 0.001)	.06	.11	0.010 (-0.018 to 0.038)	.49	.96
SIFC (right)	-0.014 (-0.043 to 0.015)	.34	.70	-0.043 (-0.074 to -0.012)	.01	**.05**	0.001 (-0.028 to 0.030)	.94	.96
SIFC (left)	-0.019 (-0.047 to 0.009)	.19	.70	-0.029 (-0.060 to 0.002)	.07	.12	0.001 (-0.027 to 0.030)	.94	.96
IFSFC (right)	-0.017 (-0.045 to 0.011)	.23	.70	-0.035 (-0.066 to -0.004)	.03	.08	0.014 (-0.015 to 0.042)	.34	.96
IFSFC (left)	-0.023 (-0.051 to 0.006)	.11	.70	-0.035 (-0.066 to -0.004)	.03	.08	0.015 (-0.013 to 0.043)	.30	.96

© 2023 Li ZA et al. *JAMA Network Open*.

White matter tracts	SES indicators (IVs)								
	**Neighborhood disadvantage** (higher = lower SES)			**Household income** (higher = higher SES)			**Parental education** (higher = higher SES)		
	***β* (95% CI)**	***p*-value** (nominal)	***p*-value** (FDR)	***β* (95% CI)**	***p*-value** (nominal)	***p*-value** (FDR)	***β* (95% CI)**	***p*-value** (nominal)	***p*-value** (FDR)
**C) DTI-FA (DV)**									
Fx (right)	0.004 (-0.025 to 0.033)	.79	.94	0.022 (-0.009 to 0.053)	.17	.98	0.029 (0.000 to 0.058)	.05	.10
Fx (left)	0.001 (-0.027 to 0.030)	.93	.96	-0.005 (-0.036 to 0.026)	.75	.98	0.038 (0.009 to 0.066)	.01	**.03**
CgC (right)	0.000 (-0.029 to 0.028)	.98	.98	-0.024 (-0.055 to 0.007)	.13	.98	0.010 (-0.018 to 0.039)	.49	.61
CgC (left)	-0.006 (-0.035 to 0.023)	.68	.94	-0.015 (-0.045 to 0.016)	.35	.98	-0.008 (-0.036 to 0.021)	.60	.69
CgH (right)	-0.010 (-0.039 to 0.018)	.47	.94	-0.003 (-0.033 to 0.028)	.86	.98	0.011 (-0.017 to 0.040)	.43	.58
CgH (left)	0.004 (-0.025 to 0.032)	.80	.94	0.014 (-0.017 to 0.044)	.38	.98	0.031 (0.002 to 0.059)	.03	.09
CST (right)	0.006 (-0.022 to 0.034)	.69	.94	0.002 (-0.029 to 0.032)	.92	.98	0.044 (0.016 to 0.072)	.002	**.01**
CST (left)	0.026 (-0.002 to 0.055)	.07	.46	0.002 (-0.029 to 0.033)	.90	.98	0.046 (0.018 to 0.074)	.002	**.01**
ATR (right)	0.017 (-0.012 to 0.046)	.25	.78	0.018 (-0.013 to 0.050)	.25	.98	0.016 (-0.013 to 0.044)	.29	.41
ATR (left)	0.009 (-0.021 to 0.038)	.57	.94	0.030 (-0.002 to 0.061)	.06	.98	0.018 (-0.011 to 0.047)	.23	.33
Unc (right)	-0.008 (-0.037 to 0.022)	.60	.94	-0.020 (-0.051 to 0.011)	.20	.98	0.005 (-0.024 to 0.033)	.74	.79
Unc (left)	0.006 (-0.024 to 0.035)	.71	.94	-0.016 (-0.047 to 0.015)	.32	.98	0.008 (-0.021 to 0.036)	.60	.69
ILF (right)	0.004 (-0.025 to 0.032)	.80	.94	-0.008 (-0.039 to 0.023)	.61	.98	0.025 (-0.003 to 0.053)	.08	.15
ILF (left)	0.020 (-0.009 to 0.049)	.17	.67	0.004 (-0.027 to 0.035)	.79	.98	0.021 (-0.008 to 0.049)	.15	.25
IFOF (right)	-0.017 (-0.046 to 0.011)	.23	.78	-0.011 (-0.042 to 0.019)	.47	.98	0.035 (0.007 to 0.064)	.01	**.04**
IFOF (left)	-0.005 (-0.033 to 0.024)	.74	.94	0.004 (-0.027 to 0.034)	.81	.98	0.030 (0.002 to 0.058)	.04	.09
Fmaj	-0.003 (-0.032 to 0.026)	.82	.94	0.000 (-0.031 to 0.031)	.99	.99	0.010 (-0.018 to 0.039)	.47	.61
Fmin	0.007 (-0.023 to 0.037)	.63	.94	-0.011 (-0.043 to 0.020)	.48	.98	-0.021 (-0.050 to 0.009)	.17	.26
CC	-0.003 (-0.031 to 0.026)	.85	.95	-0.009 (-0.040 to 0.022)	.57	.98	0.002 (-0.027 to 0.030)	.91	.94
SLF (right)	-0.025 (-0.055 to 0.004)	.09	.46	-0.008 (-0.040 to 0.024)	.62	.98	0.049 (0.020 to 0.078)	.001	**.01**
SLF (left)	-0.036 (-0.065 to -0.007)	.01	.24	-0.002 (-0.033 to 0.029)	.89	.98	0.048 (0.019 to 0.076)	.001	**.01**
tSLF (right)	-0.023 (-0.052 to 0.006)	.12	.54	-0.014 (-0.045 to 0.018)	.39	.98	0.042 (0.013 to 0.071)	.005	**.02**
tSLF (left)	-0.033 (-0.062 to -0.005)	.02	.24	0.001 (-0.030 to 0.032)	.95	.98	0.040 (0.011 to 0.068)	.01	**.03**
pSLF (right)	-0.025 (-0.054 to 0.004)	.09	.46	-0.005 (-0.036 to 0.027)	.78	.98	0.048 (0.020 to 0.077)	.001	**.01**
pSLF (left)	-0.034 (-0.063 to -0.005)	.02	.24	-0.006 (-0.036 to 0.025)	.72	.98	0.058 (0.029 to 0.086)	< .001	**.002**
SCS (right)	0.011 (-0.017 to 0.040)	.44	.94	-0.009 (-0.040 to 0.022)	.58	.98	0.030 (0.001 to 0.059)	.04	.09
SCS (left)	0.014 (-0.014 to 0.042)	.33	.93	-0.001 (-0.032 to 0.029)	.93	.98	0.035 (0.007 to 0.064)	.01	**.04**
SIFC (right)	-0.002 (-0.031 to 0.028)	.92	.96	-0.022 (-0.054 to 0.009)	.16	.98	0.006 (-0.023 to 0.035)	.70	.78
SIFC (left)	-0.003 (-0.033 to 0.026)	.82	.94	-0.010 (-0.041 to 0.021)	.53	.98	0.000 (-0.028 to 0.029)	.98	.98
IFSFC (right)	-0.009 (-0.038 to 0.020)	.54	.94	0.003 (-0.028 to 0.034)	.84	.98	0.026 (-0.002 to 0.055)	.07	.14
IFSFC (left)	-0.005 (-0.033 to 0.024)	.75	.94	-0.002 (-0.033 to 0.029)	.88	.98	0.023 (-0.005 to 0.052)	.11	.19

© 2023 Li ZA et al. *JAMA Network Open*.

White matter tracts	SES indicators (IVs)								
	**Neighborhood disadvantage** (higher = lower SES)			**Household income** (higher = higher SES)			**Parental education** (higher = higher SES)		
	β **(95% CI)**	***p*-value** (nominal)	***p*-value** (FDR)	***β*** **(95% CI)**	***p*-value** (nominal)	***p*-value** (FDR)	***β*** **(95% CI)**	***p*-value** (nominal)	***p*-value** (FDR)
**D) DTI-MD (DV)**									
Fx (right)	-0.002 (-0.030 to 0.027)	.92	.93	-0.024 (-0.055 to 0.007)	.12	.26	0.008 (-0.021 to 0.037)	.59	.76
Fx (left)	-0.005 (-0.034 to 0.023)	.71	.93	-0.009 (-0.041 to 0.022)	.55	.64	0.008 (-0.021 to 0.037)	.58	.76
CgC (right)	-0.005 (-0.034 to 0.024)	.74	.93	0.005 (-0.026 to 0.037)	.74	.77	0.004 (-0.025 to 0.033)	.79	.87
CgC (left)	-0.005 (-0.035 to 0.024)	.71	.93	0.017 (-0.014 to 0.048)	.27	.43	-0.003 (-0.032 to 0.025)	.82	.87
CgH (right)	0.002 (-0.027 to 0.032)	.87	.93	0.011 (-0.020 to 0.042)	.50	.62	-0.015 (-0.043 to 0.014)	.32	.59
CgH (left)	0.004 (-0.025 to 0.033)	.78	.93	0.026 (-0.005 to 0.057)	.10	.24	-0.011 (-0.040 to 0.017)	.43	.61
CST (right)	0.011 (-0.018 to 0.040)	.45	.81	0.017 (-0.014 to 0.048)	.30	.44	-0.011 (-0.040 to 0.017)	.43	.61
CST (left)	0.005 (-0.024 to 0.035)	.72	.93	0.008 (-0.023 to 0.040)	.60	.65	-0.002 (-0.031 to 0.026)	.87	.87
ATR (right)	0.001 (-0.028 to 0.031)	.93	.93	0.033 (0.002 to 0.065)	.04	.22	-0.017 (-0.046 to 0.011)	.23	.59
ATR (left)	0.005 (-0.024 to 0.034)	.73	.93	0.021 (-0.010 to 0.052)	.19	.34	-0.003 (-0.031 to 0.026)	.84	.87
Unc (right)	0.029 (0.000 to 0.058)	.05	.54	0.029 (-0.003 to 0.061)	.07	.22	-0.013 (-0.042 to 0.016)	.37	.59
Unc (left)	0.013 (-0.017 to 0.042)	.40	.77	0.021 (-0.010 to 0.053)	.19	.34	-0.004 (-0.033 to 0.025)	.81	.87
ILF (right)	0.026 (-0.002 to 0.055)	.07	.54	0.015 (-0.016 to 0.046)	.35	.47	-0.019 (-0.047 to 0.009)	.19	.59
ILF (left)	0.018 (-0.010 to 0.047)	.21	.58	0.008 (-0.023 to 0.039)	.60	.65	-0.016 (-0.044 to 0.012)	.26	.59
IFOF (right)	0.029 (0.000 to 0.057)	.05	.54	0.034 (0.004 to 0.065)	.03	.22	-0.016 (-0.044 to 0.012)	.27	.59
IFOF (left)	0.019 (-0.010 to 0.047)	.21	.58	0.031 (0.000 to 0.061)	.05	.22	-0.013 (-0.041 to 0.016)	.38	.59
Fmaj	-0.002 (-0.031 to 0.027)	.90	.93	-0.002 (-0.033 to 0.029)	.90	.90	0.031 (0.002 to 0.060)	.04	.57
Fmin	-0.003 (-0.033 to 0.027)	.86	.93	0.010 (-0.022 to 0.042)	.54	.64	0.031 (0.002 to 0.060)	.04	.57
CC	0.008 (-0.021 to 0.037)	.59	.93	0.014 (-0.017 to 0.045)	.38	.49	0.020 (-0.009 to 0.049)	.17	.59
SLF (right)	0.024 (-0.005 to 0.053)	.10	.54	0.030 (-0.001 to 0.061)	.06	.22	-0.024 (-0.053 to 0.004)	.09	.59
SLF (left)	0.017 (-0.012 to 0.046)	.26	.63	0.028 (-0.003 to 0.059)	.08	.22	-0.015 (-0.043 to 0.014)	.32	.59
tSLF (right)	0.020 (-0.009 to 0.048)	.18	.58	0.035 (0.004 to 0.065)	.03	.22	-0.027 (-0.055 to 0.002)	.06	.59
tSLF (left)	0.016 (-0.013 to 0.045)	.28	.63	0.028 (-0.003 to 0.059)	.08	.22	-0.013 (-0.042 to 0.015)	.36	.59
pSLF (right)	0.026 (-0.003 to 0.054)	.08	.54	0.029 (-0.002 to 0.060)	.07	.22	-0.023 (-0.051 to 0.005)	.11	.59
pSLF (left)	0.019 (-0.010 to 0.048)	.20	.58	0.030 (-0.001 to 0.061)	.06	.22	-0.016 (-0.045 to 0.012)	.27	.59
SCS (right)	0.024 (-0.005 to 0.052)	.10	.54	0.027 (-0.004 to 0.058)	.09	.23	-0.019 (-0.047 to 0.009)	.19	.59
SCS (left)	0.009 (-0.020 to 0.038)	.53	.92	0.018 (-0.013 to 0.049)	.25	.40	-0.013 (-0.042 to 0.015)	.37	.59
SIFC (right)	0.022 (-0.008 to 0.051)	.15	.58	0.032 (0.001 to 0.063)	.04	.22	-0.017 (-0.046 to 0.012)	.24	.59
SIFC (left)	0.005 (-0.025 to 0.034)	.76	.93	0.015 (-0.016 to 0.046)	.35	.47	0.006 (-0.023 to 0.035)	.67	.80
IFSFC (right)	0.016 (-0.013 to 0.045)	.28	.63	0.020 (-0.012 to 0.051)	.22	.38	-0.018 (-0.046 to 0.011)	.22	.59
IFSFC (left)	0.014 (-0.016 to 0.043)	.36	.74	0.024 (-0.007 to 0.056)	.13	.26	-0.007 (-0.036 to 0.022)	.64	.80

© 2023 Li ZA et al. *JAMA Network Open*.

Abbreviations: ATR, anterior thalamic radiations; CC, corpus callosum; CgC, cingulate cingulum; CgH, parahippocampal cingulum; CI, confidence interval; CST, corticospinal/pyramidal tract; DTI, diffusion tensor imaging; DV, dependent variable; FA, fractional anisotropy; FDR, false discovery rate; Fmaj, forceps major; Fmin, forceps minor; Fx, fornix; IFOF, inferior frontal-occipital fasciculus; IFSFC, inferior-frontal to superior-frontal cortical tract; ILF, inferior longitudinal fasciculus; IV, independent variable; MD, mean diffusivity; pSLF, parietal superior longitudinal fasciculus; RND, restricted normalized directional; RNI, restricted normalized isotropic; RSI, restriction spectrum imaging; SCS, superior-corticostriatal tract; SES, socioeconomic status; SIFC, striatal to inferior-frontal cortical tract; SLF, superior longitudinal fasciculus; tSLF, temporal superior longitudinal fasciculus; Unc, uncinate fasciculus

[a] Linear mixed effects models included all three SES indicators simultaneously as IVs and RSI and DTI metrics in each of the white matter tracts as the dependent variable DV. Models were adjusted for participant age, sex, pubertal development stage, intracranial volume, mean head motion, and race/ethnicity, and were nested by family. More restricted results were observed here compared to models that did not covary for race/ethnicity (**eTable 9** in the **Supplement**), possibly as a reflection of the high confounding between SES and race/ethnicity (see **eTable 7** in the **Supplement**).Multiple comparison correction was conducted within each neuroimaging metric and by each SES indicator, giving 31 tests to correct for in each group of models. Associations that survived FDR-corrected *p*-value ≤ .05 were considered statistically significant and are highlighted. Estimates were standardized *β*'s with 95% CIs.

eTable 12. PCA Loadings of RSI and DTI Measurements That Were Significantly Associated With SES[a]

1) Results for tracts significantly associated with neighborhood disadvantage

**Metric**	**Principal component (PC)**	
	**PC1**	**PC2**
**A) RSI-RND**		
Loadings		
Fmaj	0.32	0.95
SLF (left)	0.56	-0.19
tSLF (left)	0.55	-0.17
pSLF (left)	0.54	-0.19
Proportion of variance	0.78	0.20
Cumulative proportion	0.78	0.97
**B) RSI-RNI**		
Loadings		
Fx (right)	-0.34	-0.52
Fx (left)	-0.34	-0.53
CgH (right)	-0.33	0.48
CgH (left)	-0.34	0.46
CST (right)	-0.36	0.06
CST (left)	-0.36	0.08
ATR (right)	-0.38	-0.02
ATR (left)	-0.38	-0.02
Proportion of variance	0.59	0.13
Cumulative proportion	0.59	0.72
**C) DTI-FA**		
Loadings		
SLF (left)	-0.59	-0.17
tSLF (left)	-0.58	-0.60
pSLF (left)	-0.56	0.78
Proportion of variance	0.95	0.05
Cumulative proportion	0.95	1.00

2) Results for tracts significantly associated with household income

**Metric**	**Principal component (PC)**	
	**PC1**	**PC2**
**A) RSI-RNI**		
Loadings		
CgC (right)	-0.17	0.04
CgC (left)	-0.17	0.01
CgH (right)	-0.16	0.16
CgH (left)	-0.16	0.14
CST (right)	-0.16	-0.33
CST (left)	-0.16	-0.33
ATR (right)	-0.19	0.13
ATR (left)	-0.19	0.12
Unc (right)	-0.19	0.28
Unc (left)	-0.19	0.27
ILF (right)	-0.19	0.12
ILF (left)	-0.20	0.11
IFOF (right)	-0.21	0.17
IFOF (left)	-0.21	0.15
Fmin	-0.19	0.23
CC	-0.21	0.03
SLF (right)	-0.20	-0.21
SLF (left)	-0.21	-0.21
tSLF (left)	-0.20	-0.20
pSLF (right)	-0.21	-0.18
pSLF (left)	-0.20	-0.26
SCS (left)	-0.21	-0.23

© 2023 Li ZA et al. *JAMA Network Open*.

SIFC (right)	-0.20	0.22
SIFC (left)	-0.20	0.19
IFSFC (right)	-0.21	-0.09
IFSFC (left)	-0.21	-0.07
Proportion of variance	0.66	0.06
Cumulative proportion	0.66	0.72

3) Results for tracts significantly associated with parental education

**Metric**	**Principal component (PC)**	
	**PC1**	**PC2**
**A) RSI-RND**		
Loadings		
CST (right)	-0.22	-0.67
CST (left)	-0.22	-0.67
SLF (right)	-0.40	0.13
SLF (left)	-0.40	0.13
tSLF (right)	-0.38	0.14
tSLF (left)	-0.39	0.14
pSLF (right)	-0.39	0.13
pSLF (left)	-0.38	0.11
Proportion of variance	0.70	0.17
Cumulative proportion	0.70	0.87
**B) RSI-RNI**		
Loadings		
Fmaj	NA	NA
Proportion of variance	NA	NA
Cumulative proportion	NA	NA
**C) DTI-FA**		
Loadings		
Fx (left)	-0.12	0.21
CST (right)	-0.23	0.50
CST (left)	-0.24	0.56
SLF (right)	-0.38	-0.19
SLF (left)	-0.38	-0.17
tSLF (right)	-0.36	-0.18
tSLF (left)	-0.37	-0.17
pSLF (right)	-0.37	-0.18
pSLF (left)	-0.36	-0.16
SCS (left)	-0.25	0.46
Proportion of variance	0.58	0.17
Cumulative proportion	0.58	0.75

Abbreviations: ATR, anterior thalamic radiations; CC, corpus callosum; CgC, cingulate cingulum; CgH, parahippocampal cingulum; CST, corticospinal/pyramidal tract; DTI, diffusion tensor imaging; FA, fractional anisotropy; Fmaj, forceps major; Fmin, forceps minor; Fx, fornix; IFOF, inferior frontal-occipital fasciculus; IFSFC, inferior-frontal to superior-frontal cortical tract; ILF, inferior longitudinal fasciculus; IV, independent variable; PC, principal component; pSLF, parietal superior longitudinal fasciculus; RND, restricted normalized directional; RNI, restricted normalized isotropic; RSI, restriction spectrum imaging; SCS, superior-corticostriatal tract; SES, socioeconomic status; SIFC, striatal to inferior-frontal cortical tract; SLF, superior longitudinal fasciculus; tSLF, temporal superior longitudinal fasciculus; Unc, uncinate fasciculus

[a] Principal component analyses (PCA) was conducted with the "psych" package using singular value decomposition[24]. In a randomly selected imputed dataset out of 50, the first principal component (i.e., PC1) had strong loadings from all relevant tracts and captured substantial variance (58% to 95%) in RSI or DTI metrics; these PCs would serve as white matter microstructure composite scores in indirect associations models. Some PCs would be reverse-coded to ensure consistent direction of associations with SES indicators (i.e., higher SES, higher RSI-RND, lower RSI-RNI, and higher DTI-FA). Because parental education was associated with RSI-RNI only in Fmaj, no PCA was applied there.

© 2023 Li ZA et al. *JAMA Network Open*.

eTable 13. Associations Between SES and White Matter Microstructure PCs[a]

White matter microstructure principal components (PCs)	SES indicators (IVs)						Sample size (*n*)
	**Neighborhood disadvantage (ND)** (higher = lower SES)		**Household income (HI)** (higher = higher SES)		**Parental education (PE)** (higher = higher SES)		
	***β* (95% CI)**	***p*-value** (nominal)	***β* (95% CI)**	***p*-value** (nominal)	***β* (95% CI)**	***p*-value** (nominal)	
**A) RSI-RND (DV)**							
PC1 – ND	**-0.055 (-0.081 to -0.028)**	**< .001**	0.016 (-0.013 to 0.046)	.28	0.044 (0.017 to 0.071)	.002	8799
PC2 – ND	0.011 (-0.017 to 0.039)	.43	-0.018 (-0.049 to 0.014)	.27	0.045 (0.016 to 0.074)	.002	8799
PC1 – PE	0.030 (0.003 to 0.056)	.03	-0.009 (-0.039 to 0.021)	.56	**-0.056 (-0.084 to -0.029)***	**< .001**	8787
PC2 – PE	-0.048 (-0.075 to -0.021)	.001	-0.010 (-0.041 to 0.020)	.50	-0.019 (-0.047 to 0.010)	.20	8787
**B) RSI-RNI (DV)**							
PC1 – ND	**-0.057 (-0.084 to -0.030)***	**< .001**	0.038 (0.008 to 0.069)	.01	0.006 (-0.022 to 0.035)	.65	8707
PC2 – ND	0.019 (-0.009 to 0.046)	.19	-0.043 (-0.075 to -0.012)	.01	0.017 (-0.012 to 0.045)	.26	8707
PC1 – HI	-0.006 (-0.033 to 0.021)	.67	**0.054 (0.023 to 0.084)***	**.001**	0.006 (-0.022 to 0.035)	.65	8658
PC2 – HI	0.008 (-0.020 to 0.036)	.57	-0.017 (-0.048 to 0.014)	.28	-0.015 (-0.044 to 0.014)	.30	8658
Fmaj – PE	0.002 (-0.025 to 0.030)	.88	-0.005 (-0.036 to 0.025)	.73	**-0.048 (-0.077 to -0.020)**	**.001**	8798
**C) DTI-FA (DV)**							
PC1 – ND	**0.064 (0.037 to 0.092)***	**< .001**	-0.015 (-0.046 to 0.016)	.34	-0.051 (-0.079 to -0.022)	< .001	8835
PC2 – ND	0.019 (-0.009 to 0.046)	.18	-0.024 (-0.055 to 0.007)	.12	0.033 (0.005 to 0.062)	.02	8835
PC1 – PE	0.042 (0.015 to 0.069)	.003	-0.007 (-0.038 to 0.024)	.65	**-0.059 (-0.087 to -0.030)***	**< .001**	8826
PC2 – PE	0.056 (0.029 to 0.083)	< .001	-0.003 (-0.033 to 0.027)	.85	0.018 (-0.010 to 0.046)	.20	8826

Abbreviations: CI, confidence interval; DTI, diffusion tensor imaging; DV, dependent variable; FA, fractional anisotropy; HI, household income; IV, independent variable; ND, neighborhood disadvantage; PC, principal component; PE, parental education; RND, restricted normalized directional; RNI, restricted normalized isotropic; RSI, restriction spectrum imaging; SES, socioeconomic status

[a] The first PCs (i.e., PC1) demonstrated strong associations to specific SES indicators (in bold), consistent with individual tracts from which the PCs were derived (see **eTable 9** in the **Supplement**). In comparison, the second PCs (i.e., PC2) did not demonstrate appreciable associations with SES. PC1s were therefore designated as white matter microstructure PCs used in structural equation models testing indirect associations. Associations here were examined with linear mixed-effects models with all three SES indicators as simultaneous independent variables (IVs) and each of the PCs as dependent variable (DV). Covariates included participant age, sex, PDS, ICV, and mean head motion as fixed effects, and family as random effect. * denotes PCs that would be reverse-coded to ensure consistent direction of associations with SES indicators (i.e., higher SES, higher RSI-RND, lower RSI-RNI, and higher DTI-FA); original unreversed PCs are shown here for accurate data representation. Estimates were standardized *β*'s with 95% confidence intervals (CIs). Results were pooled across all imputed datasets.

© 2023 Li ZA et al. *JAMA Network Open*.

eTable 14. Associations Between SES and Obesity-Related Measures[a]

Measures (DV)	SES indicators (IVs)					
	**Neighborhood disadvantage** (higher = lower SES)		**Household income** (higher = higher SES)		**Parental education** (higher = higher SES)	
	**β (95% CI)**	***p*-value** (FDR)	**β (95% CI)**	***p*-value** (FDR)	**β (95% CI)**	***p*-value** (FDR)
BMI	0.103 (0.076 to 0.129)	< .001	-0.067 (-0.097 to -0.037)	< .001	-0.106 (-0.133 to -0.078)	< .001
Waist circumference	0.082 (0.057 to 0.108)	< .001	-0.054 (-0.083 to -0.025)	< .001	-0.093 (-0.120 to -0.065)	< .001
BMI *z*-score	0.081 (0.055 to 0.107)	< .001	-0.062 (-0.091 to -0.033)	< .001	-0.089 (-0.116 to -0.061)	< .001

Abbreviations: BMI, body mass index; CI, confidence interval; DV, dependent variable; FDR, false discovery rate; IV, independent variable; SES, socioeconomic status

[a] Linear mixed-effects models included all three SES indicators simultaneously as IVs and RSI and DTI metrics in each of the obesity-related measures as the DV. Models were adjusted for participant age, sex, and PDS, and were nested by family. Multiple comparison was corrected for three tests. Associations that survived false discovery rate (FDR)-corrected p-value ≤ 0.05 were considered statistically significant. Estimates were standardized β's with 95% confidence intervals (CIs). SES, socioeconomic status; BMI, body mass index

© 2023 Li ZA et al. *JAMA Network Open*.

eTable 15. Indirect Associations Between SES and White Matter Microstructure via Obesity-Related Measures[a]

**Model**		**Path a (IV → mediator)**		**Path b (Mediator → DV, controlling for IV)**		**Path a × b (Indirect association)**				**Path c' (IV → DV, controlling for mediator)**		**Proportion mediated**
**IV**	**DV**	***β*** **(SE)**	***p*****-value** (nominal)	***β*** **(SE)**	***p*****-value** (nominal)	**Estimate (SE)**	***p*****-value** (nominal)	***p*****-value** (FDR)	**95% CI**	***β*** **(SE)**	***p*****-value** (nominal)	**%**
BMI as the mediator												
Neighborhood disadvantage	RSI-RND PC	0.108 (0.012)	< .001	-0.035 (0.011)	.001	-0.004 (0.001)	.002	**.003**	[-0.006, -0.001]	-0.049 (0.013)	< .001	7.55
	RSI-RNI PC	0.104 (0.012)	< .001	0.148 (0.011)	< .001	0.015 (0.002)	< .001	**< .001**	[0.011, 0.020]	0.042 (0.013)	.001	26.75
	DTI-FA PC	0.107 (0.012)	< .001	-0.001 (0.011)	.91	0.000 (0.001)	.91	.91	[-0.003, 0.002]	-0.065 (0.013)	< .001	0.21
Household income	RSI-RNI PC	-0.062 (0.014)	< .001	0.124 (0.011)	< .001	-0.008 (0.002)	< .001	**< .001**	[-0.011, -0.004]	-0.049 (0.015)	.001	13.75
Parental education	RSI-RND PC	-0.103 (0.013)	< .001	-0.047 (0.011)	< .001	0.005 (0.001)	< .001	**.007**	[0.003, 0.008]	0.053 (0.014)	< .001	8.44
	RSI-RNI Fmaj	-0.106 (0.013)	< .001	0.082 (0.011)	< .001	-0.009 (0.002)	< .001	**< .001**	[-0.012, -0.006]	-0.040 (0.014)	.005	17.98
	DTI-FA PC	-0.105 (0.013)	< .001	-0.011 (0.011)	.34	0.001 (0.001)	.34	.39	[-0.001, 0.004]	0.059 (0.014)	< .001	1.89
Waist circumference as the mediator												
Neighborhood disadvantage	RSI-RND PC	0.087 (0.012)	< .001	-0.031 (0.011)	.005	-0.003 (0.001)	.009	**.013**	[-0.005, -0.001]	-0.050 (0.013)	< .001	5.07
	RSI-RNI PC	0.083 (0.013)	< .001	0.133 (0.011)	< .001	0.011 (0.002)	< .001	**< .001**	[0.008, 0.015]	0.046 (0.013)	< .001	19.30
	DTI-FA PC	0.086 (0.012)	< .001	0.007 (0.011)	.52	0.001 (0.001)	.53	.58	[-0.001, 0.003]	-0.065 (0.013)	< .001	-0.96
Household income	RSI-RNI PC	-0.048 (0.014)	.001	0.115 (0.011)	< .001	-0.006 (0.002)	.001	**.002**	[-0.009, -0.002]	-0.051 (0.015)	.001	9.89
Parental education	RSI-RND PC	-0.091 (0.013)	< .001	-0.040 (0.011)	< .001	0.004 (0.001)	.001	**.002**	[0.002, 0.006]	0.054 (0.013)	< .001	6.40
	RSI-RNI Fmaj	-0.094 (0.013)	< .001	0.079 (0.011)	< .001	-0.007 (0.001)	< .001	**< .001**	[-0.011, -0.005]	-0.041 (0.014)	.004	15.40
	DTI-FA PC	-0.094 (0.013)	< .001	-0.003 (0.011)	.82	0.000 (0.001)	.81	.85	[-0.002, 0.002]	0.060 (0.014)	< .001	0.41
BMI z-score as the mediator												
Neighborhood disadvantage	RSI-RND PC	0.085 (0.013)	< .001	-0.033 (0.011)	.002	-0.003 (0.001)	.004	**.006**	[-0.005, -0.001]	-0.050 (0.013)	< .001	5.28
	RSI-RNI PC	0.081 (0.013)	< .001	0.139 (0.011)	< .001	0.011 (0.002)	< .001	**< .001**	[0.007, 0.015]	0.046 (0.013)	< .001	19.64
	DTI-FA PC	0.084 (0.013)	< .001	-0.011 (0.011)	.34	-0.001 (0.001)	.32	.39	[-0.003, 0.001]	-0.064 (0.013)	< .001	1.39
Household income	RSI-RNI PC	-0.060 (0.014)	< .001	0.101 (0.011)	< .001	-0.006 (0.002)	< .001	**< .001**	[-0.009, -0.003]	-0.050 (0.015)	.001	10.89
Parental education	RSI-RND PC	-0.088 (0.013)	< .001	-0.050 (0.011)	< .001	0.004 (0.001)	< .001	**< .001**	[0.002, 0.007]	0.053 (0.013)	< .001	7.60
	RSI-RNI Fmaj	-0.090 (0.013)	< .001	0.075 (0.011)	< .001	-0.007 (0.001)	< .001	**< .001**	[-0.010, -0.004]	-0.041 (0.014)	.003	14.02
	DTI-FA PC	-0.090 (0.013)	< .001	-0.023 (0.011)	.04	0.002 (0.001)	.05	.06	[0.000, 0.004]	0.058 (0.014)	< .001	3.45

Abbreviations: CI, confidence interval; DTI, diffusion tensor imaging; DV, dependent variable; FA, fractional anisotropy; IV, independent variable; PC, principal component; RND, restricted normalized directional; RNI, restricted normalized isotropic; RSI, restriction spectrum imaging; SE, standard error; SES, socioeconomic status

[a] Higher levels of obesity-related measures plausibly mediated associations between lower socioeconomic status (SES) and lower RSI-RND and greater RSI-RNI. In each model, one of the three SES indicators was the IV, one obesity-related measure was the mediator, and each of the white matter microstructure PCs that were associated with the SES indicator in question (i.e., IV) was the DV. Models were adjusted for the other two SES indicators (so that independent indirect associations between SES indicators could be assessed), age, sex, PDS, ICV, and mean head motion. False discovery rate (FDR) correction for multiple comparison was applied to all 21 tested indirect associations; results that survived the FDR-corrected threshold of *p*-value ≤ .05 are highlighted. Estimates were standardized *β*'s with standard errors (SEs); 95% confidence intervals (CIs) were estimated using 20000 Monte Carlo simulations. Results were pooled across all imputed datasets. Refer to **eTable 13** in the **Supplement** for sample sizes in individual models.

© 2023 Li ZA et al. *JAMA Network Open*.

eTable 16. Associations Between SES and Cognitive Performance[a]

**Measures (DV)**	**SES indicators (IVs)**					
	**Neighborhood disadvantage** (higher = lower SES)		**Household income** (higher = higher SES)		**Parental education** (higher = higher SES)	
	β **(95% CI)**	***p*-value** (nominal)	***β*** **(95% CI)**	***p*-value** (nominal)	***β*** **(95% CI)**	***p*-value** (nominal)
Total cognition	-0.124 (-0.150 to -0.099)	< .001	0.142 (0.114 to 0.171)	< .001	0.242 (0.215 to 0.268)	< .001
Crystallized cognition	-0.110 (-0.136 to -0.085)	< .001	0.127 (0.098 to 0.156)	< .001	0.261 (0.234 to 0.287)	< .001
Fluid cognition	-0.099 (-0.125 to -0.072)	< .001	0.110 (0.080 to 0.140)	< .001	0.139 (0.111 to 0.167)	< .001
Picture vocabulary	-0.131 (-0.156 to -0.106)	< .001	0.132 (0.104 to 0.161)	< .001	0.233 (0.207 to 0.260)	< .001
Flanker inhibitory control	-0.055 (-0.082 to -0.027)	< .001	0.073 (0.042 to 0.103)	< .001	0.063 (0.034 to 0.091)	< .001
List sorting	-0.100 (-0.126 to -0.074)	< .001	0.113 (0.084 to 0.142)	< .001	0.155 (0.128 to 0.182)	< .001
Dimensional card sort	-0.079 (-0.106 to -0.053)	< .001	0.072 (0.042 to 0.102)	< .001	0.086 (0.058 to 0.114)	< .001
Pattern recognition	-0.052 (-0.079 to -0.025)	< .001	0.033 (0.002 to 0.063)	.04	0.060 (0.032 to 0.089)	< .001
Picture sequencing	-0.043 (-0.070 to -0.017)	.001	0.097 (0.066 to 0.127)	< .001	0.087 (0.059 to 0.115)	< .001
Oral reading	-0.060 (-0.087 to -0.034)	< .001	0.094 (0.064 to 0.124)	< .001	0.220 (0.192 to 0.248)	< .001

Abbreviations: CI, confidence interval; DV, dependent variable; IV, independent variable; SES, socioeconomic status

[a] Higher total cognition composite score (as DV) on the NIH Toolbox Cognition Battery was independently associated with higher socioeconomic status (SES) as indexed by each of the three SES indicators (as IVs). Individual task and crystallized/fluid cognition composite scores were similarly associated with SES in exploratory analyses. Estimates are standardized *β*'s with 95% confidence intervals (CIs). Linear mixed effects models were adjusted for participant age, sex, and PDS and were nested by family. Because we only had one *a priori* hypothesis concerning total cognition and analyses with other cognitive measures were exploratory, multiple comparison correction did not apply.

© 2023 Li ZA et al. *JAMA Network Open*.

eTable 17. Indirect Associations Between SES and White Matter Microstructure via Total Cognition Scores[a]

Model		Path a (IV → mediator)		Path b (Mediator → DV, controlling for IV)		Path a × b (Indirect association)				Path c' (IV → DV, controlling for mediator)		Proportion mediated
**IV**	**DV**	***β* (SE)**	***p*-value** (nominal)	***β* (SE)**	***p*-value** (nominal)	**Estimate (SE)**	***p*-value** (nominal)	***p*-value** (FDR)	**95% CI**	***β* (SE)**	***p*-value** (nominal)	**%**
Total cognition score as the mediator												
Neighborhood disadvantage	RSI-RND PC (*n* = 8498)	-0.126 (0.012)	< .001	0.095 (0.012)	< .001	-0.012 (0.002)	< .001	**< .001**	[-0.016, -0.009]	-0.039 (0.013)	.002	23.59
	DTI-FA PC (*n* = 8533)	-0.126 (0.012)	< .001	0.082 (0.012)	< .001	-0.010 (0.002)	< .001	**< .001**	[-0.014, -0.007]	-0.054 (0.013)	< .001	16.04
Parental education	RSI-RND PC (*n* = 8489)	0.238 (0.013)	< .001	0.080 (0.012)	< .001	0.019 (0.003)	< .001	**< .001**	[0.013, 0.025]	0.038 (0.014)	.006	33.34
	DTI-FA PC (*n* = 8524)	0.238 (0.013)	< .001	0.067 (0.012)	< .001	0.016 (0.003)	< .001	**< .001**	[0.010, 0.022]	0.042 (0.014)	.004	27.58

Abbreviations: CI, confidence interval; DTI, diffusion tensor imaging; DV, dependent variable; FA, fractional anisotropy; IV, independent variable; PC, principal component; RND, restricted normalized directional; RSI, restriction spectrum imaging; SE, standard error; SES, socioeconomic status

[a] Greater total cognition plausibly mediated the associations between higher socioeconomic status (SES) and greater RSI-RND and DTI-FA. In each model, one of the three SES indicators was the independent variable (IV), total cognition score was the mediator, and each of the white matter microstructure PCs that were associated with the SES indicator in question (i.e., IV) was the dependent variable (DV). Models were adjusted for the other two SES indicators (so that independent indirect associations between SES indicators could be assessed), age, sex, PDS, ICV, and mean head motion. False discovery rate (FDR) correction for multiple comparison was applied to all 4 tested indirect associations; results that survived the FDR-corrected threshold of *p*-value ≤ .05 are highlighted. Estimates were standardized *β*'s with standard errors (SEs); 95% confidence intervals (CIs) were estimated using 20000 Monte Carlo simulations. Results were pooled across all imputed datasets. Sample sizes are noted here as both SES indicators and white matter microstructure PCs had missing values

© 2023 Li ZA et al. *JAMA Network Open*.

eTable 18. Fit Indices for Main Structural Equation Models[a]

Model		Path a × b (Indirect association)		
**IV**	**DV**	**CFI**	**RMSEA**	**SRMR**
BMI as the mediator				
Neighborhood disadvantage	RSI-RND PC	0.974	0.061	0.010
	RSI-RNI PC	0.968	0.065	0.010
	DTI-FA PC	0.964	0.063	0.010
Household income	RSI-RNI PC	0.968	0.066	0.010
Parental education	RSI-RND PC	0.975	0.060	0.009
	RSI-RNI Fmaj	0.962	0.064	0.010
	DTI-FA PC	0.965	0.064	0.010
Waist circumference as the mediator				
Neighborhood disadvantage	RSI-RND PC	0.962	0.073	0.011
	RSI-RNI PC	0.954	0.076	0.011
	DTI-FA PC	0.949	0.074	0.011
Household income	RSI-RNI PC	0.955	0.077	0.011
Parental education	RSI-RND PC	0.964	0.073	0.010
	RSI-RNI Fmaj	0.945	0.076	0.011
	DTI-FA PC	0.952	0.075	0.011
BMI z-score as the mediator				
Neighborhood disadvantage	RSI-RND PC	0.953	0.080	0.011
	RSI-RNI PC	0.946	0.081	0.011
	DTI-FA PC	0.938	0.079	0.011
Household income	RSI-RNI PC	0.945	0.082	0.012
Parental education	RSI-RND PC	0.955	0.079	0.011
	RSI-RNI Fmaj	0.934	0.081	0.011
	DTI-FA PC	0.941	0.080	0.011
Total cognition composite score as the mediator				
Neighborhood disadvantage	RSI-RND PC	0.967	0.079	0.012
	DTI-FA PC	0.959	0.079	0.012
Parental education	RSI-RND PC	0.968	0.079	0.012
	DTI-FA PC	0.961	0.079	0.012

Abbreviations: CFI, comparative fit index; DTI, diffusion tensor imaging; DV, dependent variable; FA, fractional anisotropy; IV, independent variable; PC, principal component; RMSEA, root mean square error of approximation; RND, restricted normalized directional; RNI, restricted normalized isotropic; RSI, restriction spectrum imaging; SRMR, standardized root mean square residual

[a] Models demonstrated good fit[25–27] that are comparable to those in reported models[28]. See **eTable 15** and **eTable 17** in the **Supplement** for detailed indirect associations estimates.

© 2023 Li ZA et al. *JAMA Network Open*.

eTable 19. Indirect Associations Between SES and White Matter Microstructure via Cognitive Task Scores[a]

Model		Path a (IV → mediator)		Path b (Mediator → DV, controlling for IV)		Path a × b (Indirect association)		Path c' (IV → DV, controlling for mediator)		Proportion mediated
**IV**	**DV**	***β* (SE)**	***p*-value (nominal)**	***β* (SE)**	***p*-value (nominal)**	**Estimate (SE)**	***p*-value (nominal)**	***β* (SE)**	***p*-value (nominal)**	**%**
Crystallized cognition composite score as the mediator										
Neighborhood disadvantage	RSI-RND PC	-0.099 (0.013)	< .001	0.055 (0.011)	< .001	-0.005 (0.001)	< .001	-0.044 (0.013)	.001	11.07
	DTI-FA PC	-0.098 (0.013)	< .001	0.051 (0.012)	< .001	-0.005 (0.001)	< .001	-0.058 (0.014)	< .001	8.01
Parental education	RSI-RND PC	0.246 (0.013)	< .001	0.040 (0.011)	< .001	0.010 (0.003)	.001	0.046 (0.014)	.001	17.40
	DTI-FA PC	0.246 (0.013)	< .001	0.033 (0.012)	.005	0.008 (0.003)	.005	0.049 (0.014)	.001	14.32
Fluid cognition composite score as the mediator										
Neighborhood disadvantage	RSI-RND PC	-0.091 (0.013)	< .001	0.092 (0.011)	< .001	-0.008 (0.002)	< .001	-0.042 (0.013)	.002	16.73
	DTI-FA PC	-0.092 (0.013)	< .001	0.075 (0.011)	< .001	-0.007 (0.001)	< .001	-0.057 (0.014)	< .001	10.78
Parental education	RSI-RND PC	0.132 (0.014)	< .001	0.082 (0.011)	< .001	0.011 (0.002)	< .001	0.046 (0.014)	.001	19.14
	DTI-FA PC	0.131 (0.014)	< .001	0.071 (0.011)	< .001	0.009 (0.002)	< .001	0.049 (0.014)	.001	16.02
Picture vocabulary task score (assessing verbal knowledge) as the mediator										
Neighborhood disadvantage	RSI-RND PC	-0.122 (0.013)	< .001	0.063 (0.011)	< .001	-0.008 (0.002)	< .001	-0.042 (0.013)	.001	15.35
	DTI-FA PC	-0.120 (0.013)	< .001	0.061 (0.012)	< .001	-0.007 (0.002)	< .001	-0.055 (0.014)	< .001	11.72
Parental education	RSI-RND PC	0.218 (0.013)	< .001	0.046 (0.011)	< .001	0.010 (0.003)	< .001	0.048 (0.014)	< .001	17.32
	DTI-FA PC	0.218 (0.013)	< .001	0.047 (0.012)	< .001	0.010 (0.003)	< .001	0.049 (0.014)	.001	17.36
Flanker inhibitory control task score (assessing attention and executive functioning) as the mediator										
Neighborhood disadvantage	RSI-RND PC	-0.050 (0.014)	< .001	0.042 (0.010)	< .001	-0.002 (0.001)	.006	-0.049 (0.013)	< .001	4.20
	DTI-FA PC	-0.051 (0.014)	< .001	0.028 (0.011)	.007	-0.001 (0.001)	.03	-0.062 (0.014)	< .001	2.31
Parental education	RSI-RND PC	0.058 (0.014)	< .001	0.034 (0.010)	.001	0.002 (0.001)	.009	0.056 (0.013)	< .001	3.47
	DTI-FA PC	0.059 (0.014)	< .001	0.023 (0.010)	.03	0.001 (0.001)	.05	0.058 (0.014)	< .001	2.32
List sorting task score (assessing working memory) as the mediator										
Neighborhood disadvantage	RSI-RND PC	-0.089 (0.013)	< .001	0.051 (0.011)	< .001	-0.005 (0.001)	< .001	-0.046 (0.013)	< .001	8.92
	DTI-FA PC	-0.088 (0.013)	< .001	0.050 (0.011)	< .001	-0.004 (0.001)	< .001	-0.059 (0.014)	< .001	7.02
Parental education	RSI-RND PC	0.146 (0.014)	< .001	0.049 (0.011)	< .001	0.007 (0.002)	< .001	0.050 (0.014)	< .001	12.57
	DTI-FA PC	0.146 (0.014)	< .001	0.044 (0.011)	< .001	0.006 (0.002)	< .001	0.052 (0.014)	< .001	10.90
Dimensional change card sort task score (assessing executive functioning) as the mediator										
Neighborhood disadvantage	RSI-RND PC	-0.074 (0.013)	< .001	0.061 (0.010)	< .001	-0.005 (0.001)	< .001	-0.046 (0.013)	< .001	9.04
	DTI-FA PC	-0.075 (0.013)	< .001	0.051 (0.011)	< .001	-0.004 (0.001)	< .001	-0.059 (0.014)	< .001	6.07
Parental education	RSI-RND PC	0.081 (0.014)	< .001	0.057 (0.010)	< .001	0.005 (0.001)	< .001	0.053 (0.013)	< .001	8.03
	DTI-FA PC	0.081 (0.014)	< .001	0.051 (0.011)	< .001	0.004 (0.001)	< .001	0.054 (0.014)	< .001	7.08
Pattern comparison task score (assessing processing speed) as the mediator										
	RSI-RND PC	-0.048 (0.014)	< .001	0.072 (0.010)	< .001	-0.003 (0.001)	.002	-0.048 (0.013)	< .001	6.87

© 2023 Li ZA et al. *JAMA Network Open*.

Neighborhood disadvantage	DTI-FA PC	-0.049 (0.014)	< .001	0.048 (0.011)	< .001	-0.002 (0.001)	.004	-0.061 (0.014)	< .001	3.79
Parental education	RSI-RND PC	0.059 (0.014)	< .001	0.066 (0.010)	< .001	0.004 (0.001)	< .001	0.054 (0.013)	< .001	6.73
	DTI-FA PC	0.057 (0.014)	< .001	0.047 (0.011)	< .001	0.003 (0.001)	.003	0.056 (0.014)	< .001	4.53
Picture sequence task score (assessing episodic memory) as the mediator										
Neighborhood disadvantage	RSI-RND PC	-0.040 (0.013)	.003	0.049 (0.010)	< .001	-0.002 (0.001)	.01	-0.049 (0.013)	< .001	3.94
	DTI-FA PC	-0.040 (0.013)	.003	0.047 (0.011)	< .001	-0.002 (0.001)	.01	-0.062 (0.014)	< .001	2.95
Parental education	RSI-RND PC	0.086 (0.014)	< .001	0.039 (0.010)	< .001	0.003 (0.001)	.001	0.055 (0.013)	< .001	5.78
	DTI-FA PC	0.085 (0.014)	< .001	0.044 (0.011)	< .001	0.004 (0.001)	.001	0.056 (0.014)	< .001	6.24
Oral reading task score (assessing reading ability) as the mediator										
Neighborhood disadvantage	RSI-RND PC	-0.050 (0.013)	< .001	0.033 (0.011)	.002	-0.002 (0.001)	.02	-0.050 (0.013)	< .001	3.24
	DTI-FA PC	-0.050 (0.013)	< .001	0.029 (0.011)	.009	-0.001 (0.001)	.03	-0.063 (0.014)	< .001	2.22
Parental education	RSI-RND PC	0.211 (0.014)	< .001	0.022 (0.011)	.04	0.005 (0.002)	.04	0.054 (0.014)	< .001	8.01
	DTI-FA PC	0.211 (0.014)	< .001	0.012 (0.011)	.29	0.002 (0.002)	.29	0.057 (0.014)	< .001	4.15

Abbreviations: CI, confidence interval; DTI, diffusion tensor imaging; DV, dependent variable; FA, fractional anisotropy; IV, independent variable; PC, principal component; RND, restricted normalized directional; RSI, restriction spectrum imaging; SE, standard error; SES, socioeconomic status

[a] In exploratory analyses, the independent associations between higher socioeconomic status (SES) indicators and greater RSI-RND and DTI-FA were plausibly mediated by cognitive performance that appeared to be general rather than domain-specific, as similar findings were observed across individual task and fluid/crystallized composite cognitive measures. In each model, one of the three SES indicators was the independent variable (IV), cognition was the mediator, and each of the white matter microstructure PCs that were associated with the SES indicator (i.e., IV) was the dependent variable (DV). Models were adjusted for the other two SES indicators (so that independent indirect associations between SES indicators could be assessed), age, sex, PDS, ICV, and mean head motion. We did not apply multiple comparison correction due to the explorative nature of analyses. Estimates were standardized β's with standard errors (SEs). Results were pooled across all imputed datasets.

© 2023 Li ZA et al. *JAMA Network Open*.

eTable 20. Indirect Associations Between SES and Cognitive Performance via White Matter Microstructure[a]

1) Indirect associations via total cognition score

**Model**		**Path a (IV → mediator)**		**Path b (Mediator → DV, controlling for IV)**		**Path a × b (Indirect association)**				**Path c' (IV → DV, controlling for mediator)**		**Proportion mediated**
**IV**	**Mediator**	***β*** **(SE)**	***p*-value** (nominal)	***β*** **(SE)**	***p*-value** (nominal)	**Estimate (SE)**	***p*-value** (nominal)	***p*-value** (FDR)	**95% CI**	***β*** **(SE)**	***p*-value** (nominal)	**%**
Total cognition composite score as the DV												
Neighborhood disadvantage	RSI-RND PC (*n* = 8498)	-0.050 (0.013)	< .001	0.085 (0.010)	< .001	-0.004 (0.001)	< 0.001	**< .001**	[-0.007, -0.002]	-0.109 (0.012)	< .001	3.74
	DTI-FA PC (*n* = 8533)	-0.064 (0.013)	< .001	0.068 (0.010)	< .001	-0.004 (0.001)	< 0.001	**< .001**	[-0.007, -0.002]	-0.109 (0.012)	< .001	3.82
Parental education	RSI-RND PC (*n* = 8489)	0.056 (0.014)	< .001	0.071 (0.010)	< .001	0.004 (0.001)	< 0.001	**< .001**	[0.002, 0.006]	0.224 (0.013)	< .001	1.76
	DTI-FA PC (*n* = 8524)	0.057 (0.014)	< .001	0.057 (0.010)	< .001	0.003 (0.001)	0.001	**.001**	[0.002, 0.005]	0.224 (0.013)	< .001	1.42

2) Indirect associations via individual task and fluid/crystallized cognition scores

**Model**		**Path a (IV → mediator)**		**Path b (Mediator → DV, controlling for IV)**		**Path a × b (Indirect association)**		**Path c' (IV → DV, controlling for mediator)**		**Proportion mediated**
**IV**	**Mediator**	***β*** **(SE)**	***p*-value** (nominal)	***β*** **(SE)**	***p*-value** (nominal)	**Estimate (SE)**	***p*-value** (nominal)	***β*** **(SE)**	***p*-value** (nominal)	**%**
Crystallized cognition composite score as the DV										
Neighborhood disadvantage	RSI-RND PC	-0.049 (0.013)	< .001	0.050 (0.010)	< .001	-0.002 (0.001)	.003	-0.097 (0.012)	< .001	2.46
	DTI-FA PC	-0.063 (0.013)	< .001	0.043 (0.010)	< .001	-0.003 (0.001)	.002	-0.095 (0.012)	< .001	2.78
Parental education	RSI-RND PC	0.056 (0.014)	< .001	0.036 (0.010)	.001	0.002 (0.001)	.008	0.244 (0.013)	< .001	0.83
	DTI-FA PC	0.057 (0.014)	< .001	0.028 (0.010)	.005	0.002 (0.001)	.02	0.245 (0.013)	< .001	0.65
Fluid cognition composite score as the DV										
Neighborhood disadvantage	RSI-RND PC	-0.050 (0.013)	< .001	0.092 (0.011)	< .001	-0.005 (0.001)	< .001	-0.086 (0.013)	< .001	5.09
	DTI-FA PC	-0.064 (0.013)	< .001	0.070 (0.011)	< .001	-0.004 (0.001)	< .001	-0.087 (0.013)	< .001	4.89
Parental education	RSI-RND PC	0.057 (0.014)	< .001	0.084 (0.011)	< .001	0.005 (0.001)	< .001	0.127 (0.014)	< .001	3.62
	DTI-FA PC	0.058 (0.014)	< .001	0.068 (0.011)	< .001	0.004 (0.001)	.001	0.127 (0.014)	< .001	3.00
Picture vocabulary task score (assessing verbal knowledge) as the DV										
Neighborhood disadvantage	RSI-RND PC	-0.050 (0.013)	< .001	0.057 (0.010)	< .001	-0.003 (0.001)	.001	-0.119 (0.012)	< .001	2.36
	DTI-FA PC	-0.063 (0.013)	< .001	0.051 (0.010)	< .001	-0.003 (0.001)	< .001	-0.117 (0.012)	< .001	2.68
Parental education	RSI-RND PC	0.058 (0.014)	< .001	0.042 (0.010)	< .001	0.002 (0.001)	.003	0.216 (0.013)	< .001	1.13
	DTI-FA PC	0.059 (0.014)	< .001	0.041 (0.010)	< .001	0.002 (0.001)	.003	0.216 (0.013)	< .001	1.10
Flanker inhibitory control task score (assessing attention and executive functioning) as the DV										
Neighborhood disadvantage	RSI-RND PC	-0.051 (0.013)	< .001	0.047 (0.011)	< .001	-0.002 (0.001)	.005	-0.048 (0.013)	< .001	4.73
	DTI-FA PC	-0.063 (0.013)	< .001	0.029 (0.011)	.008	-0.002 (0.001)	.02	-0.049 (0.013)	< .001	3.66

© 2023 Li ZA et al. *JAMA Network Open*.

Parental education	RSI-RND PC	0.058 (0.014)	< .001	0.038 (0.011)	.001	0.002 (0.001)	.009	0.056 (0.014)	< .001	3.81
	DTI-FA PC	0.059 (0.014)	< .001	0.025 (0.011)	.03	0.001 (0.001)	.05	0.057 (0.014)	< .001	2.50
List sorting task score (assessing working memory) as the DV										
Neighborhood disadvantage	RSI-RND PC	-0.051 (0.013)	< .001	0.051 (0.011)	< .001	-0.003 (0.001)	.003	-0.087 (0.013)	< .001	2.90
	DTI-FA PC	-0.064 (0.013)	< .001	0.047 (0.010)	< .001	-0.003 (0.001)	.001	-0.085 (0.013)	< .001	3.40
Parental education	RSI-RND PC	0.057 (0.014)	< .001	0.050 (0.011)	< .001	0.003 (0.001)	.002	0.143 (0.014)	< .001	1.95
	DTI-FA PC	0.059 (0.014)	< .001	0.042 (0.011)	< .001	0.002 (0.001)	.004	0.143 (0.014)	< .001	1.69
Dimensional change card sort task score (assessing executive functioning) as the DV										
Neighborhood disadvantage	RSI-RND PC	-0.050 (0.013)	< .001	0.066 (0.011)	< .001	-0.003 (0.001)	.001	-0.071 (0.013)	< .001	4.45
	DTI-FA PC	-0.063 (0.013)	< .001	0.051 (0.011)	< .001	-0.003 (0.001)	.001	-0.072 (0.013)	< .001	4.28
Parental education	RSI-RND PC	0.057 (0.014)	< .001	0.062 (0.011)	< .001	0.004 (0.001)	.001	0.077 (0.014)	< .001	4.41
	DTI-FA PC	0.058 (0.014)	< .001	0.052 (0.011)	< .001	0.003 (0.001)	.002	0.078 (0.014)	< .001	3.76
Pattern comparison task score (assessing processing speed) as the DV										
Neighborhood disadvantage	RSI-RND PC	-0.051 (0.013)	< .001	0.079 (0.011)	< .001	-0.004 (0.001)	.001	-0.044 (0.013)	.001	8.33
	DTI-FA PC	-0.063 (0.013)	< .001	0.049 (0.011)	< .001	-0.003 (0.001)	.001	-0.046 (0.013)	< .001	6.37
Parental education	RSI-RND PC	0.058 (0.014)	< .001	0.073 (0.011)	< .001	0.004 (0.001)	< .001	0.055 (0.014)	< .001	7.19
	DTI-FA PC	0.059 (0.014)	< .001	0.048 (0.011)	< .001	0.003 (0.001)	.002	0.054 (0.014)	< .001	4.99
Picture sequence task score (assessing episodic memory) as the DV										
Neighborhood disadvantage	RSI-RND PC	-0.051 (0.013)	< .001	0.053 (0.011)	< .001	-0.003 (0.001)	.003	-0.038 (0.013)	.004	6.74
	DTI-FA PC	-0.063 (0.013)	< .001	0.047 (0.011)	< .001	-0.003 (0.001)	.001	-0.037 (0.013)	.005	7.50
Parental education	RSI-RND PC	0.059 (0.014)	< .001	0.043 (0.011)	< .001	0.003 (0.001)	.004	0.083 (0.014)	< .001	2.94
	DTI-FA PC	0.060 (0.014)	< .001	0.045 (0.011)	< .001	0.003 (0.001)	.003	0.083 (0.014)	< .001	3.13
Oral reading task score (assessing reading ability) as the DV										
Neighborhood disadvantage	RSI-RND PC	-0.052 (0.013)	< .001	0.033 (0.011)	.002	-0.002 (0.001)	.02	-0.049 (0.013)	< .001	3.48
	DTI-FA PC	-0.065 (0.013)	< .001	0.027 (0.010)	.01	-0.002 (0.001)	.03	-0.048 (0.013)	< .001	3.55
Parental education	RSI-RND PC	0.059 (0.014)	< .001	0.023 (0.010)	.04	0.001 (0.001)	.07	0.210 (0.014)	< .001	0.62
	DTI-FA PC	0.059 (0.014)	< .001	0.011 (0.011)	.29	0.001 (0.001)	.31	0.210 (0.014)	< .001	0.31

Abbreviations: CI, confidence interval; DTI, diffusion tensor imaging; DV, dependent variable; FA, fractional anisotropy; IV, independent variable; PC, principal component; RND, restricted normalized directional; RSI, restriction spectrum imaging; SE, standard error; SES, socioeconomic status

[a] The independent associations between higher socioeconomic status (SES) and better cognitive performance were plausibly mediated by greater RSI-RND and DTI-FA. Cognitive performance was primarily assessed using the total cognition composite score (**panel 1**), supplemented by exploratory analyses on individual task scores (**panel 2**). Findings again appear general rather than domain-specific. In each model, one of the three SES indicators was the independent variable (IV), each of the white matter microstructure PCs that were associated with the SES indicator in question (i.e., IV) was the mediator, and cognition was the dependent variable (DV). Models were adjusted for the other two SES indicators (so that independent indirect associations between SES indicators could be assessed), age, sex, PDS, ICV, and mean head motion. False discovery rate (FDR) correction for multiple comparison was applied to 4 tested indirect associations involving the total cognition composite score. Results that survived the FDR-corrected threshold of *p*-value ≤ .05 are highlighted. Estimates were standardized β's with standard errors (SEs); 95% confidence intervals (CIs) were estimated using 20000 Monte Carlo simulations. Results were pooled across all imputed datasets. Sample sizes are noted here as both SES indicators and white matter microstructure PCs had missing values.

© 2023 Li ZA et al. *JAMA Network Open*.

eTable 21. Indirect Associations in Random Subsample With 1 Participant per Family[a]

1) Indirect associations via obesity-related measures

**Model**		**Path a (IV → mediator)**		**Path b (Mediator → DV, controlling for IV)**		**Path a × b (Indirect association)**				**Path c' (IV → DV, controlling for mediator)**		**Proportion mediated**
**IV**	**DV**	***β*** **(SE)**	***p*-value (nominal)**	***β*** **(SE)**	***p*-value (nominal)**	**Estimate (SE)**	***p*-value (nominal)**	***p*-value (FDR)**	**95% CI**	***β*** **(SE)**	***p*-value (nominal)**	***%***
BMI as the mediator												
Neighborhood disadvantage	RSI-RND PC (*n* = 7598)	0.107 (0.013)	< .001	-0.030 (0.012)	.010	-0.003 (0.001)	.010	**.02**	[-0.006, -0.001]	-0.051 (0.013)	< .001	5.95
	RSI-RNI PC (*n* = 7526)	0.104 (0.013)	< .001	0.149 (0.012)	< .001	0.015 (0.002)	< .001	**< .001**	[0.011, 0.020]	0.041 (0.014)	.003	27.52
	DTI-FA PC (*n* = 7626)	0.106 (0.013)	< .001	0.004 (0.012)	.77	0.000 (0.001)	.76	.80	[-0.002, 0.003]	-0.066 (0.014)	< .001	-0.57
Household income	RSI-RNI PC (*n* = 7488)	-0.069 (0.015)	< .001	0.127 (0.012)	< .001	-0.009 (0.002)	< .001	**< .001**	[-0.013, -0.005]	-0.042 (0.016)	.008	17.41
Parental education	RSI-RND PC (*n* = 7592)	-0.099 (0.014)	< .001	-0.042 (0.012)	< .001	0.004 (0.001)	.001	**.002**	[0.002, 0.007]	0.053 (0.014)	< .001	7.26
	RSI-RNI Fmaj (*n* = 7601)	-0.101 (0.014)	< .001	0.088 (0.012)	< .001	-0.009 (0.002)	< .001	**< .001**	[-0.013, -0.006]	-0.039 (0.015)	.01	18.82
	DTI-FA PC (*n* = 7618)	-0.101 (0.014)	< .001	-0.006 (0.012)	.60	0.001 (0.001)	.59	.65	[-0.002, 0.003]	0.062 (0.015)	< .001	1.01
Waist circumference as the mediator												
Neighborhood disadvantage	RSI-RND PC (*n* = 7598)	0.088 (0.013)	< .001	-0.029 (0.012)	.013	-0.003 (0.001)	.02	**.03**	[-0.005, -0.001]	-0.051 (0.013)	< .001	4.72
	RSI-RNI PC (*n* = 7526)	0.084 (0.013)	< .001	0.135 (0.012)	< .001	0.011 (0.002)	< .001	**< .001**	[0.008, 0.016]	0.044 (0.014)	.001	20.52
	DTI-FA PC (*n* = 7626)	0.087 (0.013)	< .001	0.009 (0.012)	.44	0.001 (0.001)	.45	.55	[-0.001, 0.003]	-0.067 (0.013)	< .001	-1.22
Household income	RSI-RNI PC (*n* = 7488)	-0.053 (0.015)	.001	0.118 (0.012)	< .001	-0.006 (0.002)	.001	**.002**	[-0.010, -0.003]	-0.044 (0.016)	.005	12.51
Parental education	RSI-RND PC (*n* = 7592)	-0.089 (0.014)	< .001	-0.039 (0.012)	.001	0.003 (0.001)	.002	**.004**	[0.001, 0.006]	0.053 (0.014)	< .001	6.15
	RSI-RNI Fmaj (*n* = 7601)	-0.091 (0.014)	< .001	0.083 (0.012)	< .001	-0.008 (0.002)	< .001	**< .001**	[-0.011, -0.005]	-0.040 (0.015)	.008	15.99
	DTI-FA PC (*n* = 7618)	-0.091 (0.014)	< .001	-0.002 (0.012)	.85	0.000 (0.001)	.84	.84	[-0.002, 0.002]	0.062 (0.015)	< .001	0.33
BMI z-score as the mediator												
Neighborhood disadvantage	RSI-RND PC (*n* = 7598)	0.083 (0.013)	< .001	-0.029 (0.012)	.01	-0.002 (0.001)	.02	**.02**	[-0.005, -0.001]	-0.052 (0.013)	< .001	4.42
	RSI-RNI PC (*n* = 7526)	0.080 (0.014)	< .001	0.140 (0.012)	< .001	0.011 (0.002)	< .001	**< .001**	[0.007, 0.016]	0.044 (0.014)	.001	20.15
	DTI-FA PC (*n* = 7626)	0.083 (0.013)	< .001	-0.006 (0.012)	.59	-0.001 (0.001)	.57	.65	[-0.003, 0.002]	-0.065 (0.014)	< .001	0.82
Household income	RSI-RNI PC (*n* = 7488)	-0.067 (0.015)	< .001	0.104 (0.012)	< .001	-0.007 (0.002)	< .001	**< .001**	[-0.011, -0.004]	-0.043 (0.016)	.006	13.99

© 2023 Li ZA et al. *JAMA Network Open*.

Parental education	RSI-RND PC (*n* = 7592)	-0.082 (0.014)	< .001	-0.045 (0.012)	< .001	0.004 (0.001)	.001	**.002**	[0.002, 0.007]	0.053 (0.014)	< .001	6.47
	RSI-RNI Fmaj (*n* = 7601)	-0.082 (0.014)	< .001	0.082 (0.012)	< .001	-0.007 (0.002)	< .001	**< .001**	[-0.010, -0.004]	-0.041 (0.015)	.007	14.35
	DTI-FA PC (*n* = 7618)	-0.083 (0.014)	< .001	-0.019 (0.012)	0.11	0.002 (0.001)	.12	.156	[0.000, 0.004]	0.061 (0.015)	< .001	2.53

2) Indirect associations via total cognition score

**Model**		**Path a (IV → mediator)**		**Path b (Mediator → DV, controlling for IV)**		**Path a × b (Indirect association)**				**Path c' (IV → DV, controlling for mediator)**		**Proportion mediated**
**IV**	**DV**	***β* (SE)**	***p*-value (nominal)**	***β* (SE)**	***p*-value (nominal)**	**Estimate (SE)**	***p*-value (nominal)**	***p*-value (FDR)**	**95% CI**	***β* (SE)**	***p*-value (nominal)**	**%**
Total cognition score as the mediator												
Neighborhood disadvantage	RSI-RND PC (*n* = 7327)	-0.129 (0.013)	< .001	0.098 (0.012)	< .001	-0.013 (0.002)	< .001	**< .001**	[-0.017, -0.009]	-0.039 (0.014)	.004	24.46
	DTI-FA PC (*n* = 7354)	-0.129 (0.013)	< .001	0.086 (0.013)	< .001	-0.011 (0.002)	< .001	**< .001**	[-0.015, -0.008]	-0.054 (0.014)	< .001	17.07
Parental education	RSI-RND PC (*n* = 7324)	0.242 (0.014)	< .001	0.083 (0.012)	< .001	0.020 (0.003)	< .001	**< .001**	[0.014, 0.027]	0.036 (0.015)	.02	36.23
	DTI-FA PC (*n* = 7346)	0.242 (0.014)	< .001	0.073 (0.013)	< .001	0.018 (0.003)	< .001	**< .001**	[0.012, 0.024]	0.042 (0.015)	.006	29.86

Abbreviations: BMI, body mass index; CI, confidence interval; DTI, diffusion tensor imaging; DV, dependent variable; FA, fractional anisotropy; IV, independent variable; PC, principal component; RND, restricted normalized directional; RSI, restriction spectrum imaging; SE, standard error; SES, socioeconomic status

[a] Both (**1**) obesity-related measures and (**2**) total cognition plausibly mediated the associations between socioeconomic status (SES) and white matter microstructure. These findings here are all in line with those in main analyses (see **eTable 15** and **eTable 17** in the **Supplement**), ruling out confounding from family structure. In each model, one of the three SES indicators was the independent variable (IV), one obesity-related measure or total cognition score was the mediator, and each of the white matter microstructure PCs that were associated with the SES indicator in question (i.e., IV) was the dependent variable (DV). Models were adjusted for the other two SES indicators (so that independent indirect associations between SES indicators could be assessed), age, sex, PDS, ICV, and mean head motion. False discovery rate (FDR) correction for multiple comparison was applied to each group of 21 tests (**1**) or 4 tests (**2**). Results that survived the FDR-corrected threshold of p-value ≤ .05 are highlighted. Estimates were standardized β's with standard errors (SEs); 95% confidence intervals (CIs) were estimated using 20000 Monte Carlo simulations. Results were pooled across all imputed datasets.

© 2023 Li ZA et al. *JAMA Network Open*.

eReferences

1. Garavan H, Bartsch H, Conway K, et al. Recruiting the ABCD sample: Design considerations and procedures. *Dev Cogn Neurosci*. 2018;32:16-22. doi:10.1016/j.dcn.2018.04.004
2. Casey BJ, Cannonier T, Conley MI, et al. The Adolescent Brain Cognitive Development (ABCD) study: Imaging acquisition across 21 sites. *Dev Cogn Neurosci*. 2018;32:43-54. doi:https://doi.org/10.1016/j.dcn.2018.03.001
3. Li Y, Thompson WK, Reuter C, et al. Rates of Incidental Findings in Brain Magnetic Resonance Imaging in Children. *JAMA Neurol*. 2021;78(5):578-587. doi:10.1001/jamaneurol.2021.0306
4. McCuen-Wurst C, Ruggieri M, Allison KC. Disordered eating and obesity: associations between binge-eating disorder, night-eating syndrome, and weight-related comorbidities. *Ann N Y Acad Sci*. 2018;1411(1):96-105. doi:https://doi.org/10.1111/nyas.13467
5. Liang Y, Hou D, Zhao X, et al. Childhood obesity affects adult metabolic syndrome and diabetes. *Endocrine*. 2015;50(1):87-92. doi:10.1007/s12020-015-0560-7
6. Hackman DA, Cserbik D, Chen J-C, et al. Association of Local Variation in Neighborhood Disadvantage in Metropolitan Areas With Youth Neurocognition and Brain Structure. *JAMA Pediatr*. 2021;175(8):e210426-e210426. doi:10.1001/jamapediatrics.2021.0426
7. Rakesh D, Zalesky A, Whittle S. Similar but distinct – Effects of different socioeconomic indicators on resting state functional connectivity: Findings from the Adolescent Brain Cognitive Development (ABCD) Study®. *Dev Cogn Neurosci*. 2021;51:101005. doi:https://doi.org/10.1016/j.dcn.2021.101005
8. Auchter AM, Hernandez Mejia M, Heyser CJ, et al. A description of the ABCD organizational structure and communication framework. *Dev Cogn Neurosci*. 2018;32:8-15. doi:10.1016/j.dcn.2018.04.003
9. White NS, Leergaard TB, D'Arceuil H, Bjaalie JG, Dale AM. Probing tissue microstructure with restriction spectrum imaging: Histological and theoretical validation. *Hum Brain Mapp*. 2013;34(2):327-346. doi:https://doi.org/10.1002/hbm.21454
10. Palmer CE, Pecheva D, Iversen JR, et al. Microstructural development from 9 to 14 years: Evidence from the ABCD Study. *Dev Cogn Neurosci*. 2022;53:101044. doi:https://doi.org/10.1016/j.dcn.2021.101044
11. Luciana M, Bjork JM, Nagel BJ, et al. Adolescent neurocognitive development and impacts of substance use: Overview of the adolescent brain cognitive development (ABCD) baseline neurocognition battery. *Dev Cogn Neurosci*. 2018;32:67-79. doi:10.1016/j.dcn.2018.02.006
12. Akshoomoff N, Beaumont JL, Bauer PJ, et al. VIII. NIH Toolbox Cognition Battery (CB): composite scores of crystallized, fluid, and overall cognition. *Monogr Soc Res Child Dev*. 2013;78(4):119-132. doi:10.1111/mono.12038
13. Zhu T, Hu R, Qiu X, et al. Quantification of accuracy and precision of multi-center DTI measurements: A diffusion phantom and human brain study. *Neuroimage*. 2011;56(3):1398-1411. doi:https://doi.org/10.1016/j.neuroimage.2011.02.010
14. Fortin J-P, Parker D, Tunç B, et al. Harmonization of multi-site diffusion tensor imaging data. *Neuroimage*. 2017;161:149-170. doi:https://doi.org/10.1016/j.neuroimage.2017.08.047
15. Barch DM, Albaugh MD, Avenevoli S, et al. Demographic, physical and mental health assessments in the adolescent brain and cognitive development study: Rationale and description. *Dev Cogn Neurosci*. 2018;32:55-66. doi:https://doi.org/10.1016/j.dcn.2017.10.010
16. Karcher NR, Niendam TA, Barch DM. Adverse childhood experiences and psychotic-like experiences are associated above and beyond shared correlates: Findings from the adolescent brain cognitive development study. *Schizophr Res*. 2020;222:235-242. doi:10.1016/j.schres.2020.05.045
17. Kuczmarski RJ, Ogden CL, Guo SS, et al. 2000 CDC Growth Charts for the United States: methods and development. *Vital Health Stat 11*. 2002;(246):1-190.
18. Harrison E, Drake T, Ots R. finalfit: Quickly Create Elegant Regression Results Tables and Plots when Modelling. 2022.
19. McCarthy-Jones S, Oestreich LKL, Lyall AE, et al. Childhood adversity associated with white matter alteration in the corpus callosum, corona radiata, and uncinate fasciculus of psychiatrically healthy adults. *Brain Imaging Behav*. 2018;12(2):449-458. doi:10.1007/s11682-017-9703-1
20. Benedetti F, Bollettini I, Radaelli D, et al. Adverse childhood experiences influence white matter microstructure in patients with bipolar disorder. *Psychol Med*. 2014;44(14):3069-3082. doi:DOI: 10.1017/S0033291714000506
21. Barth C, Lonning V, Gurholt TP, Andreassen OA, Myhre AM, Agartz I. Exploring white matter microstructure and the impact of antipsychotics in adolescent-onset psychosis. *PLoS One*. 2020;15(5):e0233684. https://doi.org/10.1371/journal.pone.0233684.
22. Park J, Kang Y, Han K-M, et al. Association Between the C4 Binding Protein Level and White Matter Integrity in Major Depressive Disorder. *Psychiatry Investig*. 2022;19(9):703-711. doi:10.30773/pi.2022.0100
23. Bokobza C, Van Steenwinckel J, Mani S, Mezger V, Fleiss B, Gressens P. Neuroinflammation in preterm babies and autism spectrum disorders. *Pediatr Res*. 2019;85(2):155-165. doi:10.1038/s41390-018-0208-4
24. Revelle WR. psych: Procedures for personality and psychological research. 2017.
25. Hu L-T, Bentler PM. Evaluating model fit. In: *Structural Equation Modeling: Concepts, Issues, and Applications.* Thousand Oaks, CA, US: Sage Publications, Inc; 1995:76-99.
26. Browne MW, Cudeck R. Alternative Ways of Assessing Model Fit. *Sociol Methods Res*. 1992;21(2):230-258. doi:10.1177/0049124192021002005
27. Hooper D, Coughlan J, Mullen M. Structural Equation Modeling: Guidelines for Determining Model Fit. *Electron J Bus Res Methods*. 2007;6.
28. Taylor RL, Cooper SR, Jackson JJ, Barch DM. Assessment of Neighborhood Poverty, Cognitive Function, and Prefrontal and Hippocampal Volumes in Children. *JAMA Netw Open*. 2020;3(11):e2023774-e2023774. doi:10.1001/jamanetworkopen.2020.23774

© 2023 Li ZA et al. *JAMA Network Open*.
