# Supplementary material for: Associations Between Socioeconomic Status, Obesity, Cognition, and White Matter Microstructure in Children
Source: JAMA Netw Open. 2023 Jun 27;6(6):e2320276. doi: 10.1001/jamanetworkopen.2023.20276 (PMC10300724; doi:10.1001/jamanetworkopen.2023.20276)
Supplement: Supplement 2. — Data Sharing Statement [file jamanetwopen-e2320276-s002.pdf]

Data Sharing Statement

Li. Associations Between Socioeconomic Status, Obesity, Cognition, and White Matter Microstructure in Children. *JAMA Netw Open*. Published June 27, 2023. doi:10.1001/jamanetworkopen.2023.20276

Data
Data available: Yes
Data types: Deidentified participant data, Data dictionary
How to access data: Data in this study were from the baseline measurements published in 2021 as part of the ABCD Curated Data Release 4.0, available at DOI: 10.15154/1523041. The ABCD data repository grows and may be modified as more data are collected and processed. This study is available in the NIMH Data Archive (NDA) at DOI: 10.15154/1528516. A data dictionary is shown in eTable 2 in the Supplement.
When available: beginning date: 02-16-2023

Supporting Documents
Document types: None

Additional Information
Who can access the data: Data in this study is available as part of the ABCD Curated Data Release 4.0 at DOI: 10.15154/1523041. Access is open to researchers with valid NIMH Data Archive (NDA) Data Use Certification (DUC).
Types of analyses: Pediatric research purposes.
Mechanisms of data availability: Access is open to approved researchers with valid NIMH Data Archive (NDA) Data Use Certification (DUC).
